# Opportunistic offering of self-sampling to non-attenders within the English cervical screening programme: a pragmatic, multicentre, implementation feasibility trial with randomly allocated cluster intervention start dates (YouScreen)

**DOI:** 10.1016/j.eclinm.2024.102672

**Published:** 2024-07-16

**Authors:** Anita W.W. Lim, Katie Deats, Joanna Gambell, Alexandra Lawrence, Jiayao Lei, Mairead Lyons, Bernard North, Dharmishta Parmar, Hasit Patel, Jo Waller, Jane Warwick, Peter D. Sasieni, YouScreen Joint Steering Group, Alexandra Lawrence, Clare Stephens, Afsana Bhuiya, Fanta Bojang, Catherine Nestor, Naser Turabi, Holly Norman, Kate Sanger, Michelle Quaye, Farhat Gilani, Misha Ladva, Anita Lim, Peter Sasieni, Jo Waller, Mairéad Lyons, Jo Gambell, Katie Deats, Ann-Marie Wright, Aileen Masson, Philippa Pearmain, Ruth Stubbs, Josephine Ruwende, Hasit Patel, Mike Gandy, Paul Roberts, Pauline Fisher, Angela Lydon-Burgan, Lorraine Silver, Kate Ruane, Nick Winfield, Marion Dunn, Georgina Platt, Molly Taylor, Adele Shepherd, Emma Coppini, Alison Cowie, Caroline Cook, Claire Horner, Elliann Fairbairn, Jo Aracena, Wayne Douglas, Lucy McLaughlin, Gali Siegal

**Affiliations:** a School of Cancer and Pharmaceutical Sciences, Faculty of Life Sciences and Medicine, King's College London, London, SE1 9RT, UK; b Centre for Cancer Screening, Prevention and Early Diagnosis, Queen Mary University of London, London, EC1M 6BQ, UK; c Barts Health NHS Trust, Department of Gynaecology, Royal London Hospital, Whitechapel Road, London, E1 1BB, UK; d Health Service Laboratories LLP, Level 8 #, The Halo Building, Mabledon Place, London, WC1H 9AX, UK

**Keywords:** Human papillomavirus, Cervical cancer, Cervical intraepithelial neoplasia, Mass screening, Self-sampling

## Abstract

**Background:**

Self-sampling has game-changing potential to tackle the declining participation and inequities seen in many organised cervical screening programmes. Wide variation in uptake between settings and mode of kit offer highlight the importance of local piloting. Furthermore, harnessing the benefits of self-sampling in real-world settings has been surprisingly challenging. The YouScreen study estimated the impact of offering self-sampling to non-attenders within the English Programme and evaluated large-scale opportunistic offering of self-sampling in primary care.

**Methods:**

A pragmatic modified stepped-wedge implementation feasibility trial with randomly-allocated cluster intervention start dates at primary care practices in England (133 participating, 62 non-participating). Eligible women were aged 25–64 years and ≥6 months overdue for screening (“non-attenders”). Between January 13, 2021 and 30 November, 2021 self-sampling kits were distributed to non-attenders via an opportunistic offer in primary care when they consulted for any reason and direct mailout to those unscreened 15-months after routine invitation. Primary outcomes were the proportion of non-attenders screened each month; change in coverage; and uptake (90 days). YouScreen is registered with ISRCTN:12759467.

**Findings:**

8338 women provided self-samples following recruitment between January 13, 2021 and 30 November, 2021. Self-samples were returned from 65.5% (6061/9248) who accepted an opportunistically offered kit and 12.9% (2777/17,604) directly-mailed kits. Responders were representative of the ethnically diverse and deprived underlying non-attendee population (64% ethnic minority groups, 60% from the two most deprived national quintiles). The self-sampling intervention resulted in a 22% (95% CI 18–26) increase in non-attenders screened per month (per-protocol analysis) and 12% (95% CI 9–15) (intention-to-treat analysis). Change in coverage at participating (mean intervention duration 7.5 months) vs non-participating practices was 1.6% (95% CI 0.4–2.8). Adverse effects were not formally collected.

**Interpretation:**

Opportunistically offering self-sampling to under-screened women in primary care could increase coverage in England and potentially reach underserved populations.

**Funding:**

North Central London and North East London Cancer Alliance.

**Research in context**

**Evidence before this study**

A PubMed search of articles published between 1 January 2000 and 1 January 2021 using the search terms “self-sample”; “self-collect”; “self-sampling” AND “HPV OR “human papillomavirus” OR “cervical cancer, screening for cancer, HPV” was conducted prior to the trial start. Numerous studies have shown that home-based speculum-free screening offers women comfort, convenience and control but uptake varies greatly in different countries. In the UK 60% of cervical cancer screening non-attenders visit their general practitioner at least once a year suggests that existing primary care appointments could be leveraged to make an in-person offer opportunistically.

**Added value of this study**

YouScreen provides insights on the first large-scale opportunistic offering of self-sampling within a well-established cervical screening programme in a high-income country. Opportunistic offering of self-sampling to women overdue screening when they attended primary care for any reason elicited almost five-fold higher uptake than direct kit mailout. Offering self-sampling to non-attenders for 7.5 months increased coverage in the organised screening programme by 1.6%, with participants predominantly from ethnic minority and deprived backgrounds.

**Implications of all the available evidence**

Opportunistically offering self-sampling to under-screened women in primary care could be an effective approach for improving cervical screening participation and access amongst non-attenders. Research evidence continues to suggest that self-sampling can promote greater equity and accessibility of screening, including traditionally underserved populations.

## Introduction

Cervical cancer is highly preventable through screening; a key pillar of the World Health Organisation's (WHO) global strategy for elimination by 2100.^1^ Yet, a persistent challenge for organised cervical screening programmes is maintaining good coverage.^2^ In England, attendance is in longstanding decline and is particularly sub-optimal in London (age-appropriate coverage 62.3% vs 69.9% nationally).^2^ The importance of having a well-screened population is underscored by the fact that most cases arise in under-screened women.^3^ The main deterrent to conventional screening is the speculum-based pelvic examination which can be physically, emotionally and culturally challenging for women.^4^^,^^5^ Busy lifestyles and difficulty getting appointments are also influential.^5^ Self-sampling overcomes most of these barriers by enabling women to collect their own sample (at home) for human papillomavirus (HPV) testing with comparable accuracy to clinician-taken samples.^6^

Several countries^7^ have already integrated self-sampling as a strategy to improve coverage. Experience from these countries indicates that uptake amongst non-attenders is variable (∼8–17%), setting-specific, and easily influenced by intricacies of how the offer is made.^8^, ^9^, ^10^ This provides rationale for local piloting prior to broader implementation to ensure non-attenders can be reliably and accurately identified, and the mode of self-sampling offer is sufficiently appealing to the target population. The most commonly studied approaches for offering kits are (i) sending letters inviting women to order kits and (ii) sending kits directly to women. In England, an estimated 60% of cervical screening non-attenders consult their general practitioner (GP) at least once a year,^11^ and could be opportunistically offered self-sampling. Electronic GP patient record systems can be programmed to “flag” consulting non-attenders using an on-screen message. This approach has elicited 45%–78%^12^^,^^13^ uptake in small studies but has yet to be evaluated at scale within an organised screening programme.

YouScreen embedded self-sampling into the English Cervical Screening Programme (CSP) for the first time. The trial was designed to test the end-to-end pathways for integrating self-sampling for non-attenders into the national programme and to estimate the associated increase in uptake and coverage. Secondary objectives were to estimate the cervical intraepithelial neoplasia grade 2 or worse (CIN2+) detection rate and the compliance to follow-up for self-sampling HPV-positives. Here, we report on the impact of offering self-sampling to non-attenders in a real-world setting, and the first large scale evaluation of opportunistically offered self-sampling kits in an organised screening programme.

## Methods

### Ethics

All participants provided informed consent prior to taking part (implicit upon return of a self-sample). Ethical approval for the study was granted by the South Birmingham Research Ethics Committee (20/WM/0120), IRAS ID 264776. Approvals were also received from the Confidentiality Advisory Group (20/CAG/0086) and the CSP Research Advisory Committee (CSP-RAC-032).

This study was performed in accordance with the Declaration of Helsinki.

### Cervical screening in England

In England, the National Health Service (NHS) provides free-of-charge cervical screening to all people with a cervix aged 25–64 years. Cervical screening is delivered in GP primary care and uses HPV testing with reflex cytology. A call/recall system is operated via a centralised national database (National Health Application and Infrastructure Services (NHAIS)) which only includes people who are registered as female sex. Invitation letters are sent every three years to women aged 25–49 and every five years to women aged 50–64. A single reminder letter is sent approximately three months after invitation.

### Study design and setting

YouScreen was a pragmatic implementation feasibility clinical trial embedded within the CSP. Study procedures and documents mirrored those of the CSP.

Non-attenders were defined as people aged 25½ to <65 years at least six months overdue for routine cervical screening (i.e., not screened in the last 3½ or 5½ years, depending on age). These women have not responded to their invitation or reminder letter and will not receive further reminders.

All GP practices in the London boroughs of Camden, Islington, Tower Hamlets, Newham and Barnet (see Table S1) were eligible. These areas have low coverage, with highly deprived (59% in the two most deprived national quintiles) and ethnically diverse populations (43.6% ethnic minority groups).^14^ Practices registered interest to participate via an online form publicised in GP forums and newsletters. The impact of the intervention was estimated by comparing screening rates among under-screened women before and after the introduction of the intervention. Data from non-participating practices were used to control for trends in screening activity over time in the absence of the intervention.

Based on previous clinical trials in GP primary care, study set up was planned for an estimated 50% of eligible GP practices (circa. 100) taking part. However, interest from GP practices was much greater than anticipated (n = 164), which required careful planning and management of site set-up to deliver within the study timeline and available resource. An additional challenge was the rapidly changing and pressured environment in GP primary care due to covid-19. GP practices were given a deadline to complete site set up training and to confirm all relevant trial compliances were in place. GP practices which did not respond or which did not complete these activities within the timeframe despite reminders and extensions were removed from the activation plan.

At the individual-level the trial was an open cohort as women could move GP practice, or become eligible (≥6 months overdue) or ineligible (screened during the study) throughout. At the cluster-level, the trial was closed as only practices activated at study start were included and all GP practices agreed to participate before the first practice was randomised.

### Randomisation

Registered GP practices were randomised to a start week between December 2020 and April 2021 making this a “stepped-wedge” design^15^ cluster randomisation. This enabled all registered practices to offer the intervention and facilitated robust assessment by controlling for underlying temporal trends.

The randomisation list was produced using the R programme which implements a random assignment whereby each GP practice was allocated to one of 19 start dates. For the first 18 dates the number of GP practices assigned was alternately 8 and 9 with 6 assigned to the final date making 159 total assignments (two randomised GP practices merged prior to study set up, one with another practice which was randomised and the other merged with a practice which did not register interest/did not participate, leaving a total of 157). Practices were randomised to a start ‘week’ during months 2–6 of the planned active recruitment period (November 2020–October 2021).

Seven “early adopter” practices were excluded from randomisation and were prioritised to start ahead of the main trial to generate lessons and refine study processes ahead of wider roll-out.

GP practices were closed to recruitment in (approximately) reverse order of opening (September to November 2021), with preference given to those with particularly poor recruitment to conserve kit supplies and avoid kit wastage.

Cluster randomisation of when GP practices offered the intervention, meant that we could (1) estimate a GP-specific random effect; (2) estimate temporal effects (influencing all GP practices in the 5 Boroughs over the course of the trial); (3) estimate the causal impact of implementing the intervention on the numbers screened.

### Participant invitation

Self-sampling kits were distributed in two ways: (i) in-person opportunistic offer in GP primary care and (ii) systematic direct mailout from the screening programme so as to compare three elements (1) opportunistic vs systematic; (2) in-person vs direct mailout and (3) primary care vs screening programme.

For the opportunistic offer the GP electronic record system EMIS Web (Egton Medical Information Systems) was programmed to display an on-screen message flagging nonattenders (i.e., those who were at least 6 months overdue cervical screening on the day of the consultation).

Healthcare professionals were asked to offer kits to flagged non-attenders when they consulted for any reason. Women could choose to take their sample in clinic or at home. Self-samples collected in-clinic were returned to the laboratory via routine sample collection services. Home-collected samples were posted by women directly to the laboratory. Women offered self-sampling via remote (telephone or video) consultation were asked to collect kits from the practice. GP practices were asked to post uncollected kits to women's homes after two weeks. All GP practices were instructed to stop opportunistically offering kits no later than 30 November 2021.

Direct-mailout provided a systematic approach for kit distribution and a “mop-up” for nonattenders who did not consult their GP. At the beginning of each month, a pre-programmed search of the national screening database identified females from participating practices who were 15 months overdue for screening. By definition, these individuals received a screening invitation between ≥15 and <16 months earlier and had not been screened for at least 3.5 years. A pre-notification letter was sent to women's homes, followed by a kit about a week later. The 15-month timepoint provided a screening offer that was distinct from both the last reminder and the next routine invitation. Prior consent was not sought as the offer was made as a clinical service. However, a study-specific mechanism enabled women to opt-out of receiving mailed kits and data sharing. The direct-mailout pathway ended in September 2021 due to NHS plans to decommission NHAIS.

### Self-sampling kits

Self-sampling kits contained a flocked swab (552C.80 FLOQswab™, Copan Italia, Brescia, Italy), written and pictorial instructions, a patient information booklet, a pre-paid envelope pre-addressed to the laboratory and a study questionnaire (see supplement).

Participant-facing materials were co-developed with the Cervical Screening Programme and with Public and Patient Representative input. The overall approach was broadly consistent with the Capability Opportunity Motivation-B model of behaviour change. Invitation letters and the patient information booklet included a link to the study microsite and a study helpline (hosted by a cervical cancer charity https://www.jostrust.org.uk/). A microsite provided translated patient information booklet for the main non-English languages spoken in the boroughs (Turkish, Bengali, Polish, Arabic and Somali), frequently asked questions, and an animated instructional video https://www.smallc.org.uk/get-involved/get-involved-youscreen/. The Small c website is fully accessible including a translation tool for over 100 languages and text to speech functionality for 30 languages. An online communications campaign targeting the five boroughs was used to boost credibility of self-sampling and maximise uptake.

Direct mailout kits included a laboratory request consent form pre-filled with the women's details. Opportunistic kits included a plastic specimen bag to facilitate return of samples with the laboratory request consent form via the usual screening collection services. Study questionnaires comprised a single side of A4 in a self-seal freepost envelope and inquired about women's previous barriers to participation.

### Clinical management

Results were reported via the usual CSP routes (letters to women and electronically to GPs). Women with an HPV negative self-sample had their recall date reset to 3 or 5 years (if aged 25–49 or 50–64 years, respectively) as per routine practice following an HPV negative clinician-taken sample. Women with an HPV positive self-sample had their recall reset to 3 months and were advised to have a clinician-taken follow-up test (HPV primary screening test). Further management was then determined by the clinician-taken result according to the HPV primary screening pathway.^16^ This meant that women HPV positive on a self-sample and HPV negative on a follow-up sample were returned to routine screening. At the end of the study, GP practices were asked to make a final attempt to offer a clinician-taken sample to women testing HPV positive on a self-sample who had not attended.

Women with an invalid/insufficient result (i.e., unable to produce a valid result due to insufficient sample cellularity or analytical issues) were sent a repeat kit to their home. Repeat kits were also sent to women whose self-samples were rejected for analysis due to insufficient information supplied.

### Outcomes

The primary outcomes were (1) coverage: change in age-appropriate coverage before and after the intervention at participating vs non-participating practices; (2) activity: change in the number of women at least 6 months overdue who were screened each month (associated with the randomised intervention) and (3) uptake: return of self-sampling kits within 90 days of offer.

Secondary outcomes were compliance to follow-up (clinician screening or colposcopy) at 6 months for screen-positive women and CIN2+ yield.

Implementation endpoints including an assessment of the impact adaptations in response to the COVID-19 pandemic and the impact of social distancing on the trial will be described in detail in a separate paper.

### Laboratory and sample handling

All self-samples were analysed by the designated NHS laboratory for cervical screening in London (Health Services Laboratory). Samples were transported dry at ambient temperature and were analysed for the presence of high-risk HPV using Roche cobas 4800 (Roche Diagnostics GmBH) according to the manufacturer's instruction. Upon arrival at the laboratory, swabs were immediately resuspended in 5 mL of PreservCyt media (Hologic, USA) and vortexed for 10 s in the original sample tube with the flocked swab immersed. Samples were stored at 5 °C until the next run, at which point they were aliquoted into another tube that was loaded onto the Roche cobas 4800 for analysis. The Roche cobas 4800 assay specifically identifies (types) HPV16 and HPV18 while concurrently detecting the 12 other high-risk types (31, 33, 35, 39, 45, 51, 52, 56, 58, 59, 66 and 68) at clinically relevant infection levels. The Cobas® assay simultaneously tests for human beta-globin as an internal control of sufficient specimen cellularity. In addition, a positive and negative control specimen were included in each run.

Roche cobas 4800 is not licensed for HPV testing on self-collected dry vaginal swab samples. The NHS England Screening Programme gave approval for off-license use of Roche cobas 4800 in YouScreen on the basis that (i) women were clearly informed in the information leaflet that a clinician-taken sample was most effective; (ii) women gave informed consent to participate (implicit upon return of a self-sample) and (iii) given that this was a non-attender population, a slightly inferior screen is better than no screening.

Self-samples received more than 14 days after the sample taking date were reported as invalid/insufficient unless HPV positive. This was mandated by the Cervical Screening Programme in the absence of their own studies sample stability to minimise potential false negative reporting due to sample degradation.

Clinician-taken follow-up samples were collected and analysed as per routine screening using ThinPrep (Hologic, Marlborough, MA) and the APTIMA (Hologic, Manchester) system.

### Data collection and sources

YouScreen collected a series of cross-sectional individual-level and aggregate data from different data sources. Confidentiality Advisory Group approval (20/CAG/0086) enabled collection of de-identified data on all eligible women including those who did not return a sample. Individual-level data were obtained from (i) NHAIS (national screening records); (ii) laboratory (HPV test results); (iii) Cyres Colposcopy database linked via the laboratory (HPV primary test results, cytology tests, colposcopy data including histology); (iv) GP records and (v) print company (invitation letters, direct mailout and repeat kits). Datasets were linked via an encrypted version of the unique personal identifier (NHS number) for NHS health records. Encryption was via OpenPseudonymiser (https://www.openpseudonymiser.org/Default.aspx) a free open-source software.

NHAIS individual-level data were used to classify women according to their screening history and quintile of the Index of Multiple Deprivation 2019 (IMD) derived from residential postcode. GP individual-level data provided women's ethnicity and details of the opportunistic offer (date of offer, type of consultation and the type of healthcare professional offering kits). Histological outcomes (the most severe for each woman) were extracted by the laboratory from the Cyres colposcopy database which holds data from all NHS colposcopy clinics in London.

Individual-level data were unavailable for some women in various datasets. England operates a National Data Opt-Out (NDOO) whereby individuals can opt-out of having their health data being used for secondary purposes.^17^ Eligible individuals could also opt out of data sharing for the study via their GP practice. A study-specific code was entered into the electronic patient record which automatically removed individuals with this code in GP-based trial reporting datasets (apart from aggregate reports which did not require permission). GP practices sent details of each person who opted out (full name, date of birth, NHS number and GP practice) to a trial-specific email address managed by the Cervical Screening Administration Services (CSAS). Throughout the trial, CSAS provided a regularly updated list of opted-out individuals to NHS Digital (via a secure support ticket system).

Individual-level data from GP records were unavailable for women in the NDOO and from five participating practices that had technical issues excluding those in the NDOO from the data. These five GP practices were still included in the study as all other datasets were available (including aggregate GP-practice data). NHAIS and GP individual-level data were also unavailable from women in the study-specific opt-out (50 of approximately 147,965, 0.03%). Additionally, as NHAIS, GP and Cyres records are live databases and data were extracted several months after trial end, data may be missing for women who relocated or GP practices which closed during the trial.

Two aggregate NHAIS datasets were obtained for all 195 GP practices in the YouScreen boroughs. The first comprised the standard CSP reports for age-appropriate coverage for each practice and each month between December 2020 and April 2022. The second comprised counts for each practice of the number of non-attenders (≥6 months overdue screening) on the first of the calendar month, and of these, numbers screened during that month (by self-sample or conventional screening). Data were extracted for September 2020–April 2022.

Data on gender are not routinely collected by the NHS Cervical Screening Programme or in primary care records and were therefore not available in the dataset for analysis. All individuals in the datasets were presumed to be of female sex given that (i) the NHAIS database only includes females and (ii) cervical screening is for people with a cervix.

### Safety reporting

The study protocol did not stipulate that adverse events would be formally reported or collected as self-sampling is a low-risk intervention and flocked swabs for vaginal self-collection are already widely used within the NHS. We did not anticipate any major safety issues based on previous studies and experience.

There was a very small risk that the swab could break off in the woman's vagina during sampling as there is a breakpoint at 8 cm from the swab tip. The participant information booklet advised women to report this to their GP who were in turn asked to inform the trial team via email or telephone.

### Statistics

Demographic variables and screening history for women who returned a self-sample are presented using standard descriptive methods. Proportions were calculated with associated Wilson confidence intervals.

Screening history was classified according to the time from last adequate NHS test before kit offer or return as (1) ‘not overdue’; (2) ‘late’—overdue by 6–24 months, (3) ‘very late’—overdue by at least 24 months, and (4) ‘never’—no adequate NHS tests recorded. Women aged 25–27 years with no tests recorded were classified as ‘late’, rather than ‘never’. For self-sample screen-positive women, follow-up investigation was defined as having either a clinician-taken sample or colposcopy visit within six months. Baseline CIN2+ yield was defined as those with CIN2 or worse (including invasive cancer) as a proportion of all women with a valid self-sample result. CIN2+ identified at early recall (at 12 months) for HPV positive/cytology negative follow-up results was excluded.

Deprivation was assessed using the standard measure of relative deprivation in England (IMD). Women's postcodes in NHAIS were converted to IMD quintiles in the English indices of deprivation report for 2019 using Lower Layer Super Output Area. Ethnicity codes from GP records were grouped according to the Office for National Statistics categories.

Box 1 shows a summary of comparators and analyses for primary endpoints. We estimated coverage at each GP practice, before (December 2020) and after (February 2022) the intervention, using routinely collected aggregate NHAIS data and the standard CSP definition of coverage: the proportion eligible for screening who were screened adequately within 3.5 years for those aged 25–49 years, and within 5.5 years for those aged 50–64 years. To assess the impact of the intervention, we compared the change in coverage (with 95% Wald confidence intervals) between December 2020 and February 2022 in participating vs the non-participating (“control”) GP practices, using weighted linear regression (each practice weighted by the inverse of the expected variance of its coverage before the intervention) to downweigh practices with small numbers. GP practices were weighted by min (nDecember 2020, nFebruary 2022)/[COVdec20∗(1- COVdec20)], which is based on the reciprocal of the square of the standard error of the √(n/(pq)) where n is the sample size and p an estimate of a proportion and q = 1-p and n is taken as the minimum of the number eligible in December 2020 and February 2022 (nDecember 2020 and nFebruary 2022).

Box 1 **Summary of comparators and analyses for primary endpoints in the YouScreen trial.**

**Table:** 

Metric	Basis for comparison	Analysis
**Coverage** (as defined by the screening programme)	Quasi-experimental design: Difference of differences	Comparison of change in coverage between December 2020 and February 2022 between participating and non-participating GP practices
**Activity**: Proportion of women ≥6 months overdue screening (i.e., nonattenders) screened each month	Randomised controlled clinical trial	Estimation of the (fixed) effect of the intervention on screening of nonattender women using a mixed-effects Poisson regression model
**Uptake** of self-sampling following an offer	Observation in those offered self-sampling as part of the trial intervention	Proportion of those offered self-sampling who returned a valid sample. Estimated separately by type of offer (direct mailout or opportunistic), screening history, and various demographics

As coverage is measured over 3.5–5.5 years whereas the intervention was at most one year, and to take advantage of the randomisation, we additionally analysed the number of non-attenders screened each month, per practice, from September 2020 to April 2022. We fitted a log-linear mixed-effects model with Poisson distribution. We used the logarithm of the number at least 6 months overdue screening on the first of the month as an offset and included a random effect for GP practices and a fixed effect for calendar months. A fixed effect for GP-practice-months in which self-sampling was offered by the practice throughout the month, and another for months in which it was offered for just part of the month were estimated.

For hypothesis testing, we performed a randomised comparison (intention-to-treat) which excluded the early adopters and used the randomised start dates and allocated end dates (as opposed to the actual intervention start and end dates).

As the intervention started several weeks after the randomised start date at some practices, we additionally conducted a per-protocol (PP) analysis to estimate the magnitude of the intervention effect using the actual intervention start and end dates. PP analysis included the early-adopter practices and used the actual intervention start and end date (defined as the first offer in the last month in which at least 3 opportunistic kit offers were made or the final direct mailout (30 September 2021), whichever was latest). This provides a controlled parallel interrupted time-series design, one of the strongest quasi-experimental designs. It also distinguishes between months in which GP practices used both kit pathways and months in which either opportunistic or direct mailout were used. An NHAIS programming issue resulted in no direct-mailout kits being sent for 32 participating GP practices, enabling the impact on coverage for each pathway to be estimated.

We estimated the total additional number of non-attenders screened due to the intervention by comparing the observed numbers in participating practices with the estimated counterfactual number in the absence of any intervention using the formula N (1–1/R), where N is the observed number and R is the rate ratio associated with the intervention (PP analysis).

The model used for the ITT to estimate the effect of the intervention on the monthly count of nonattenders screened was:

$$\log\;{\text{ES}}_{\text{mg}}=\log\left(N_{\text{mg}}\right)+\beta_{1}*I_{\text{mg}}+\beta_{2}*J_{\text{mg}}+T_{m}+P_{g}$$

• whereES_mg_ is the expected number of eligible women adequately screened at practice g in month m
• N_mg_ is an offset (the number of women≥6 m overdue screening at the start of month m in practice g),
• T_m_ is a fixed month effect treated as nominal, starting with the first month we have screening data
• P_g_ is a random effect for practice g
• I_mg_ is an indicator variable equal to 1 for practice g in a month m fully employing the intervention, based on planned start date, and 0 for those months where practice g is not employing the intervention or for which the practice is in a peri-intervention month.

A peri-intervention month is a month where the intervention has a partial effect: for practice g month m is a peri-intervention month if it is the month that contains the planned (randomised) switch-over date to the intervention (unless that occurs on the first of the month), the month that the practice stop-date occurs, and the month following the stop-date.

A peri-intervention month is indicated by the variable J_mg_ taking the value 1 (and 0 for a non-peri-intervention month). S_mg_ is assumed to be a Poisson random variable with mean ES_mg_.

The effect of the intervention after adjusting for month of year, temporal changes in coverage and variation over practices and after treating peri-intervention effects as a nuisance factor, was assessed by β_1_. We anticipated that 0< β_2_< β_1_ and wanted to show that β_1_ is significantly greater than 0.

For the PP the model was similar with the intervention variables I, m and J redefined to reflect the actual intervention. For the ITT the randomised start dates were used, and for the PP the actual start dates were used. In the PP we divided the full intervention variable depending on whether it was the full intervention, direct mailout only, opportunistic only or peri-intervention.

Non-participating practices provide data on two aspects of variability and allow more precise estimation of the treatment effects. This first is a better estimation of the monthly variation in the absence of the intervention. This is important because without that months in which all participating practices were offering the intervention would not contribute to estimating the effect of the intervention. Secondly, having more practices allows for more precise estimation of the random-effects—the variation between practices. Because there is a fixed effect for non-participating practices, and random-effects for all practices, the intervention effect only compares what happens in participating practices in months with the intervention with months without the intervention. As very few participating GP practices offered no intervention between May 2021 and September 2021, without including non-participating GP practices in the ITT analysis, the effect of intervention would be underestimated because there were no sufficient referent practices months without intervention during that period. Including the non-participating GP practices also enabled us to estimate the effect of calendar month.

Uptake was defined as the return of a self-sample following the mailout or offer of a kit and was categorised according to the first kit type received if more than one kit was returned. Uptake at 90 days was calculated separately for kit pathway, as were response by age, ethnicity, IMD and screening history. For the opportunistic offer, this was only possible for women from whom a date of offer in GP individual-level data was available.

We used funnel plots to explore the variability in self-sampling uptake by GP practice separately for each kit pathway.

Statistical analyses were conducted using Stata (version 17.0) and R (version 4.1.1). YouScreen is registered with ISRCTN:12759467.

### Role of the funding source

The funder had a role in writing of the report, approved the design, attended the trial steering committee and was represented by authors on the paper. The funder had no role in study design, data collection, data analysis, data interpretation or the writing of the statistical analysis report. The sponsor had no role in study design, data collection, data analysis, data interpretation, writing of the report or the decision to submit the article for publication.

## Results

**Fig. 1:**
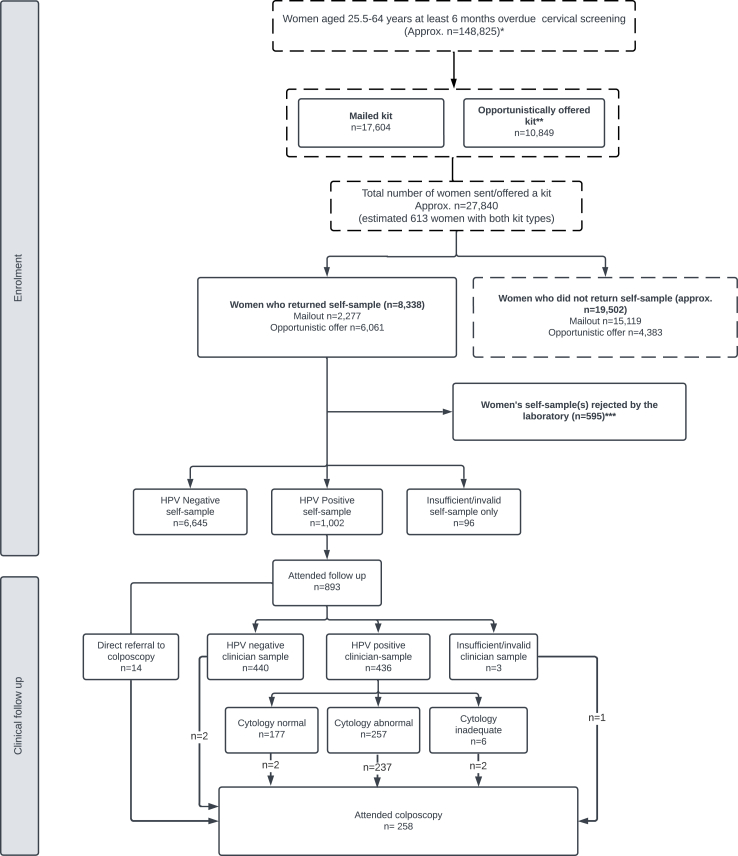
∗Estimate from aggregate GP (general practitioner) data downloaded from participating GP practices for women aged 25.5–64 y at least 6 months overdue cervical screening on 15th February 2021 (or 15th March 2021 for five GP practices missing from February 2021 data download). ∗∗Numbers are based on aggregate GP data which are approximate due to underrecording of opportunistic offers and women who may have left the GP practice during the trial. ∗∗∗Includes 154 women deemed by laboratory to be “ineligible” (146 <6 months overdue when self-sample received, 8 not on routine recall due to previous abnormal result), 350 women whose samples had insufficient identifiers or were missing the laboratory request form, 81 women who returned samples after study end, 10 women for other reasons Dashed lines indicate that numbers are approximate. Numbers may not add up as some women were offered both kit types and/or returned more than one kit type. Women returning more than one type are classified according to the first kit returned. Insufficient/invalid = unable to produce a valid result due to negative beta-globin or other sample analytical issues. Repeat kits issued to eligible women with rejected kits or an invalid/insufficient Human Papillomavirus (HPV) result. HPV self-sample result shown is for the valid HPV result if obtained. Of the 96 women with insufficient/invalid self-sample results, 21 tested HPV negative but were reported as invalid because they were received >14 days after the sample was taken 4 women whose self-samples were analysed were subsequently discovered to have no cervix and are excluded from the reporting of clinical results in the text (3 HPV negative, 1 HPV positive). Women seen in colposcopy following negative/normal clinician-taken follow up results were presumably symptomatic referrals (e.g. abnormal bleeding, cervical ectropion or polyps).

**Fig. 2:**
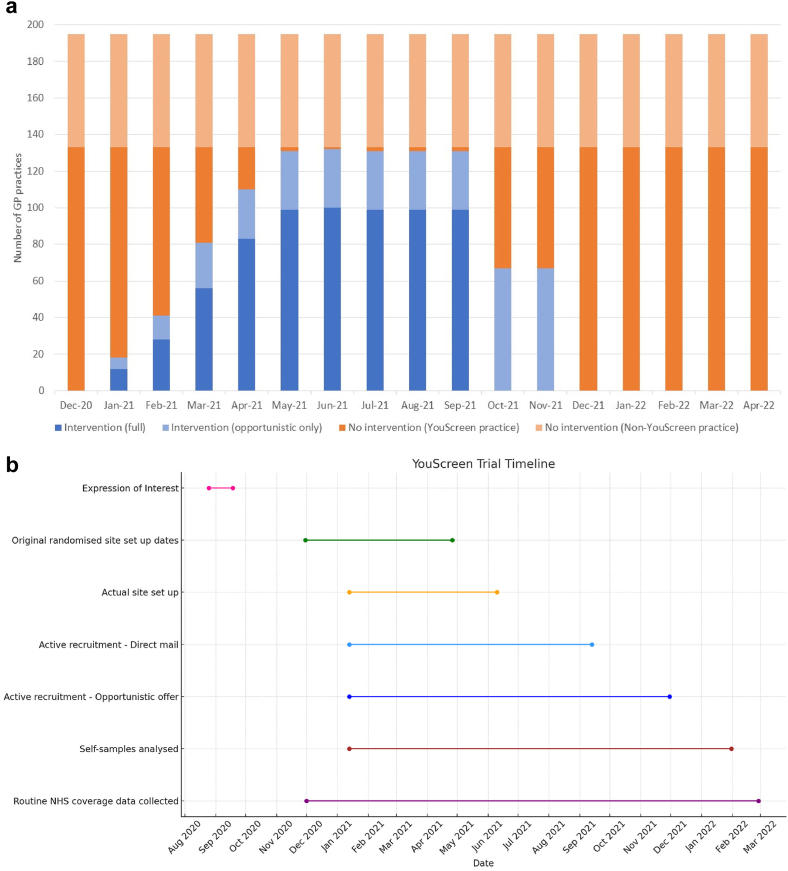
**(a) Overview of YouScreen trial ("step-wedge") intervention. (b) YouScreen trial timeline**. NHS, National Health Service.

Fig. 1 shows the CONSORT flow diagram. Fig. 2a and Figure S1 summarise GP practice participation. Fig. 2b provides an overview of key trial events and data collection timepoints. Of 195 eligible GP practices, 164 (84.1%) expressed interest in participating and 133 (68.2%) were activated for an average of 7.5 months (range 4–10 months). Most started within 60 days of the randomisation date. One practice was closed as a study site for failure to comply with the protocol and was excluded from coverage estimates. Participating practices were larger than non-participating (median numbers eligible for screening 2639 vs 1875) (Table S1). However, they had similar life-expectancy of female patients (84.6 vs 84.8 years) and median IMD 2019 deprivation scores. At study start, there were an estimated 147,965 non-attenders were registered at participating practices. An estimated 27,840 were offered kits between January 13, 2021, and November 30, 2021, and 8338 returned them. For direct-mail, 17,604 kits were sent and 2277 (12.9%) were returned. Of an estimated 10,849 women offered a kit opportunistically, 9248 (85.2%) accepted and 6061 (55.9%) returned a self-sample.

The proportion of those mailed or offered a kit who returned a self-sample within 90 days was 11.6% (95% CI 11.2–12.1) for direct-mail and 55.0% (95% CI 53.9–56.1) for opportunistic, with most returned kits received within a week (Table S2).

Half (52.0%, 4312/8285) of those who returned a self-sample were at least two years overdue screening (including never previously screened) (Table 1). Almost two-thirds were from ethnic minority groups (63.6%) or the two most deprived quintiles (60.6%), mirroring the distribution of the underlying non-attender population. The proportion of participants from ethnic minorities far exceeded that in women aged 25–64 years in the five boroughs in the 2011 Census (63.6% vs 43.6%). Table S3 shows that response rates for opportunistic offers were broadly homogenous by age, screening history and ethnicity. By contrast, for directly-mailed kits response decreased with age and time since last screen.

**Table 1:** Characteristics of women who returned a self-sample and the underlying non-attender population.

	Direct mail		Opportunistic		All		Non-attender population^d^		YouScreen Boroughs
	N	%	N	%	N	%	n	%	%
Total	2277	27.3%	6061	72.7%	8338	100%	147,965	5.6%	
**Age (years)**									
25–29	425	18.7%	1317	21.7%	1742	20.9%	36,776	24.9%	
30–39	712	31.3%	1940	32.0%	2652	31.8%	60,940	41.2%	
40–49	585	25.7%	1405	23.2%	1990	23.9%	29,003	19.6%	
50–59	411	18.1%	890	14.7%	1301	15.6%	14,866	10.0%	
60+	144	6.3%	509	8.4%	653	7.8%	6380	4.3%	
**Screening status**^a^									
Not ≥6 m overdue	11	0.5%	182	3.0%	193	2.3%			
Late (≥6–<24 m)	1383	60.8%	2397	39.9%	3780	45.6%			
Very late (≥24 m)	366	16.1%	1651	27.5%	2017	24.3%			
Never screened	514	22.6%	1781	29.6%	2295	27.7%			
*Missing*	*1*		*47*		*48*				
**Ethnic background**^b^									
White	679	39.4%	1659	35.3%	2338	36.4%	51,929	40.6%	56.4%
Asian	498	28.9%	1407	29.9%	1905	29.6%	37,433	29.3%	25.1%
Black	164	9.5%	504	10.7%	668	10.4%	11,836	9.3%	11.4%
Mixed	298	17.3%	878	18.7%	1176	18.3%	19,068	14.9%	7.1%
Other	83	4.8%	256	5.4%	339	5.3%	7507	5.9%	
*Unknown/missing*	*555*		*1357*		*1912*		*20,192*		
**IMD (quintile)**^c^									
Q1 (Most deprived)	406	17.8%	1192	19.8%	1598	19.3%	3487	19.8%	19.5%
Q2	1012	44.5%	2371	39.5%	3383	40.8%	7952	45.2%	39.2%
Q3	432	19.0%	1208	20.1%	1640	19.8%	2987	17.0%	18.1%
Q4	281	12.3%	836	13.9%	1117	13.5%	2069	11.8%	14.6%
Q5 (most affluent)	145	6.4%	403	6.7%	548	6.6%	1094	6.2%	8.6%
*Not available*	*1*		*51*		*52*		*15*		

a Screening status defined as ‘Not ≥6 m overdue’ = screened within the last 3.5 years if aged 25–49 y or 5.5 years if aged 50–64 y, Late’ ≥6–<24 m overdue, ‘Very late’ = at least 24 m overdue; ‘Never’ = not screened previously (women aged <28 y without previous screens categorised as ‘late’).

b Ethnicity data for (i) YouScreen participants is missing for 5 GP practices, proportions are calculated using those with known ethnicity; (ii) non-attender population uses aggregate GP record data downloaded from participating practices on 15.02.2021 (one month after global study start) and (iii) YouScreen boroughs uses UK Census 2011 data for females aged 25–64 years https://www.nomisweb.co.uk/census/2011/lc2101ew.

c IMD, Index of multiple deprivation English Indices of Deprivation (ID) data for London at LSOA and borough level for the (ID2019) release. https://data.london.gov.uk/dataset/indices-of-deprivation.

d Age and ethnicity use aggregate GP data downloaded from participating practices on 15.02.2021 (one month after global study start) and 15.03.2021 for 5 GP practices that were missing from data from 15.02.2021. IMD is based on all women sent a mailout kit as these women were selected in an unbiased way and are therefore, representative of the underlying non-attendee population.

Opportunistically offered kits that were accepted in-person were more likely to be returned than those accepted via remote consultation (which needed to be collected by women): 72.4% (3537/4887) vs 47.3% (900/1901), respectively.

Of the 668 women issued a repeat kit for rejected or invalid samples, 316 (47.3%) returned it, 262 (39.2%) did not and 90 (13.5%) attended for a conventional screen instead.

The funnel plot in Figure S2a shows that the proportion of directly mailed kits returned by women in different practices was broadly homogeneous across GP practices. By contrast, the success of the opportunistic offer was highly variable (Figures S2b and S2c). Figure S2b shows that most practices fall outside of the 95% limits for the ratio of opportunistic kits returned to the number of eligible women at study start. Less (but still substantial) heterogeneity is seen for the ratio of opportunistic kits returned to those offered (Figure S2c).

The weighted average “before and after” coverage in participating practices increased from 58.1% to 59.3% but decreased slightly in non-participating practices from 59.8% to 59.4% (Table 2). This corresponded to a 1.6% (95% CI 0.4–2.8) improvement in coverage associated with the intervention and an additional 6450 women “covered” in participating practices. Unweighted estimates found that coverage increased by 1.02% in participating and decreased by 0.75% in non-participating practices, corresponding to an additional 7135 women screened.

**Table 2:** Estimated increase in coverage associated with the YouScreen intervention (unweighted and intention-to-treat weighted linear regression model).

	Non-participating “control” GP practices (n = 62)				Participating “intervention” GP practices (n = 131)^a^			
	Number of women adequately screened	Number of eligible women	Unweighted coverage % (95% CI)	Weighted coverage % (95% CI)	Number of women adequately screened women	Number of eligible women	Unweighted coverage % (95% CI)	Weighted coverage % (95% CI)
December 2020	76,857	129,339	59.4 (59.2–59.7)	59.8 (57.8–61.7)	224,245	386,362	58.0 (57.9–58.2)	58.1 (56.6–59.5)
February 2022	82,652	140,868	58.7 (58.4–58.9)	59.4 (57.3–61.5)	238,100	403,116	59.1 (58.9–59.2)	59.3 (57.8–60.8)
Change in coverage			−0.75	−0.4 (−1.4–0.6)			1.02	1.2 (0.6–1.8)
Difference in change							1.8	1.6 (0.4–2.8)
Number of additional women screened							7135	6450

Note: Weighted coverage calculations are derived from the regression model therefore, differ from coverage calculated using the raw numbers.

a Two participating practices excluded (1 closed early due to excessive protocol violations, 2 merged during the trial (analysed as a single practice)).

Despite imperfect compliance with randomised start date, intention-to-treat analysis showed a highly significant increase in the monthly number of non-attenders screened of 12% (incident rate ratio (IRR) 1.12, 95% CI 1.09–1.15) (Table 3). The “per protocol” analysis which included all practices and used the actual intervention dates resulted in an increase in 22% of non-attenders screened each month in which self-sampling was offered throughout (IRR 1.22, 95% CI 1.18–1.26). See Tables S4 and S5 for additional information (including a modified ITT analysis which ignores the assigned intervention start dates in the 31 practices that were randomised but were not set up and therefore did not offer self-sampling).

**Table 3:** Estimated increase in the proportion of non-attendee women screened associated with self-sampling per practice-month.

Model 1–Intention-to-treat analysis (ITT)–186 GP practices: 124 randomised and 62 control	Practice months	Number of screens	Person-years	Average screens per practice month	Relative excess		IRR (95% CI)		Number of screens per 100 person-years
Control practices	610	8246	43,902	13.5			0.98 (0.86–1.10)		18.8
No intervention	1572	31,750	165,804	20.2	Ref		Ref		19.1
Intervention	1132	28,063	120,437	24.8	1.23		1.12 (1.09–1.15)		23.3
Peri-intervention	389	7680	38,102	19.7	0.98		0.99 (0.86–1.10)		20.2
**Model 2–Per-protocol (PP) analysis–all 193 GP practices**	**Practice months**	**Number of screens**	**Person-years**	**Average screens per practice month**	**Relative excess**	**Total excess**	**IRR (95% CI)**	**Additional non-attendees screened by the intervention**	**Number of screens per 100 person-years**
Control practices	1230	19,512	99,280	15.9			1.00 (0.91–1.10)		19.7
No intervention	1531	32,355	167,583	21.1	Ref		Ref		19.3
Full intervention	532	15,806	63,066	29.7	1.41	4563	1.22 (1.18–1.26)	2850	25.1
Direct mail only	45	753	3458	16.7	0.79	−198	1.15 (1.06–1.25)	98	21.8
Opportunistic only	179	4168	19,491	23.3	1.10	385	1.23 (1.18–1.28)	779	21.4
Peri-intervention	323	7636	35,381	23.6	1.12	810	1.06 (1.03–1.10)	432	21.6
Total						**5560**		**4159**	19.7

Model 1 excludes the 7 early adopter practices and uses randomized start dates and allocated closure dates for classifying months and calculating person-years.

Model 2 includes all practices and uses actual intervention start and end dates for classifying months and calculating person-year. Opportunistic offer “end date” was defined as the first date in the final month that at least 3 offers were made. The month in which the intervention started was classified as “peri-intervention” (unless kits were offered from the first of that month). If the assigned start date was prior to that month, the months between the assigned start and the actual start were classified as “no intervention” rather than “peri-intervention”.

Note: IRR, Incidence rate ratio. Control practices are the non-participating GP practices in the 5 YouScreen boroughs.

We also calculated the number of screens in women overdue screening per 100 person-years in control practices and by intervention in the randomised practices. There were 18.8 screens per 100 in the control practices and 19.1 in months with no intervention in the randomised practices. In the ITT analysis there were 23.3 screens per 100 when the intervention should have been offered. In the per-protocol analysis there were 25.1 screens per 100 person-years when the full intervention was offered, 19.7 in control practices and 21.4 in opportunistic only practices.

Self-samples from 7739 women were analysed and reported (excluding four ineligible women who did not have a cervix, one HPV positive, three HPV negative) (Fig. 1). HPV prevalence amongst women with a valid result was 13.1% (1001/7643), comprising mainly non-16/18 HPV types (77.1%, (772/1001)). “Invalid” results were reported for 96 women including 21 who tested HPV negative but self-samples arrived outside of the 14-day validity period.

Overall, 84.9% (95% CI 82.5–87.0) of women who tested HPV positive on a self-sample attended for follow-up within 6 months. By study end, this was 89.2% (95% CI 87.1–91.0). Compliance to follow-up was high for all age groups, ethnic backgrounds, IMD and screening history (Table S6).

Of those who underwent a clinician-taken sample for follow-up: 50.0% (440/879) tested HPV positive; 29.2% (257/879) had abnormal cytology (and were referred to colposcopy); and 20.1% (177/879) had negative cytology. For the remaining women, 49.6% (436/879) had an HPV negative result and were therefore returned to routine screening and three who tested HPV unavailable/invalid had no subsequent test recorded.

In total, 257 of the 1001 (25.7%, 95% CI 23.0–28.5) self-sample screen positives were seen in colposcopy and 169 (16.9%, 95% CI 14.7–19.3) had a biopsy or treatment. According to available histology, 80 women had confirmed CIN2+ at baseline, including two invasive cancers (FIGO stage 2b and 3b) (see Table S7). This corresponds to a CIN2+ yield of 1.0% (95% CI 0.8–1.3).

No safety issues were reported during the trial and no swab breakages. However, there was one report of a woman developing a urinary tract infection after sampling from the urethra instead of the vagina.

## Discussion

This pragmatic implementation trial has provided the first large-scale evaluation of opportunistically offered self-sampling to non-attenders within an organised cervical screening programme. The opportunistic approach elicited almost five-fold higher uptake than direct mailout, highlighting the important role and influence of primary care on screening and the efficacy of an in-person offer.^18^ Opportunistic offering also had a broader reach than direct mailed kits in terms of age and screening history. Importantly, we found the potential for self-sampling to improve coverage to be substantial. Routine roll-out would yield an estimated 7.4% (absolute) increase in coverage over a three-year screening round (1.6% (95% CI 1.9–12.7) over 7.5 months extrapolated using 1−((1–0.016)^(36/7.5)^) assuming the monthly reduction in nonattenders screened is constant; 8.3% based on the unweighted increase of 1.8%). In England this would translate to a change in coverage from 69.9%^2^ to 77.3%. Compliance to follow-up amongst self-sample screen positives was high (89.2% by trial end) across all ethnicities, deprivation and screening histories.

We found encouraging evidence that self-sampling could increase equity in cervical screening and reach underserved populations. Women from minority ethnic and deprived backgrounds are traditionally less likely to participate in cervical screening,^19^ but were well-represented amongst respondents. Compared with age-appropriate women in the 2011 Census, respondents were far more likely to be from a minority ethnic group (64% vs 44%). Feedback from GPs and participants indicated that self-sampling enabled screening in trans men, people with learning difficulties, mental illness, and a history of abuse.

A key strength is the real-world setting which provides a realistic estimate of impact. The study benefitted from a rich dataset derived from multiple real-world data sources. Furthermore, YouScreen is the first UK study to embed self-sampling within the screening programme. Covid-19 elicited unprecedented workload issues in GP primary care, rapidly changing work practices, and staff burnout.^20^ The response achieved in this challenging setting underscores the high feasibility and acceptability of the opportunistic approach in routine clinical care. Similarly, the high compliance to follow-up achieved in this context was reassuring. Collectively, these give confidence that the impact of our intervention has not been overestimated.

Further advantages relate to the pragmatic experimental design. The modified stepped-wedge design was both experimentally rigorous and pragmatic. The addition of data from practices that did not take part (“control”) enabled estimation of variations in cervical screening activity over calendar time, even at times when all randomised practices were offering the intervention. This was important because of the impact the pandemic had on health services. “Control” practices also allowed the quasi-experimental method of “difference of differences” to be used to assess the impact on coverage.

Conversely, the pandemic may have reduced study generalisability by influencing women's willingness or ability to attend for conventional screening or their decision to self-sample. The pausing of screening invitations in England between April 2020 and June 2020^21^ may have resulted in higher than usual numbers of women becoming at least 6 months overdue. Indeed, one estimate of the number of additional non-attenders screened due to the intervention (4,159) was only half of the number who returned a self-sample (7,643). However, other estimates based on coverage data (n = 6450; n = 7135) and screening data (n = 7115) suggested that most responders were true non-attenders rather than regular attenders influenced by the pandemic. Further, over half of those who returned a sample were at least 2 years overdue screening. Pandemic-related issues resulted in GP practice non-adherence to randomised starting week. Nevertheless, the majority adhered to within a few weeks of the randomly assigned target date.

Although London has greater ethnic diversity and lower coverage than the rest of the UK, self-samples were returned from women across all ethnic groups including those from White backgrounds. Additionally, the strongest interest in introducing self-sampling in England comes from those areas with low coverage.

Data on gender were not available, therefore unfortunately the trial is unable to provide quantifiable evidence on how uptake of self-sampling may differ for people who are biologically of female sex but do not identify as such.

Further limitations include the lack of formal safety outcomes reporting, and the exclusion of acceptability and cost-effectiveness data (to be published separately). Safety outcomes were not formally collected for the trial as self-sampling is considered to be low risk.

A final limitation relates to data completeness. Follow-up and histological outcome data relied on uploads from individual colposcopy units which may not have been complete. In addition, Cyres data are not available for women who move outside of the database catchment area. As such, attendance to follow-up and CIN2+/CIN3+ detection may be underestimated. GP data were limited by both under-recording of the opportunistic offer and unavailable data due to the NDOO.

Our findings confirmed previous UK self-sampling studies which reported modest (∼6–8%) uptake for directly mailed kits^22^^,^^23^ and much greater (44.8%) uptake when opportunistically offered in GP primary care.^12^ Generally, uptake associated with in-person offers is higher than that observed with other strategies.^18^ Peeters et al.^13^ found self-sampling uptake to be 77% (35/45) when kits were given to women unscreened for ≥3 years, consulting a single GP practice in Belgium. Although this is higher than the 55.9% opportunistic offer uptake in our study, Peeters et al. only randomised women to receive a kit once they had consented and the study was very small. “Opt-in” is another major strategy for offering self-sampling. Women are sent a letter inviting them to order a kit online, by post, email or telephone. Participation rates in research settings and implementation have ranged between 17% and 21%.^9^^,^^24^

Variation between and within GP practices in the opportunistic offer was also noted in our previous study.^12^ Evidence suggested this was driven by differences in the healthcare workers offering kits rather than characteristics of the women.

Few data exist on the impact of self-sampling on coverage. Coverage has likely increased from roughly 73%–77% in the Danish Capital Region where self-sampling has been offered to non-attenders since 2017.^9^ A nationwide switch to self-sampling for all women in Sweden during Covid resulted in an increase in coverage from 66% to 70% over one year.^25^

The high compliance to follow-up observed closely aligns with our previous study^12^ and with the experience of the Capital Region of Denmark (90% within 1 year).^9^ Lower compliance has been reported in other studies,^18^ including a UK trial (59%).^22^ In general, adherence to follow-up is likely to be higher in settings where cervical screening is provided free-of-charge and in primary care-based programmes. The need for follow up remains an important drawback of self-sampling and may be particularly challenging for deprived and ethnic minority groups, or where systematic barriers to screening access exist.^18^ Fortunately, high adherence was seen across all sociodemographic backgrounds in this pragmatic setting embedded in the English national screening programme.

An unexpected finding was that half of the self-sample HPV positive women were HPV negative on their clinician taken follow up sample. Possible explanations include clearance, vaginal infections, difference in false-positives between Roche cobas and Aptima, and HPV deposits rather than true infection. Further research and longitudinal data are needed to better understand the clinical significance and implications of this.

Falling cervical screening attendance has been relatively impervious to intervention,^26^ particularly among deprived and ethnic minority populations. YouScreen has provided definitive evidence that offering self-sampling to non-attenders can both improve coverage and address health inequities within a high-income organised screening programme. Increasing coverage of cervical screening by 7.4% (over a 3-year screening cycle) would be unprecedented in England. Although, this optimistically assumes a sustained uplift which may be challenging to maintain over three years.

Opportunistically offering self-sampling to non-attenders in GP primary care is acceptable, feasible and substantially more efficient than direct mailout. Further optimisation of the opportunistic offer strategy could be achieved by taking advantage of other digital communication tools or community pharmacy to increase outreach and return rates. The large variation observed between GP practices implies there is much to be learned from high performing practices to improve and streamline the opportunistic approach.

It is essential that screening programmes continue to adapt and evolve in order to achieve cervical cancer elimination.^1^ Integral to this will be providing a choice^27^ of speculum-free screening modalities such as vaginal and urine self-sampling^28^ and non-speculum clinician-sampling,^29^ to ensure optimal participation.

## Contributors

AL, PS, and JWal conceptualised and designed the study. AL, PS, JWal, ML, JG, KD, HP, ALa designed the study conduct. AL, KD, JG, and HP acquired the data. DP curated the data.

JG, KD and ML carried out project administration.

BN and JL statistically analysed the data. PS directed the statistical analysis.

AL acquired the funding and produced descriptive statistics.

JWal acquired funding for the survey sub-study. AL, PS and JWar interpreted the data. AL drafted the manuscript.

All authors read and approved the final version of the manuscript and critically revised the manuscript for important intellectual content. AL and PS act as guarantors. The corresponding author attests that all listed authors meet authorship criteria and that no others meeting the criteria have been omitted.

AL and BN accessed and verified the underlying data.

## Data sharing statement

Anonymous data generated by our research including the data used in this article will be made available, wherever legally and ethically required.

Deidentified patient level data and the full dataset with low risk of identification and the data dictionary are available on reasonable request from the corresponding author after approval by the trial independent oversight committee and the Chief Investigator.

The protocol is available at https://www.isrctn.com/ISRCTN12759467.

## Disclaimer

Dr Anita Lim currently works at the Medicines and Healthcare products Regulatory Agency (MHRA). Dr Lim carried out this research while working for King’s College London from January 2018 to January 2023. Dr Lim’s opinions are her own and do not reflect the view of the MHRA.

## Declaration of interests

All authors have completed the ICMJE uniform disclosure form at www.icmje.org/coi_disclosure.pdf and declare:

AL declares in-kind support from Copan Italia S.p.A in the form of provision of the 552C.80 FLOQSwab for the YouScreen study, received honorarium for a lecture from Roche (Dec 2021), travel and accommodation to attend an expert meeting from Copan (Dec 2022). PS declares in-kind support from Copan Italia S.p.A in the form of provision of the 552C.80 FLOQSwab for the YouScreen study, honara from Roche for advisory board, stock option for advisory board NSVtech.com, participates on NIHR and IARC DMC/Advisory board.

BN is the independent statistician on the data monitoring committees of several trials relating to cancer prevention and is on the trial steering committees of trials involving prostate cancer and diabetic foot.

ALawrence is employed by North East London Cancer Alliance as Chair of Expert Gynaecological Reference Group and is a Clinical advisor to National Screening Committee, Department of Health and Social Care. Previously employed by London Cancer as Gynaecological Pathway Director (2017–2020).

JL declares support in way of a waived registration fee for EUROGIN–International Multidisciplinary HPV Congress, as she was invited as a speaker.

## Appendix A Supplementary data

Supplementary material

YouScreen Protocol

Trial Steering Committee list

## References

[1] World Health Organization (2020). Global strategy to accelerate the elimination of cervical cancer as a public health problem. https://www.who.int/publications/i/item/9789240014107.

[2] NHS Digital (2022). Cervical screening programme, England - 2021-2022 [NS]. https://digital.nhs.uk/data-and-information/publications/statistical/cervical-screening-annual/england-2021-2022.

[3] Landy R., Pesola F., Castañón A., Sasieni P. (2016). Impact of cervical screening on cervical cancer mortality: estimation using stage-specific results from a nested case-control study. Br J Cancer.

[4] Marlow L.A.V., Chorley A.J., Haddrell J., Ferrer R., Waller J. (2017). Understanding the heterogeneity of cervical cancer screening non-participants: data from a national sample of British women. Eur J Cancer.

[5] Waller J., Bartoszek M., Marlow L., Wardle J. (2009). Barriers to cervical cancer screening attendance in England: a population-based survey. J Med Screen.

[6] Arbyn M., Smith S.B., Temin S., Sultana F., Castle P. (2018). Detecting cervical precancer and reaching underscreened women by using HPV testing on self samples: updated meta-analyses. BMJ.

[7] Serrano B., Ibáñez R., Robles C., Peremiquel-Trillas P., de Sanjosé S., Bruni L. (2022). Worldwide use of HPV self-sampling for cervical cancer screening. Prev Med.

[8] Creagh N.S., Zammit C., Brotherton J.M. (2021). Self-collection cervical screening in the renewed national cervical screening program: a qualitative study. Med J Aust.

[9] Ejegod D.M., Pedersen H., Pedersen B.T., Serizawa R., Bonde J. (2022). Operational experiences from the general implementation of HPV self-sampling to Danish screening non-attenders. Prev Med.

[10] Aitken C.A., van Agt H.M.E., Siebers A.G. (2019). Introduction of primary screening using high-risk HPV DNA detection in the Dutch cervical cancer screening programme: a population-based cohort study. BMC Med.

[11] Lim A.W., Mesher D., Sasieni P. (2014). Estimating the workload associated with symptoms-based ovarian cancer screening in primary care: an audit of electronic medical records. BMC Fam Pract.

[12] Lim A.W., Hollingworth A., Kalwij S., Curran G., Sasieni P. (2017). Offering self-sampling to cervical screening non-attenders in primary care. J Med Screen.

[13] Peeters E., Cornet K., Cammu H., Verhoeven V., Devroey D., Arbyn M. (2020). Efficacy of strategies to increase participation in cervical cancer screening: GPs offering self-sampling kits for HPV testing versus recommendations to have a pap smear taken - a randomised controlled trial. Papillomavirus Res.

[14] Office for National Statistics (2011). Census 2011 ethnic group by sex by age. https://nomisweb.co.uk/census/2011/lc2101ew.

[15] Copas A.J., Lewis J.J., Thompson J.A., Davey C., Baio G., Hargreaves J.R. (2015). Designing a stepped wedge trial: three main designs, carry-over effects and randomisation approaches. Trials.

[16] Public Health England (PHE) (2021). NHS cervical screening programme: cervical screening care pathway. https://www.gov.uk/government/publications/cervical-screening-care-pathway/cervical-screening-care-pathway#:~:text=The%20pathway%20is%20divided%20into,sample%20taking%2C%20colposcopy%20and%20histopathology).

[17] NHS Digital (2022). National data opt-out. https://digital.nhs.uk/services/national-data-opt-out.

[18] Costa S., Verberckmoes B., Castle P.E., Arbyn M. (2023). Offering HPV self-sampling kits: an updated meta-analysis of the effectiveness of strategies to increase participation in cervical cancer screening. Br J Cancer.

[19] Moser K., Patnick J., Beral V. (2009). Inequalities in reported use of breast and cervical screening in great Britain: analysis of cross sectional survey data. BMJ.

[20] GP online (2021). Basic GP workload far outstrips pre-pandemic level as practices deliver COVID-19 jabs on top. https://www.gponline.com/basic-gp-workload-far-outstrips-pre-pandemic-level-practices-deliver-covid-19-jabs-top/article/1711214.

[21] Pulse (2020). NHSE to start issuing cervical screening invitations again from this month. https://www.pulsetoday.co.uk/news/clinical-areas/cancer/nhse-to-start-issuing-cervical-screening-invitations-again-from-this-month/.

[22] Cadman L., Wilkes S., Mansour D. (2015). A randomized controlled trial in non-responders from Newcastle upon Tyne invited to return a self-sample for human papillomavirus testing versus repeat invitation for cervical screening. J Med Screen.

[23] Szarewski A., Cadman L., Mesher D. (2011). HPV self-sampling as an alternative strategy in non-attenders for cervical screening - a randomised controlled trial. Br J Cancer.

[24] Lam J.U., Rebolj M., Moller Ejegod D. (2017). Human papillomavirus self-sampling for screening nonattenders: opt-in pilot implementation with electronic communication platforms. Int J Cancer.

[25] Elfström K.M., Dillner J. (2022). Cervical cancer screening improvements with self-sampling during the COVID-19 pandemic. medRxiv.

[26] Staley H., Shiraz A., Shreeve N., Bryant A., Martin-Hirsch P.P.L., Gajjar K. (2021). Interventions targeted at women to encourage the uptake of cervical screening. Cochrane Database Syst Rev.

[27] Wedisinghe L., Sasieni P., Currie H., Baxter G. (2022). The impact of offering multiple cervical screening options to women whose screening was overdue in Dumfries and Galloway, Scotland. Prev Med Rep.

[28] Cadman L., Reuter C., Jitlal M. (2021). A randomized comparison of different vaginal self-sampling devices and urine for human papillomavirus testing-predictors 5.1. Cancer Epidemiol Biomarkers Prev.

[29] Landy R., Hollingworth T., Waller J. (2022). Non-speculum sampling approaches for cervical screening in older women: randomised controlled trial. Br J Gen Pract.

