# Supplementary material for: Opportunistic offering of self-sampling to non-attenders within the English cervical screening programme: a pragmatic, multicentre, implementation feasibility trial with randomly allocated cluster intervention start dates (YouScreen)
Source: eClinicalMedicine. 2024 Jul 16;73:102672. doi: 10.1016/j.eclinm.2024.102672 (PMC11490653; doi:10.1016/j.eclinm.2024.102672)
Supplement: YouScreen Protocol [file mmc2.pdf]

PROTOCOL

YouScreen: A pragmatic implementation feasibility clinical trial of offering HPV self-sampling to cervical screening non-attenders within the NHS cervical screening programme in England

This protocol has regard for the HRA guidance and order of content

RESEARCH REFERENCE NUMBERS

**REC Reference**	20/LO/0600
**IRAS Number**	264776
**Sponsor Reference**	123563
**ISRCTN Number**	TBC

PROTOCOL VERSION NUMBER AND DATE

**Version**	1.0
**Date**	19 March 2020

SIGNATURE PAGE

The undersigned confirm that the following protocol has been agreed and accepted and that the Chief Investigator agrees to conduct the trial in compliance with the approved protocol (Version 1.0 19th March 2020) and will adhere to the principles outlined in the GCP guidelines, the Sponsor's (and any other relevant) SOPs, and other regulatory requirements as amended.

I agree to ensure that the confidential information contained in this document will not be used for any other purpose other than the evaluation or conduct of the clinical investigation without the prior written consent of the Sponsor.

I also confirm that I will make the findings of the trial publicly available through publication or other dissemination tools without any unnecessary delay and that an honest accurate and transparent account of the trial will be given; and that any discrepancies and serious breaches of GCP from the trial as planned in this protocol will be explained.

For and on behalf of the Trial Sponsor:

Signature: .... Date: ....../....../......

Name (please print): ....

Position: ....

Chief Investigator:

Signature: .... Date: ....../....../......

Name: (please print): ....

(Optional)

Statistician:

Signature: ....

Name: (please print): ....

Position: ....

KEY TRIAL CONTACTS

**Chief Investigator**	**Dr Anita Lim**<br>King's College London<br>Faculty of Life Sciences & Medicine<br>School of Cancer & Pharmaceutical Sciences, Cancer Prevention Group<br>Innovation Hub, Guy's Cancer Centre, Guy's Hospital<br>Great Maze Pond, London SE1 9RT<br>T: 0207 848 1691<br>E: anita.lim@kcl.ac.uk
**Clinical Trial Design and Statistics and Co-Investigator**	**Professor Peter Sasieni**<br>King's College London<br>Faculty of Life Sciences & Medicine<br>School of Cancer & Pharmaceutical Sciences, Cancer Prevention Group<br>Innovation Hub, Guy's Cancer Centre, Guy's Hospital<br>Great Maze Pond, London SE1 9RT<br>T: 0207 188 4484<br>E: peter.sasieni@kcl.ac.uk
**Clinical Trials Unit**	**Cancer Research UK & King's College London Cancer Prevention Trials Unit (CPTU)**<br>King's College London<br>Faculty of Life Sciences & Medicine<br>School of Cancer & Pharmaceutical Sciences, Cancer Prevention Group<br>GH0603004 Research Oncology Seminar Room<br>Floor 3, Bermondsey Wing, Guy's Hospital,<br>Great Maze Pond,<br>London SE1 1UL<br>T: 020 7848 9702<br>E: youscreen@kcl.ac.uk
**Sponsor**	**Misha Ladva**<br>University College London<br>1st Floor Maple House, 149 Tottenham Court Road<br>London<br>WC1E 6BT<br>T: 0207 6796 469<br>E: misha.ladva@ucl.ac.uk
**Health Psychology**	**Dr Jo Waller**<br>Reader in Cancer Behavioural Science<br>School of Cancer & Pharmaceutical Sciences<br>Cancer Behavioural Science Group<br>T: 0207 848 4675<br>E: jo.waller@kcl.ac.uk
**Trial Statistician**	**Dr Francesca Pesola**<br>King's College London<br>Cancer Prevention Group<br>Guy's Hospital<br>Great Maze Pond, London SE1 9RT<br>T: 020 7848 4675<br>E: francesca.pesola@kcl.ac.uk

List of laboratories

**HPV testing**	**Central NHS laboratory**

	**Health Services Laboratories (HSL)**<br>1 Mabledon Pl,<br>Kings Cross,<br>London WC1H 9AX
**Tissue Bank**	**Guy's & St Thomas' Research Tissue Bank**<br>**King's College London**<br>Guy's Hospital<br>Great Maze Pond, London SE1 9RT
**List of central facilities**	
**Print company**	**CFH Docmail Ltd**<br>Wells Rd, Radstock BA3 3UP<br>T: 01761 416311<br>Mob: 07739 976458<br>E: Jason.Lusty@cfh.com
**Collaborator & Funder**	**North Central and East London Cancer Alliance**<br>Naser Turabi<br>Programme Director, North Central and East London Cancer Alliance<br>UCLH NHS Foundation Trust, Cancer Division, 2nd Floor, 47 Wimpole Street, London W1G 8SE<br>T: 020 3447 2782<br>E: n.turabi@nhs.net
**Collaborators**	**NHS England London**<br>Dr Josephine Ruwende<br>Consultant Public Health, Cancer Screening Lead<br>NHS England London Region/Public Health England London<br>Southside<br>105 Victoria Street<br>London, SW1E 6QT<br>T: 01138070807<br>E: josephine.ruwende@nhs.net<br><br>**NHS Digital**<br>John Willis<br>Product Manager (Cervical Screening)<br>Screening Technology Products<br>T: 01392 206796 | 07879 055 685<br>E: john.willis4@nhs.net
**Partner**	**Public Health England**<br>c/o Professor Anne Mackie, Director of Screening – PHE<br>PHE Screening Anne.Mackie@phe.gov.uk +44 (0)20 368 20893<br>Ruth Stubbs National cervical screening programme manager<br>PHE Screening<br>Ruth.Stubbs@phe.gov.uk<br>+ 44 (0) 114 201 3015<br>+ 44 (0) 7794291424
**Joint Steering Committee**	c/o UCLH NHS Foundation Trust, Cancer Division, 2nd Floor, 47 Wimpole Street, London W1G 8SE
**Study Delivery Group**	c/o Cancer Research UK & King's College London CPTU, GH0603004 Research Oncology Seminar Room, Floor 3, Bermondsey Wing, Guy's Hospital, Great Maze Pond, London SE1 1UL

CONTENTS

RESEARCH REFERENCE NUMBERS 1
SIGNATURE PAGE 2
KEY TRIAL CONTACTS 3
CONTENTS 5
LIST OF ABBREVIATIONS & GLOSSARY 8
TRIAL SUMMARY 9
FUNDING AND SUPPORT IN KIND 10
PROTOCOL CONTRIBUTORS 10
THIRD PARTIES 10
1 BACKGROUND 12
2 RATIONALE 13
3 AIMS, OBJECTIVES AND OUTCOME MEASURES/ENDPOINTS 13
3.1 Aims 13
3.2 Objectives, endpoints and measures 14
4 TRIAL DESIGN 19
4.1 Trial set up 19
4.2 Active recruitment 19
4.3 Clinical Follow up 20
4.4 Passive/Long term follow up 20
4.5 Questionnaire-based sub-study 20
5 TRIAL SETTING 20
6 PARTICIPANT ELIGIBILITY CRITERIA 21
6.1 Inclusion criteria 21
6.2 Exclusion criteria 21
7 TRIAL PROCEDURES 22
7.1 Selection of GP practices 22
7.2 Participant identification 22
7.3 Consent 27
7.4 HPV testing, reporting and recording of results 29
7.5 Clinical management & NHAIS (call/recall) pathway 29
7.6 Central Laboratory Process (HPV Testing) 32
7.7 Residual samples 33
7.8 Follow up 34

7.9 Withdrawal criteria 35
7.10 End of Trial 35
8 MEDICAL DEVICES 35
8.1 FLOQSwab 552C.80 35
8.2 Roche cobas 4800 System 35
9 STATISTICAL CONSIDERATIONS 36
9.1 Sample size considerations 36
9.2 Planned analysis 38
10 DATA MANAGEMENT 39
10.1 Data collection tools and source document identification 39
10.2 Data handling and record keeping 39
10.3 Study ID 39
10.4 Datasets 40
10.5 Confidentiality 42
10.6 Record Retention and Archiving 43
11 SAFETY REPORTING 43
11.1 Definitions of Adverse Events 43
11.2 Causality 43
11.3 Expectedness 44
11.4 Recording adverse events 44
11.5 Urgent safety measures 44
11.6 Annual Safety Reporting 44
11.7 Overview of the Safety Reporting responsibilities 44
12 MONITORING, AUDIT & INSPECTION 44
12.1 Assessment and management of risk 44
12.2 Monitoring 46
13 TRIAL COMMITTEES 46
14 ETHICAL AND REGULATORY CONSIDERATIONS 47
14.1 Health Research Authority (HRA) & Research Ethics Committee (REC) approvals 47
14.2 Confidentiality Advisory Group (CAG) approval 47
14.3 Medicines and Healthcare Regulatory Agency 48
14.4 Other approvals 49
14.5 Peer review 49

14.6 Regulatory compliance 49
14.7 Protocol compliance 49
14.8 Data protection and patient confidentiality 49
14.9 Sponsorship and Indemnity 50
14.10 Amendments 50
14.11 Access to the final trial dataset 50
15 PUBLIC AND PATIENT INVOLVEMENT 50
16 FINANCE AND FUNDING 51
17 DISSEMINATION POLICY 51
17.1 Authorship guidelines 51
18 APPENDICES 52
18.1 Appendix 1: Randomisation 52
18.2 Appendix 2: Revision history 53
19 References 54

LIST OF ABBREVIATIONS & GLOSSARY

ASR	Annual Safety Report
CCG	Clinical Commissioning Group
CI	Chief Investigator
CIN2+	Cervical Intraepithelial Neoplasia grade 2 or higher
Colposcopy	A procedure which uses a more detailed view of the cervix using a special microscope to see the changes at high magnification with good lighting
Coverage	People screened (i.e. tested and have a result documented) in the population as a proportion of the number of people eligible for that scheme
CPTU	Cancer Prevention Trials Unit
CSAS	Cervical Screening Administration Service
Cyres	Software used by cytology and colposcopy clinics to record and monitor cervical screening data. Cyres has a central server database where all cytology and histology data from NHS colposcopy clinics in London are uploaded.
DNA	Deoxyribonucleic acid
DSH	Data Safe Haven
EMIS	Egton Medical Information Systems
GCP	Good Clinical Practice
GP	General Practitioner
HCA	Healthcare Assistant
HSL	Health Services Laboratories
HIV	Human Immunodeficiency Virus
HPV	Human Papillomavirus
IG	Information Governance
ISO	International Organisation for Standards
KCL	King’s College London
LIMS	Laboratory Information Management System
LSOA	Lower Layer Super Output Area
NCELCA	North Central and East London Cancer Alliance
NCL	North Central London
NCRAS	National Cancer Registration and Analysis Service
NEL	North East London
NHAIS	National Health Application and Infrastructure Services
NHS R&D	National Health Service Research & Development
NHSE	National Health Service England
NIHR	National Institute for Health Research
NTDD	Next Test Due date
PAPM	Precaution Adoption Process Model
PHE	Public Health England
PI	Principal Investigator
PNL	Prior Notification List
RedCap	A secure web application for building and managing online surveys and databases
RAC	Research Advisory Committee
REC	Research Ethics Committee
SOP	Standard Operating Procedure
SSK	Self-Sample Kit
Test of Cure	An HPV test taken after treatment for cervical abnormalities to confirm if a woman has been successfully treated
UCL	University College London
UCLH	University College London Hospital

TRIAL SUMMARY

<table>
<tr><td>Trial Title</td><td colspan="2">A pragmatic implementation feasibility clinical trial of offering HPV self-sampling to cervical screening non-attenders within the NHS cervical screening programme in England</td></tr>
<tr><td>Short Title</td><td colspan="2">YouScreen</td></tr>
<tr><td>Trial design</td><td colspan="2">A pragmatic implementation feasibility clinical trial

Setting: GP practices in 5 CCGs in North Central London and East London (Barnet, Camden, Islington, Tower Hamlets, Newham).

Self-sampling kits will be distributed using two approaches:

(1) Direct mail-out (systematic offer)
Kits to be mailed out in monthly cycles to women who reach the 15month anniversary of their last test due date without being screened (identified using the national screening database (NHAIS)).
(2) Opportunistic Offer
Women ≥6m overdue cervical screening according to their electronic GP records to be offered kits opportunistically in GP primary care when they consult for any reason (GPs, nurses or healthcare assistants/practitioners).</td></tr>
<tr><td>Inclusion Criteria</td><td colspan="2">• Women >=25 and <=64* years old, and
• Eligible for cervical screening under the NHS CSP in England, and
• At least 6 months overdue cervical screening; and
• Registered at a participating GP practice</td></tr>
<tr><td>Exclusion Criteria</td><td colspan="2">• Women unable or unwilling to provide informed consent
• Women under the care of colposcopy within the last 36 months and/or due for a test of cure (HPV test to confirm if a woman has been successfully treated after treatment for cervical abnormalities)</td></tr>
<tr><td>Planned sample size</td><td colspan="2">We expect up to ~10,000 women will return a self-sample

Assumptions:
• ~50% (106/212) of eligible GP practices recruited
• 9 months average recruitment period at GP practices
• An estimated 31,000 participants will receive self-sampling kits:
  o 19,000 via direct mail out
  o 12,000 kits accepted opportunistically in GP primary care
• Estimated uptake: 15% for direct mail-out and 50% of those offered opportunistically</td></tr>
<tr><td>Trial Duration</td><td colspan="2">**Set-up period**: 3-6 months (staggered at each GP practice)
Active recruitment of participants: 6-12 months at each GP practice (average 9 months)
Clinical Follow up: 6 months after end of recruitment for follow up data on all women who are HPV positive on self-sample (clinical outcome assessment)
Analysis: 3 months
Total: 21 months</td></tr>
<tr><td>Objectives</td><td>Objective</td><td>Outcome measure</td></tr>
</table>

Primary	To estimate the uptake and potential increase in coverage associated with offering self-sampling	1. Uptake of kits at 30 and 90 days and at the end of the trial<br>2. Change in coverage at participating GP practices (when additionally counting a negative HPV test on a self-sample as having been screened) from the start vs the end of the trial.<br>3. Change in the proportion eligible appropriately screened
Secondary	Multiple	
Exploratory:<br>Long-term follow up<br>(Future research)	Future research: Anonymous aggregate data follow up via NHS Digital (NHAIS database, Cyres and NCRAS) to assess impact on long term cervical screening coverage, CIN2+ rates and cancer diagnoses	

FUNDING AND SUPPORT IN KIND

FUNDER(S)	FINANCIAL AND NON-FINANCIAL SUPPORT PROVIDED
North Central and East London Cancer Alliance (NCELCA)	Primary funder, Research costs
National Institute for Health Research (NIHR)	Service support costs
NHS Clinical Commissioning Groups	Excess treatment costs
NHS England London	Secondary funder, Research costs
Copan Italia Spa	Provision of the 552C.80 FLOQSwab

PROTOCOL CONTRIBUTORS

A range of stakeholders have contributed to the development of this protocol including academic and research management staff at the King's College London Cancer Prevention Group, King's CPTU team members, NCELCA, NHS digital, NHS England London, Public Health England (PHE), NHS Cervical Screening Programme (NHS CSP) laboratories. Protocol design has taken account of the view of patients and the public via several rounds of reviews.

The Sponsor is solely responsible for trial design, conduct, data analysis and interpretation, manuscript writing and dissemination of results. They will have the final decision in all matters relating to design and implementation in line with funding conditions.

THIRD PARTIES

As this is a pragmatic implementation feasibility trial embedded within the NHS CSP the third-party suppliers may be subject to change in order to meet changing needs of the trial or to adapt to the demands of the NHSCSP. Where changes occur to third-party suppliers, all changes to contractual arrangements (including data sharing agreements where appropriate) will be managed by UCL and/or KCL (for instances where UCL has delegated responsibility for contractual arrangements to KCL).

KEYWORDS: Cervical screening, cervical cancer, nonattenders, primary care

TRIAL FLOWCHART

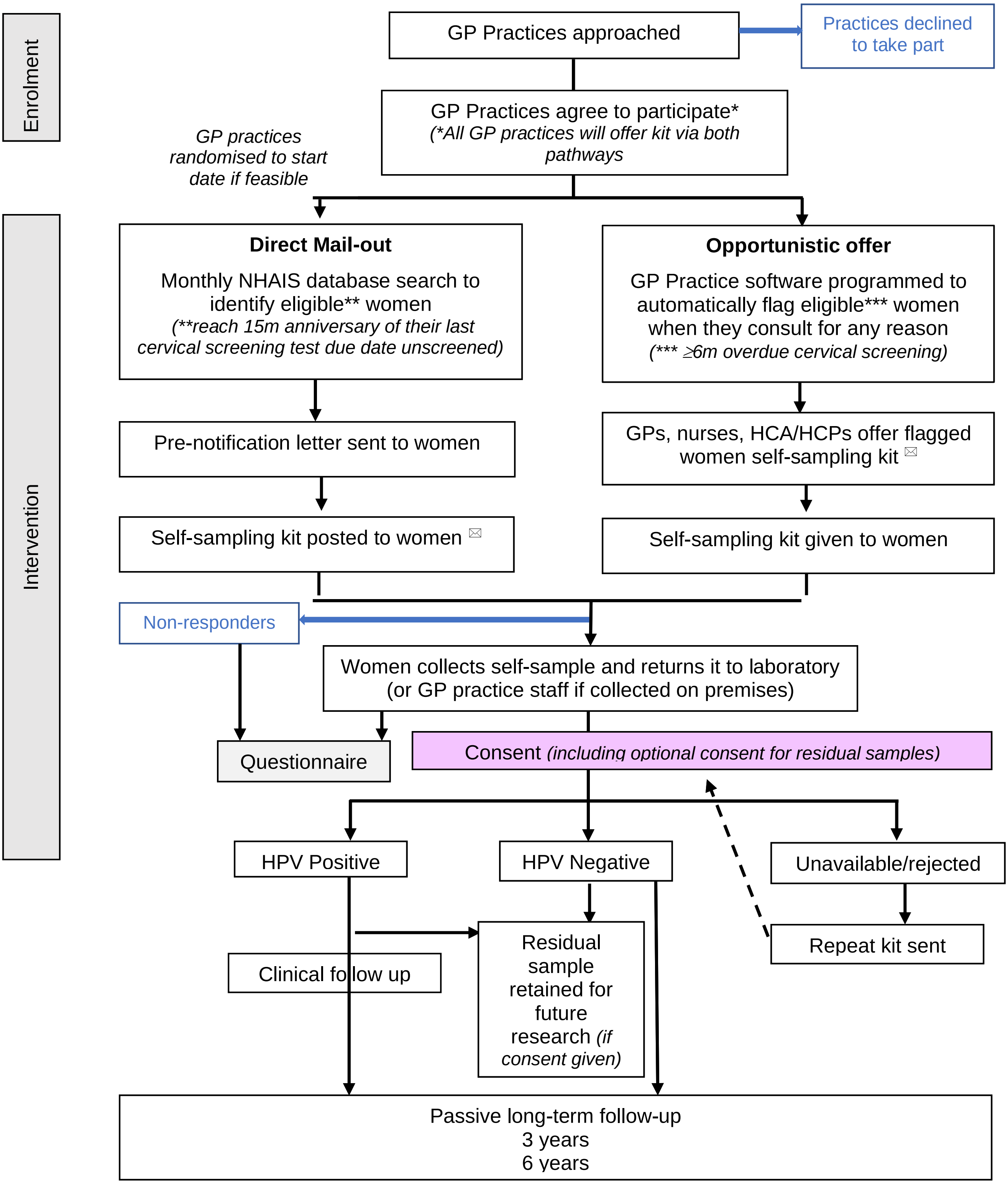

✉Opportunity to opt-out of YouScreen data sharing without consent

1 BACKGROUND

Cervical screening coverage in England has been falling and is currently at a 20 year low.(1) London consistently has the lowest cervical screening coverage nationally - 64.7% versus 71.4% across England in 2018.(1) Patches in North East (NE) and North Central (NCL) London are amongst the worst performers (age appropriate coverage in Camden CCG 54.6%, Tower Hamlets CCG 60.9% in 2017/18).(2) The North Central and East London Cancer Alliance (NCELCA) commissioned by NHS England to drive local change in the quality of cancer services such as screening, to improve cancer outcomes and patient experience. NCELCA are extremely keen to tackle the issue of falling coverage in their patches.

Currently the most promising solution to this longstanding issue is to offer self-sampling for HPV testing to women who have not attended for screening (i.e. non-attenders). Common barriers to cervical screening include fear or dislike of the pelvic examination, embarrassment, difficulty getting appointments or being too busy.(3-5) Self-sampling addresses most screening barriers enabling women to take a sample themselves, in private and at a time and place of their choosing. There is also robust evidence that offering HPV self-sampling to non-attenders can increase screening uptake, on average by 10%,(6, 7) However, uptake rates are highly variable between studies (6.4%-34%).(6, 8, 9)

In terms of clinical test performance, evidence from an international meta-analysis (including 56 accuracy studies) reported that HPV testing on self-samples are similarly accurate to clinician samples for cervical screening (i.e. for detecting high-grade cervical disease) when using an assay based on polymerase chain reaction.(6, 10, 11) A large non-inferiority trial (the IMPROVE study) that was nested within the Dutch Cervical screening programme showed that primary HPV testing on self-samples in an organised screening setting was non-inferior to clinician-taken HPV samples.(10) In addition, long term (5 year) follow data from a population-based self-sampling study in Scotland show that the risk of CIN2+ and CIN3+ in women with an HPV negative self-sample is extremely very low (0.6% and 0.2%, respectively).(12)

The self-sampling approach is highly acceptable to women and meta-analysis shows that many women prefer it over clinician sampling.(13) A consistent negative finding is that women worry about not taking a good sample.(13, 14) This concern is unfounded, as women almost always take a good quality sample (~99%),(6) but highlights the need for clear communication and reassurance when offering self-sampling.

Direct mail out of self-sampling kits achieves the best uptake, but kit attrition rates can be high.(9) An audit of electronic GP patient records and found that 60% of women at least six months overdue cervical screening attended at least once over a year, implying that a large proportion of non-attenders could be targeted opportunistically.(15) A pilot study found that 45% of women at least 6 months overdue cervical screening returned a self-sample when opportunistically offered kits in GP primary care.(16)

Uptake in UK-based studies(16-18) has been lower than similar European countries and all have been carried out outside of the national cervical screening programme; i.e. self-samples were not recorded in the national screening database (NHAIS) and did not count towards coverage or the usual GP financial incentives for cervical screening. This is likely to have contributed suboptimal uptake. None of the UK self-sampling studies have assessed impact on screening coverage.

National screening programmes in The Netherlands(19) and Australia(20) integrated self-sampling for non-attenders into their national screening programmes in 2017 and 2018, respectively. Denmark has announced that they will do so from 2020. In addition, the Dutch programme are looking to introduce self-sampling as the primary cervical screening test.(21)

In early 2019, the UK National Screening Committee called for further study of self-sampling within well organised pilots and research projects(22, 23) and there is growing interest from the national screening programmes in introducing self-sampling. Given the observed

variability in uptake it is recommended(6, 24) that local studies are carried out to establish uptake and acceptance prior to wider roll-out.

2 RATIONALE

The longstanding issues with low coverage in NE and NCL London provide an impetus to start offering self-sampling ahead of the national screening programme. In England, there is a national cervical screening programme offered to all women aged 25-64 using a call/recall system (i.e. women are invited on a regular basis at set intervals). A challenge with introducing new tests into call/recall-based screening programmes is establishing robust pathways for accurately identifying the relevant population, recording and reporting test results and setting the correct recall intervals. It is important to understand how new interventions might work in "real world" settings and to test approaches to improve them before moving to a larger scale. This will be an implementation feasibility trial of offering HPV self-sampling in North East and Central London and is a collaboration between King's College London, the North Central and East London Cancer Alliance and NHS England London.

The project will serve to test the new pathways for delivery, establish robust pathways for accurately identifying the relevant population, recording and reporting test results and setting the correct recall intervals, generate lessons to help ensure a smooth transition for a national or London-wide roll-out and provide the evidence-base for implementing self-sampling at scale. This will include all the necessary changes to call/recall within NHAIS, NHS laboratories and GP records. Self-sampling kits will be offered to non-attenders via direct mail-out (identified via NHAIS) and opportunistically in GP primary care when they consult for any reason (by GPs, practice nurses or healthcare practitioners/assistants).

Little is known about the specific barriers that this approach to screening overcomes, or about the characteristics of the non-attenders who take up the offer of completing a self-sample test. Previous research by Waller *et al*. has shown that cervical screening non-attenders are a heterogeneous group, including women who have never heard of screening, those who have never engaged with the idea of taking part, those who are undecided, those who have made a conscious decision not to attend, and those who are positively inclined towards screening but are currently overdue.(3)

It is clear that different interventions are needed to address non-participation across these groups; but little is known about which approaches might be most suitable for which women. A questionnaire-based sub-study will be carried out within the main trial. A short questionnaire will be included in the self-sampling kits to understand more about the women who take up the offer of a self-sampling test, and about their previous barriers to participation. Addressing these questions will help to inform future approaches to the implementation of HPV self-sampling and will aid our understanding of appropriate women to target with this approach to screening.

3 AIMS, OBJECTIVES AND OUTCOME MEASURES/ENDPOINTS

3.1 Aims

- To provide the evidence-base that self-sampling can improve cervical screening coverage in England and can increase detection and treatment of high grade CIN (CIN2+)
- To test a new pathway for the implementation of self-sampling for non-attenders within the NHS cervical screening programme in England

3.2 Objectives, endpoints and measures

Objectives	Endpoints	Measures
Primary objective	Primary endpoints	
To estimate the uptake and increase in coverage associated with offering self-sampling	1. Uptake of kits at 30 days, 90 days and at the end of the trial*<br><br>2. Change in coverage at participating GP practices (when additionally counting a negative HPV test on a self-sample as having been screened) from the start vs the end of the trial.<br><br>3. Change in the proportion eligible appropriately screened.	1. Uptake: number of women screened by self-sample (at 30 days and 90 days from date kit mailed out (from print company) or given out in GP practice (GP records), and total) (from NHAIS and central laboratory) as a % of all women sent kit or opportunistically offered kit (from NHAIS for 15m and from GP record data for opportunistic)<br><br>2. Coverage: Age-appropriate coverage (as determined by the number of women who have either been screened by cytology or HPV negative on a primary HPV test or self-sample in the past 3.5y if aged 25-49 or 5.5y if aged 50-64y as a % of all those eligible for cervical screening) at participating GP practices. The endpoint is the change in coverage from the (practice-specific) beginning to the (practice-specific) end of the trial (all NHAIS data).<br><br>3. Eligible women (on the first of each month): women not ceased from screening aged 25-49 who have not been screened within the last 2.9 years or aged 50-64 and not screened within the last 4.9 years.<br>Appropriately screened: number of eligible women screened within the month. Data will be collected separately for “conventional screen”, “return of mailout kit” and “return of opportunistic kit”. The proportion eligible screened (by any approach) will be recorded each month during the trial and for the four months before the trial start. The average for the before and after period will be compared.
Secondary objectives	Secondary endpoints	
To estimate the rates of compliance with follow up for women who test HPV positive on a self-sample	*Attendance at follow up (conventional cervical screening test or colposcopy) within 6	Number of women who test HPV positive on a self-sample and<br>(i) have a conventional cervical screening test recorded in NHAIS within 6m of HPV positive

	months of testing HPV positive on a self-sample	self-sample result as a % of all HPV positive on a self-sample and<br>(ii) of these, number who attend colposcopy if conventional cervical screening test is abnormal within 6 months
To estimate the CIN2+ detection rate of self-sampling	Detection rates of disease (CIN2+) amongst women who return a self-sample	Number of women with histologically confirmed CIN2+ (i.e. CIN2, CIN3 or invasive cervical cancer) in women who return a self-sample as a % of all women who return a self-sample (central laboratory data from the Cyres database)
To estimate the impact of self-sampling on colposcopy referral	Rates of referral to colposcopy associated with self-sampling	Number of colposcopy referrals as a % of all women who return a self-sample (from central laboratory Cyres)
To examine the uptake of self-sampling according to the mode of kit offer	Uptake of self-sampling kit within 6m after invitation when:<br>Sent kit directly (mail-out)<br><br>Vs<br><br>Offered kits opportunistically	Direct mail out: number of women returned self-sample as a % of total eligible sent kit directly (using data provided by NHAIS and mailing company)<br><br>Vs<br><br>Opportunistic: number of eligible women who returned a self-sample using opportunistic kit (data provided by NHAIS (sample “Source” field) as a % of total eligible women with Read codes for kits offered in GP records (GP record download)
To assess the impact of self-sampling on inequalities	Uptake of self-sampling kits according to (i) ethnic group and (ii) Lower Layer Super Output Area (LSOA) overall and by mode of kit offer	Compare distribution of (i) ethnicity (British Census categories from GP records and self-report on survey) and (ii) LSOA (from NHAIS) for women who have a self-sample result (NHAIS data linked with GP record data) overall, and by mode of kit offer (mailout vs opportunistic offer)<br>Vs<br>• women who have a standard screening test (NHAIS data linked with GP record data) over the trial period and<br>• women who are ≥6m overdue (mailout or eligible for opportunistic offer) but not screened by any method during trial (i.e. non-responders).

To estimate the cost effectiveness of this intervention	Cost per woman screened or per case of CIN2+ detected	Cost of self-sampling kit * total number of kits sent or offered (NHAIS & GP records) as a % of number of women with histologically confirmed CIN2+ in women who return a self-sample (Cyres data)
To assess the logistics of testing samples, recording results and giving women their results in a timely manner.	Turnaround time for HPV testing on self-samples	Using laboratory data:<br><br>Mean (range) number of days between:<br>• date sample taken and date sample received at laboratory<br>• date sample received and date analysed<br>• date sample received and date results released into LIMS<br>Number of samples not analysable because:<br>• Date sample taken is missing<br>• Sample returned outside of acceptable validity window<br>as a % of all samples returned
To assess the acceptability of self-sampling in cervical screening non-attenders	Uptake of self-sampling kits within 6m and compliance to follow up if self-sample positive as above*<br><br>Future screening preferences (survey sub-study)	*As above<br><br>Number of women who return a survey and endorse "self-sampling" as their preferred future screening choice as a % of all women who return a survey
To gain a better understanding of the profile of women who take up the offer of a self-sampling test, and about their previous barriers to participation (questionnaire sub-study) including:		
• previous barriers to cervical screening among women who do (and do not) return a self-sample kit (Survey sub-study)	Endorsement of questionnaire items assessing previous barriers to screening participation	Compare responses to 12 yes/no/not sure questionnaire items on previous screening barriers between women who do vs. do not return a self-sample kit.

• previous stage of screening uptake among kit completers using the Precaution Adoption Process Model (PAPM)<br>• Stage of screening uptake (unaware/unengaged/undecided/decided not to be screened/decided to have screening)		
• Distribution of women who self-identify as being at each PAPM stage women's confidence in having completed the kit correctly and their trust in the results		
• 5-point Likert responses to items on confidence and trust Describe confidence and trust in results among women who have completed a kit self-reported kit engagement among women who did not complete a kit	Self-reported opening of the kit; self-reported attempt to use the kit	Descriptive data on yes/no responses to two items assessing opening of the kit and attempt to use it.
Exploratory Objectives	Exploratory endpoints	
To examine in more detail the opportunistic offer of self-sampling in GP primary care (if data allow)	Response rates of eligible women offered kits in GP primary care<br><br>Reasons for declining kits<br><br>Kit offer and response to offer by health care professional type	The number of women who (i) accept a kit or (ii) decline a kit as a % of total number of eligible women offered kits opportunistically in GP primary care (GP record data)<br><br>Aggregated data on the main reasons for declining kits (GP records): number of women with each key reason provided as a % of all women recorded as declining kits (GP record data)<br><br>Number of kits (i) offered and (ii) accepted by health care professional type as a % of all kits offered (GP record data)
To assess any potential "nudge" effect towards the uptake of conventional screening associated with offering self-sampling	Uptake of conventional screening within 3 months of offer of self-sampling	At participating GP practices:<br><br>Number of women who attend for conventional screening (and do not return a self-sample) within 3 months of the date of 15-month mailout pre-notification letter is sent (NHAIS &

		CFH Docmail data) as a % of all women sent 15-month mailout pre-notification letter<br><br>Vs<br><br>Number of women who attend for conventional screening in months 12-14 as a % of all women who reach the 12-month anniversary of their last test due date without being screened (NHAIS data)
To ensure robust pathways are established for accurately identifying non-attenders for mail-out and opportunistic offer	Proportion of women accurately identified for (i) mail-out and (ii) opportunistic offer	Number of women who are correctly (i.e. eligible) and incorrectly (i.e. ineligible) identified in NHAIS as eligible for 15m mailout (NHAIS data) and (ii) ≥6m overdue cervical screening in GP primary care (GP and NHAIS data) as a % of all women identified for (i) 15m mailout and (ii) opportunistic offer, respectively (GP and NHAIS data)
To assess the demographic characteristics, cervical screening status and previous barriers to screening uptake of women who respond to self-sampling	Uptake of cervical screening by age bands, ethnic group, LSOA, cervical screening status (never screened, overdue, up-to-date) and reported reason for previous non-attendance	Number of women who return a self-sample in (i) 10 year age bands (NHAIS data), (ii) British Census ethnicity categories (GP record and survey data), (iii) cervical screening status (NHAIS data) and (iv) reason for previous non-attendance (survey data) as a % of all women who return a self-sample
To estimate the impact of offering self-sampling on long-term coverage (Long-term follow-up: future analysis)	Coverage at 13 months after trial recruitment ends	"Coverage" calculated at 13 months after last woman was sent/given a kit (NHAIS data) calculated separately for 3 different groups of women:<br>1) 0-14m (women overdue but who were not sent a kit),<br>2) 15m+ (women overdue who were sent a kit),<br>3) 16-26m (women overdue but not sent a kit because past 15m timepoint at trial start)
To estimate the (long term) NPV & PPV of self-sampling (Long-term follow-up: future analysis)	CIN2+ rates at 3y and 6y after trial end in women who return a self-sample by (i) HPV negative and (ii) HPV positive	Number of women with histologically confirmed CIN2+ (i.e. CIN2, CIN3 or invasive cervical cancer) in women who return a self-sample as a % of all women return a self-sample separately for (i) HPV negative and (ii) HPV positive (aggregate anonymous data from Cyres and the National Cancer Registration and Analysis Service)

4 TRIAL DESIGN

A pragmatic implementation feasibility clinical trial of HPV self-sampling. The trial is designed to both test the pathway for implementing self-sampling within the NHS cervical screening programme in England and to estimate the associated increase in uptake and coverage. We anticipate that most GP practices invited will want to take part, however, the final number of GP practices included will depend on the recruitment and response rates of participants (as observed early on in the trial-set up).

Eligible women will be identified and offered self-sampling kits via two different pathways running in parallel:

(i) **<u>Direct mail-out</u> (systematic offer)** - identified via NHAIS (national screening database) and kit posted to women's recorded home address, and

(ii) **<u>Opportunistic offer</u>** – identified via electronic GP records and women offered kits when they consult GP primary care for any reason.

All participating GP practices will offer kits via both pathways.

4.1 Trial set up

The trial is expected to be adopted onto the NIHR portfolio and will therefore have the support of the North Thames Clinical Research Network (NOCLOR) for identifying and setting up GP practices.

We anticipate that recruiting and setting up a large number of GP practices will be challenging. Therefore, we will take a pragmatic approach and have planned for a phased trial set up period over a target period of 3-6 months.

Given that the logistics required for setting up the opportunistic offer will be substantial (e.g. changes to the electronic patient record software, bespoke training to ensure optimal delivery of the intervention, kit supply) whereas direct mail-out can be easily initiated, it is likely that we will start the mailout almost immediately but stagger the opportunistic offer to align with the timing of activities required for site setup.

4.1.1 Randomisation

If feasible we will randomise the week or month that each GP practice starts the trial (see 18.1 Appendix 1: Randomisation). This is likely only be applied to the start of opportunistic offer (not the mailout). This design is like a form of stepped-wedge cluster randomisation (25) in which more clusters (GP practices) are exposed to an intervention towards the end of the trial than early on. The stepped wedge design helps control for confounding factors (via randomisation) on an intervention which may have an underlying temporal trend. The advantages of using this approach are that it will allow us to assess the impact of offering self-sampling more robustly and make any necessary alterations to the process early on with minimal impact to the majority of the trial sites.

4.1.2 Historical cohort "control" data

Historical (retrospective) data will be collected using a pre-written search of the electronic patient record system at participating GP practices for the 12 month period before the trial start. Women who are at least 6m overdue screening or who reached the 15m anniversary on a census date of 12 months before the trial start ("control" data) will be identified. Of these women, we will assess the proportion that were screened in the following 12 month period. This will be collected at or close to trial start ("entry") for each GP practice and will allow us to more robustly assess the impact of offering self-sampling.

4.2 Active recruitment

Active recruitment at each GP practice is planned for up to 12 months but may be longer or shorter depending on self-sampling uptake rates. Potentially, GP practices may recruit for longer if response rates are lower than expected or if further funds are made available to continue the intervention as part of service improvement (e.g. from NHS commissioners) and an amendment to extend the study recruitment period is approved.

In addition, if we find that the uptake of self-sampling is much lower than expected we may recruit additional GP practices. We will also do outreach at the CCG level to boost GP practice engagement and offering of opportunistic kits.

4.3 Clinical Follow up

After recruitment has ended, we will allow an additional 6 months period for all women who test HPV positive on a self-sample to have had follow-up testing and to collect follow up data (including histology).

Individual participant clinical follow-up length for self-sample screen positives during the current protocol will depend on the point at which they are enrolled and will range from 6-18 months.

4.4 Passive/Long term follow up

Long term coverage will be assessed at 13 months after trial recruitment ends.

Depending on permissions and feasibility, we also plan to collect follow up data for all women who return a self-sample at 3 years and 6 years after the end of active recruitment (last self-sample returned) to provide data that will inform the appropriate screening intervals for self-sampling in the future.

The specific data collection included in this long term follow up is further detailed in section 7.8.

4.5 Questionnaire-based sub-study

A cross-sectional, descriptive questionnaire-based sub-study will be nested within the feasibility trial. The purpose will be to understand more about the women who take up the offer of a self-sampling kit, and about their previous barriers to participation. A short questionnaire will be included in the self-sampling kits which are posted or handed out to women. The focus of the sub-study will be on women who do return a kit, but questionnaires will be mailed/handed to all women as part of the YouScreen kit and we will include a small number of questions designed to understand why some women do not return the kit – albeit anticipating a low response rate in this group. In this brief questionnaire sub-study, we will use the Precaution Adoption Process Model (PAPM) (26) as a theoretical framework and will draw on Dr Waller's previous work to explore previous barriers to participation.(27-30)

In the patient information booklet, women will be invited to complete the questionnaire and mail it to KCL by freepost. Questionnaires will be a single page of A4, folded and sealed (self-seal), with pre-paid postage and address printed on the reverse side. We may include a link for women to complete the questionnaire online instead of completing and returning by post.

The questionnaire will be pre-printed with the kit ID number to allow linkage to information about the mode of kit offer, return of self-sample, the woman's cervical screening history, HPV result and (if HPV positive) attendance for follow-up, as well as basic demographic information not collected as part of the questionnaire e.g. LSOA (Lower Layer Super Output Area).

5 TRIAL SETTING

All 212 GP practices in the Clinical Commissioning Groups (CCG) within North East (NEL) and North Central (NCL) London with the lowest cervical screening coverage will be invited to participate. These are:

- Camden (54.6% coverage 2017/18, 33 GP practices, NCL),
- Islington (62.8% coverage 2017/18, 33 GP practices, NCL)
- Barnet (63.0% coverage 2017/18, 57 GP practices, NCL)
- Tower Hamlets (60.9% coverage 2017/18, 36 GP practices, NEL)
- Newham (62.9% coverage 2017/18, 53 GP practices, NEL)

We estimate that approximately 106 (50%) GP practices will be interested in taking part. For IT reasons, only practices using Egton Medical Information Systems (EMIS) will be included. We will set up as many of these 106 practices as possible, depending on time and resources.

6 PARTICIPANT ELIGIBILITY CRITERIA

The trial population will comprise women who are registered at a participating GP practice, aged >=25 and <=64 who are at least 6 months overdue their cervical screening.

6.1 Inclusion criteria

A participant will be eligible for the trial if all of the following criteria apply:

- Women >=25 and <=64* years old, and
- Eligible for cervical screening under the NHS CSP in England, and
- At least 6 months overdue cervical screening; and
- Registered at a participating GP practice

A sub-group of eligible women will also be eligible for direct mailout of self-sampling kits (see section 7.2.1). – those who reach the 15-month anniversary of their last test due date without being screened.

*Women aged 65 will not be targeted but if included will not be considered a protocol violation. This is because although women aged 65+ are not invited to routine cervical screening they may still be offered a test if they have not been screened since age 50, or if they have not yet met the criteria to be ceased from the programme.

6.2 Exclusion criteria

A participant will not be eligible for the trial if any of the following criteria apply:

- Women unable to provide informed consent
- Women under the care of colposcopy within the last 36 months and/or due for a test of cure (HPV test to confirm if a woman has been successfully treated after treatment for cervical abnormalities)

6.2.1 Pregnancy

In addition to the eligibility criteria, women who are pregnant will be advised in the participant information booklet to not take part in the trial. There are no safety concerns with pregnant women self-collecting a vaginal swab, however the trial will advise against it for two reasons. The first is that the CE-mark for the Copan 552C.80 FLOQswab does not cover pregnant women because pregnant women were not included in Copan's validation studies. The second is that there are potential issues with ensuring adequate follow up within the trial lifetime if they test HPV positive on a self-sample (colposcopy is less inaccurate due to hormone changes and excisional treatment to the cervix for high grade cervical disease is sometimes deferred until post-partum). The NHSCSP recommend that pregnant women defer cervical screening until 12 weeks post-partum.

It is not practical or feasible to assess whether a woman is pregnant using the NHAIS database therefore some pregnant women may be sent kits in the post.

6.2.2 Women who have formally opted out of cervical screening

For the direct mailout cohort, women who are documented in NHAIS as having formally opted out of cervical screening (by returning a signed cervical screening disclaimer form) will not be sent kits.

However, women who have formally opted out of cervical screening may be offered a kit opportunistically at their GP practice at the discretion of the health professional consulted. This is because women may have opted out of cervical screening because of barriers associated with the pelvic examination and self-sampling may be acceptable to these women (as observed in our previous pilot study)(16).

6.2.3 Women who are HIV positive

Women who are documented as being HIV positive in the Cervical Screening Programme are recalled annually (as opposed to 3 or 5 yearly). These women will only be included in the opportunistic offering of self-sampling. By definition, they will never reach the 15-month anniversary of last test due date to trigger direct mail out of a kit as their Next Test Due Date (NTDD) is reset

every 12 months. HIV status will not be stored or reported to King's College London at any point during the trial.

6.2.4 Women who have opted out of NHS data usage via the National Opt-Out

The national data opt-out was introduced on 25 May 2018 enabling patients to opt out of the use of their data for research or planning purposes. Women who have opted out of their NHS data usage via the national opt-out will be excluded from the study.

- For the mailout cohort, women who are documented in NHAIS to have formally opted out via the national opt-out will be removed from the data extract before the kits are posted.

7 TRIAL PROCEDURES

7.1 Selection of GP practices

Expression of Interest (EOI) forms will be sent to all 212 GP practices in the five CCGs. We anticipate there will be strong interest in participating and estimate that approximately 50% (~106) of GP practices will be willing to take part.

We will use a pragmatic and adaptive approach to recruit and set up GP practices in the trial. If substantially more GP practices than the anticipated 106 express interest in participating, we may select practices at random, stratifying based on the number of under-screened women in each practice, the active recruitment period remaining and available study resources.

7.1.1 Early Adopter (flagship) GP practices

A small cohort of early adopter GP practices will be identified as flagship practices with whom we already have established relationships with and/or who have taken part in our previous self-sampling studies. We will aim to have one in each of the five participating CCGs. The trial will start at these practices and they will help us generate lessons for the wider roll-out of the trial; including piloting and refining content and structure of the Site Initiation Visit. If we randomise the start date at each practice (as described in section 4.1.1 Randomisation) these practice would be excluded from the randomisation.

7.1.2 GP training

Participating GP practices will receive full training as part of the Site Initiation Visit (SIV). Given the large number (~106) of GP practices to set-up a pragmatic and strategic approach will be taken. For example, SIVs may be carried out by CCG area in batches using a mixture of communal training (GP or CCG training days, Primary Care Networks) and/or delivered by webinar (~40 minute) with a short test afterwards to check for understanding, delivered via a skype call or on-site training (depending on GP practice preferences).

The training will cover all practical and clinical aspects of the trial (how to offer self-sampling kits, clinical pathway, frequently asked questions by women, logistics of kit management at the practice, results management). A self-sampling webpage aimed at health professionals will be made available through the North Central and East London Cancer Alliance website. The web page will host a short training video about the trial including FAQs.

7.2 Participant identification

7.2.1 Direct mail-out

Under a Section 251 exemption, eligible women will be identified using the national cervical screening database NHAIS (National Health Application and Infrastructure Services - owned by NHS England and PHE who are the data controllers for the cervical screening database). Accessing these data is the most accurate way of identifying eligible women and ensuring that the women invited to the trial are the appropriate population (i.e. women from a participating GP practice).

Once each GP practice has received training as detailed in section 7.1.2; the study team will issue confirmation for the GP practice to be included in the monthly mailout.

At the start of each month, the study team will then provide NHAIS with a table of participating GP practices. Following receipt of this table; a list of women (from participating GP practices) who reach the 15-month anniversary of their last test due date without being screened (by self-sample or

conventional cervical screening (HPV primary testing or cytology) will be extracted from NHAIS. This 15-month timepoint has been chosen as an approximate midpoint between the usual call/recall screening invitation and reminder letters. This is to make it obvious to women that the self-sampling offer is different from the usual screening invitation and also to avoid overwhelming women with letters about screening. Other than this, the 15-month timepoint is arbitrary, and could equally be taken at a similar timepoint (e.g. 18 months).

Figure 1 Timeline of call/recall letters and study direct mail-out of kits

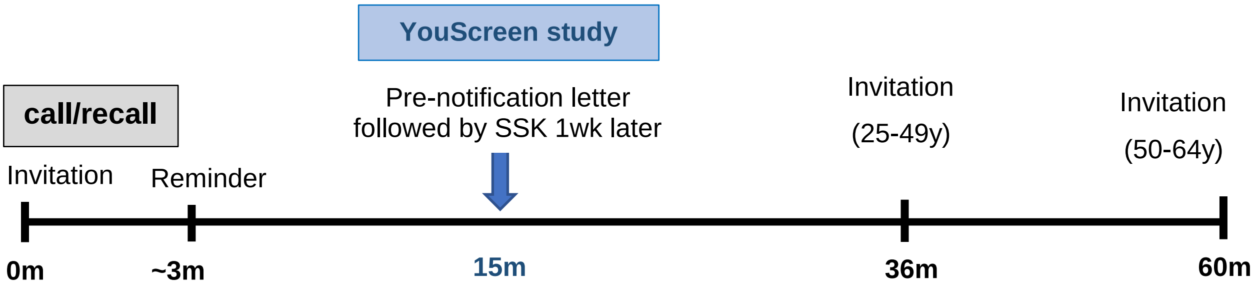

The NHAIS extract will include the women's:

- Full name
- Date of Birth
- Address including postcode
- NHS number
- GP Practice Code

These are the minimum fields required to ensure that kits can be sent to the correct women's homes identified at the lab and that the results can be accurately reported to the correct women and correct GP practice.

NHS Digital will make the list of eligible women from current participating GP Practices in NHAIS available to the Cervical Screening Administration Service (CSAS) who are responsible for sending invitations and reminders for the call/recall Cervical Screening Programme in England (see Figure 2 for further details). CSAS will then send the list to a commercial print company (contracted by UCL and/or KCL) with the appropriate level of information governance standards to securely handle NHS data (i.e. with International Organisation for Standards (ISO) accreditations and Information Governance (IG) approved on the NHS IG tool kit, their Data Security and Protection Toolkit will be compliant with NHS Digital Data Security and Protection regulations).

The print company will send a pre-notification letter to women (on behalf of the GP practices and the Cervical Screening Programme) informing them that they are overdue cervical screening and that a self-sampling kit will be posted to them. It has been hypothesised that sending a pre-notification letter enhances self-sampling uptake.(18) Within 1-2 weeks of sending the letter, the print company will send women a self-sampling kit along with an invitation letter.

GP endorsement may be included on the pre-notification and/or invitation letters as this has been shown to increase participation in colorectal screening (and would be cost neutral).(31)

Self-sampling kits in the mail-out arm will comprise:

- A CE-marked dry flocked swab (552C.80 Copan FLOQswab™) in a tube with a re-sealable lid
- Pictorial and written instructions for self-collecting the vaginal sample
- A UN3373-compliant freepost envelope (padded or polybag) or box that is pre-addressed to the laboratory

- A patient information booklet
- A specimen bag
- A lab request/consent form
- A questionnaire (questionnaire-based sub-study)

Women will be instructed to write the date sample taken on the tube label and lab request/consent form. Women will collect their self-sample at home and post it directly to the laboratory (Royal Mail freepost in line with Packaging Instruction P650 for biological substances classified as UN3373) for analysis.

Figure 2 Flow diagram for direct mail-out of self-sampling kits

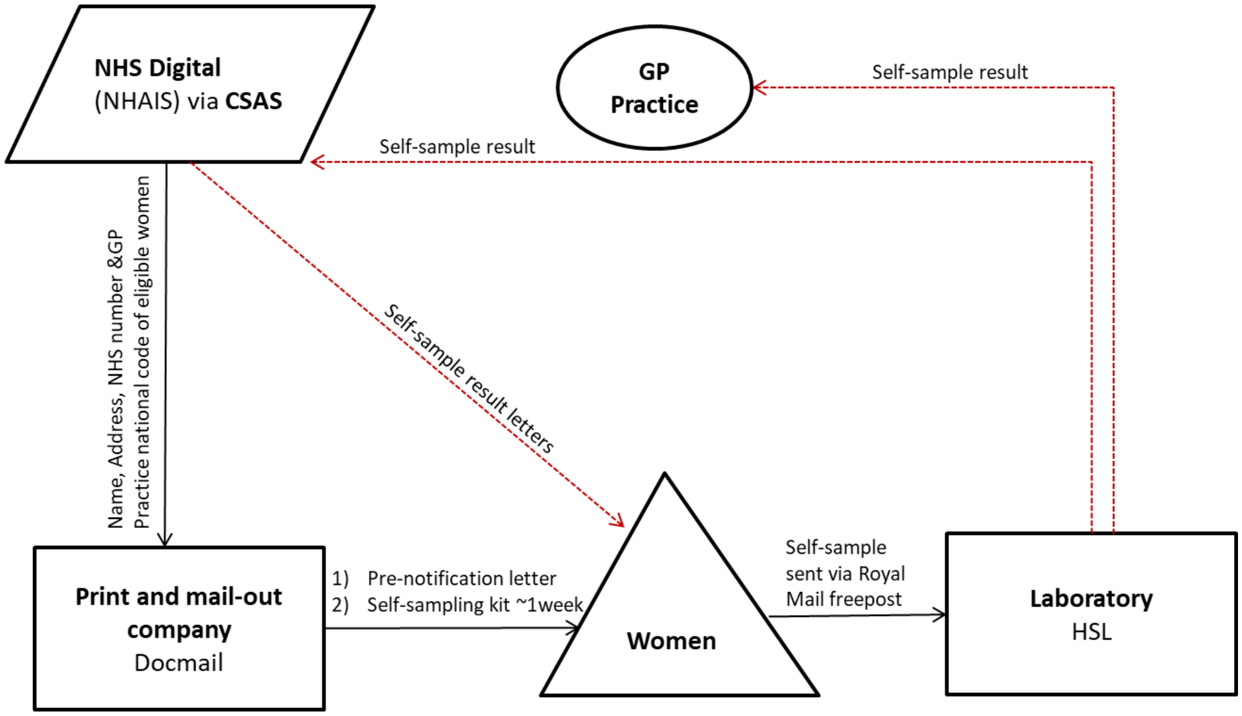

7.2.2 Opportunistic offering

Women targeted for opportunistic offering will be identified using the GP patient record software system (date of last recorded cervical screening test). Although cervical screening records in GP records are sometimes not up to date, this method of identifying the relevant cohort for opportunistic offering is currently the only viable option (GP patient records are not linked to the national screening database (NHAIS)).

An alert will be set up on the electronic patient record (EPR) software to automatically flag potentially eligible women when their record is opened. Specifically, EPR systems will be programmed to flag women aged 25.5-64 years who are at least 6 months overdue cervical screening (i.e. no cervical screening test (cytology, HPV primary or self-sample) recorded in the past 3.5 years (if aged 25-49) or 5.5 years (if aged 50-64) and no codes for hysterectomy or amputation of cervix). The alert will be switched on at the practice start date.

Doctors, nurses and healthcare assistants (HCA)/assistant practitioners (AP) will be asked to offer self-sampling kits to women who have been flagged when they consult for any reason. In practice, this will be on a case-by-case basis at the discretion of the healthcare professional consulted. The trial invitation (kit offer) will comprise a brief conversation (~2 minutes) at the end of the consultation. Healthcare professionals will be asked to confirm the women's eligibility before handing them a self-sampling kit, which includes the patient information booklet.

A study template will be set up in the GP electronic patient record system. When women are offered kits, the consulting health professional will use the template to record:
1) Whether or not the trial kit was accepted.

2) If accepted: trial kit ID number
3) If declined:
    a) Reason for declining (if logistics allow)
    b) Consent or dissent to share anonymous data from their GP records and the national cervical screening database

If the woman accepts a kit, the consulting health professional will:

- Print out a lab request/consent form with the woman's details mail-merged using the EPR system to be returned with the sample
- Write the kit ID number on the lab request/consent form
- Write three of the NHS CSP acceptable identifiers on the tube label. Preferably these will comprise the woman's name, date of birth and postcode.

To ensure optimal uptake women will be encouraged to collect the sample at the GP practice, but will also have the option of taking their kit away to collect the self-sample at home.

Depending on where the sample is collected, either the healthcare professional (clinic) or the woman (home) will write the date the sample was taken on the tube label and the lab request/consent form. Healthcare professionals will be asked to only enter a sample date on the tube label and lab request/consent form once women have collected a sample, to avoid the incorrect date appearing if women take the kit home and sample at a later date.

If possible, self-samples that are collected at the GP practice will be collected by the usual central laboratory collection services and delivered to the laboratory. If this is not possible, samples will be posted to the laboratory daily by the GP practice administrative staff using freepost, pre-addressed envelopes.

Women who collect their self-sample at home will post it directly to the laboratory (Royal Mail freepost in line with Packaging Instruction P650 for biological substances classified as UN3373) for analysis.

Opportunistically offered kits will comprise:

- A CE-marked dry flocked swab (552C.80 Copan FLOQswab™) in a tube with a re-sealable lid
- Pictorial and written instructions for self-collecting the vaginal sample
- A UN3373-compliant freepost envelope (padded or polybag) or box that is pre-addressed to the laboratory
- A patient information booklet
- A specimen bag
- A questionnaire (questionnaire-based sub-study)

Once a valid self-sample result (i.e. either Human papillomavirus self-sample test positive or Human papillomavirus self-sample test negative. Results for unavailable/Insufficient self-sample for human papillomavirus testing will be ignored) has been coded into the electronic GP records the alert will be programmed to switch off. With this trial design, potentially women may receive more than one kit offer, either opportunistically if they consult more than once during the trial period or if they are sent a kit via direct mail-out and are offered self-sampling opportunistically at their GP practice prior to an adequate self-sample result being entered. The rationale for allowing women to be offered kits opportunistically more than once is because in our previous study we found that women who declined kits accepted subsequent offers. This could happen for example if women are feeling particularly unwell at the first offer and in better health at the subsequent visit.

The laboratory will have a protocol to ensure that it rejects self-samples if one has already been tested in that screening round (i.e. in the last 3 years if aged 25-49 or the last 5 years if aged 50-64) unless the self-sample recorded was not reportable because it was unavailable or not returned in the acceptable time frame for sample validity.

Figure 3 Flow diagram for opportunistic offering of self-sampling

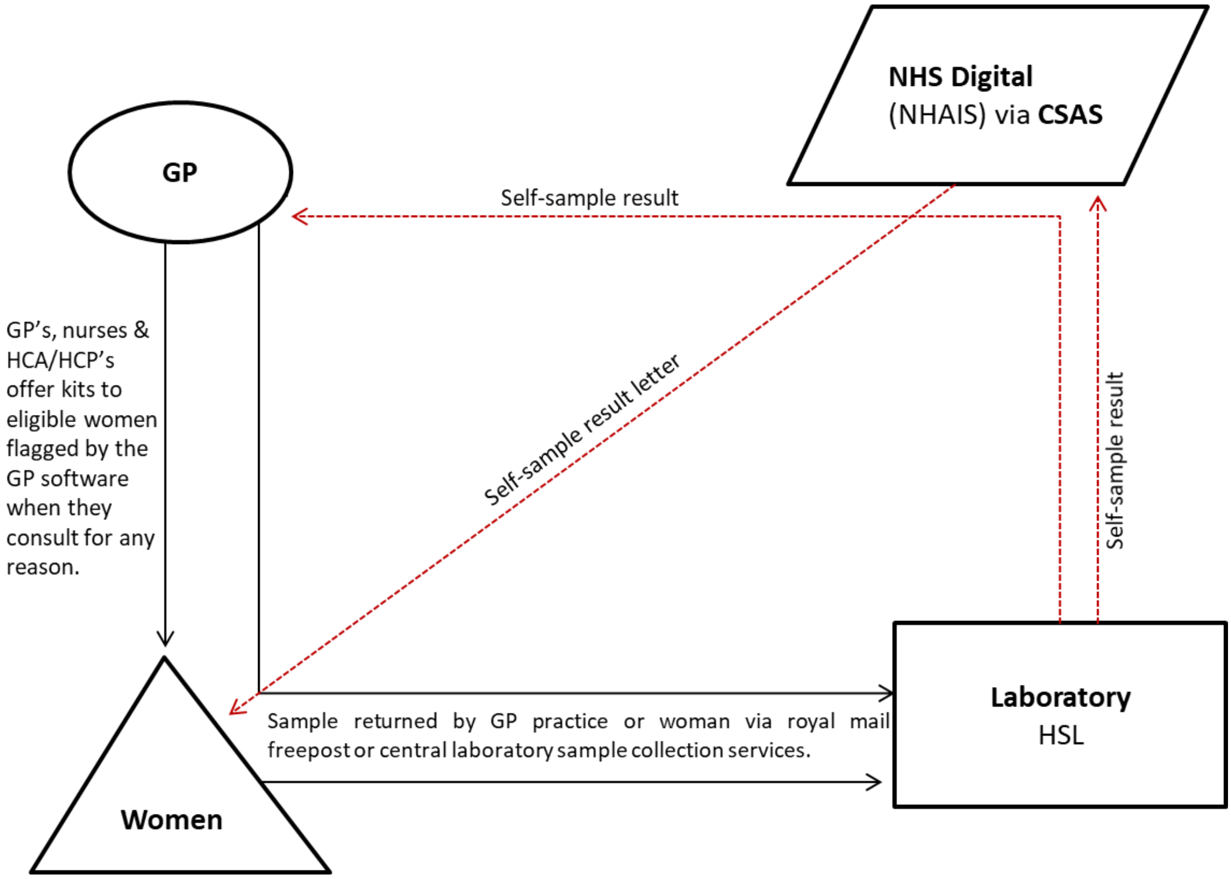

7.2.3 *Optimising self-sampling uptake*

We will use various approaches to help optimise self-sampling uptake. These include:

- A communication campaign will be developed by Claremont Communications to enhance the uptake of the kits. Claremont will deliver a targeted communications campaign to aid the recruitment and maximise uptake of self-sampling in our target population (women aged 25-64 in the boroughs of Camden, Barnet, Tower Hamlets, Islington and Newham who are at least 6 months overdue their smear test). The communications campaign will target audience's influencers, e.g. family, friends, healthcare professionals (GPs, pharmacists and practice nurses), charities, community groups, religious groups and other influencers. Claremont's campaign package will include the development of posters and leaflets. The focus of the messages will be to raise awareness and highlight that invited women will receive a kit (postal approach) or be provided one at their GP practice (opportunistic). The posters will be distributed to several stakeholders for display in key places such as GP practices, community pharmacies, sexual health clinics, community and faith organisations and offices of large employers such as local councils. Information about the trial will also be featured in local media in the respective CCGs, council and community organisation newsletters. Key organisations such as the CCGs, councils and community organisations will also be provided information to feature on their websites and social media channels. This content will also be featured on the UCLH and Small c websites (as detailed further below). The campaign package will also include a video raising awareness of the trial. The video will be featured on websites of the stakeholders that disseminate the other campaign materials as well as the UCLH and Small C websites.
- A web page with information about the trial will be hosted by the North Central and East London Cancer Alliance Small c website https://www.smallc.org.uk/. The Small c website has the "Recite me" functionality which allows the website to be fully accessible. Visitors to the site can customise the site the way they need it to work for them. The website has a translation tool for over 100 languages and text to speech functionality for 30 languages.
- The small C website will also include an animated video for women with instructions on how to take a sample.

- To educate GPs, a training video about the trial will be made available on the North Central and East London Cancer Alliance website, shared directly with practices and if possible, linked to from the YouScreen EMIS template.
- A self-sampling telephone helpline will be run by Jo's cervical cancer trust https://www.jostrust.org.uk/ for women offered kits to address any questions they have on the trial or how to take the self-sample. The helpline will provide clear, reliable and trustworthy information around self-sampling, HPV, HPV testing, cervical screening, symptoms, colposcopy, treatment for cell changes and cervical cancer.
- If uptake is lower than expected, we may send a reminder postcard or letter to women who have received kits (via either mode of kit offer) but not returned kits within 4 weeks.(32) A substantial amendment will be submitted for this.

7.3 Consent

The YouScreen patient information booklet, pre-notification letter and invitation letters will be translated into the top 3-5 most commonly requested languages in North Central & East London (Turkish, Bengali, Polish, Arabic, Somali) and made available on the small C website. As mentioned previously, the Small c website has the "recite me" translation tool for over 100 languages and text to speech functionality for 30 languages.

As this is a pragmatic implementation feasibility trial that will closely mimic the NHS CSP screening pathway in order to ensure optimal uptake in a hard to reach population, this study will not include a signed consent form.

7.3.1 Consent at GP practice level

Firstly, consent will occur at the GP practice level. Each GP practice will provide consent confirmation (via the Organisational Information Document) for their practice to take part and for eligible women to be sent kits for the trial. GP practices will consent to aggregate anonymised patient data being collated from their Electronic Patient Record systems (EMIS web) for monthly study reporting, and de-personalised line-level data on all eligible women including women who do not return a self-sample). We will seek CAG approval for this, particularly as this data will be downloaded centrally at the CCG level.

7.3.2 Consent at individual women level

This trial will be conducted as closely as possible to the likely pathway self-sampling at the national level. The patient information booklet (including information on HPV) will be provided to all women who receive self-sampling kits. As only women who wish to participate will return a self-sample (i.e. opt-in), consent is implicit.

The lab request/consent form and invitation letter will clearly state that if the women choose to complete the self-sample and send the kit to the laboratory that they are consenting to participate in the trial. The patient information booklet will specify the relevant details about the laboratory testing, retention of residual samples for future research, access to their data, and passive follow up data including line individual level linkage to previous and future cervical screening outcomes for women who consent to residual sample retention. It will also be clearly stated that this test will be completed instead of the cervical screening test taken by a doctor or nurse. The women will be informed that if they prefer to book a cervical screening appointment with a doctor or nurse that they are free to do so.

The lab request/consent form will comprise of an initials box to allow participants to opt in for their residual samples to be stored with their personal details removed for future ethically approved research.

7.3.3 Opportunistic offering of self-sampling

GP primary care is a busy and time-pressured environment with an average GP consultation length of just 9 minutes.(33) In a previous study of opportunistic offering of self-sampling in GP primary care the consent procedure consisted of a brief (2 min) study explanation and verbal consent. This simplified approach was suggested by the reviewing REC committee (Brighton and Sussex NRES Committee 13/LO/1441 and 17/LO/1655) and worked well for the study with no issues raised. Therefore, we intend to use the same verbal consent procedure for this trial. Consent to accept a

self-sampling kit will be recorded in the women's GP medical record, regardless of whether or not they return a self-sample. The lab request/consent form will not be signed.

7.3.4 Women sent kits via direct mail-out

Consent will be implicit by return of a self-sample.

Self-Sample Kits (SSKs) will come with a lab request/consent form which will contain the women's details (to identify the women) and statements detailing what the trial involves and what they are agreeing to by returning a self-sample (e.g. regarding permission for the trial team to access to medical record data). The form will state clearly what the women are consenting to by returning their sample. The lab request/consent form will not be signed.

7.3.5 Opt-out procedures for women who do not consent to the trial

YouScreen plans to collect anonymous line-level and aggregate data from NHAIS (the national screening database) and GP medical records (via the CCG) for women who do not consent to the trial. A Confidentiality Advisory Group (CAG) approval will be obtained for this activity (see 14.2 Confidentiality Advisory Group (CAG) approval).

We will provide any woman, who does not consent to the trial by returning the sample, and does not wish for her data to be used for the purposes of the trial, the opportunity to opt out of line level data collection.

The opportunities for opt-outing of data collection will include:

- Mailout offer: The invitation letter and patient information booklet sent to women who are identified as eligible for the mailout of kit will contain text advising women of the process for opting out of data collection.
- Fair processing notices in GP practices – these will be put up in all participating GP practices and will advise women to visit or call their GP Practice if they wish to opt out.
- Online - The Cancer Prevention Group webpage on the King's College website https://www.kcl.ac.uk/lsm/research/divisions/cancer/research/groups/canprev/cancer-prevention-group and the trial webpage on the Small c website https://www.smallc.org.uk/ will contain details on data processing and sharing for YouScreen and will advise women to visit or call their GP Practice if they wish to opt out.

As opting out will require exclusion of women's data from two separate data sources; GP records and the national screening database (NHAIS), two steps will be executed by the GP Practice to ensure that the request for the opt-out is upheld. CSAS and NHS Digital will facilitate this process. KCL will not receive any patient identifiable data.

- The GP Practice staff will ask the woman for her details in order to code the woman's dissent into the electronic patient record.
- The GP Practice will then provide details of every woman who has opted out (full name, date of birth, NHS number and GP practice) to a trial specific email address managed by CSAS.
- CSAS will then take responsibility of providing a regular list to NHS Digital (via a secure support ticket system) of the women who have opted out.
- NHS Digital will verify the women's identify and then add her NHS Number to an 'exclusion list'.

The coding of the dissent into the electronic patient record will allow YouScreen study EMIS searches to be programmed to exclude data with this code in trial reporting datasets (apart from aggregate reports which do not require permission).

The exclusion list managed by NHS Digital will be applied to all NHAIS machines to ensure that the NHS number of any woman on this list is removed from the YouScreen NHAIS searches to identify women at the 15m anniversary for mailout and from the trial reporting datasets (apart from aggregate reports).

GP Practices will be provided with instructions for managing the opt-out process for the study at the Site Initiation Visit.

7.4 HPV testing, reporting and recording of results

Self-samples will be analysed for the presence of high-risk HPV DNA. Full details of the testing procedures are provided in section 7.6.

As this trial aims to test the new IT pathways for self-sampling, we plan to integrate self-sampling ordering, processing of samples, results recording and reporting as much as possible into the existing systems for cervical screening programme. Self-samples will be booked into the same laboratory information management system (LIMS) that the central laboratory service use for processing cervical screening under the NHSCSP. When cervical screening tests are taken at GP practices with electronic lab links set up, the sample is automatically booked into the central laboratory (LIMS), meaning that the laboratory already has the woman's personal and test details in their system before it arrives for analysis. No pre-booking of samples will be possible for kits directly mailed out because the screening programme and GP practice databases are not linked.

"Source of sample" is a compulsory field that needs to be inputted for all cervical screening tests under the Programme. To allow us to calculate uptake according to mode of kit offer, the laboratory will enter:

1 - "GP" for self-samples returned using opportunistically offered kits, and
6 - "Other" for self-samples returned via direct mail-out kits.

A specific range of kit ID numbers and potentially different coloured sample tube labels will be set up to assist the laboratory with distinguishing between the two kit types returned.

Results will be reported using the usual routes used by the NHSCSP:

- For sending results from the laboratory to GP practice: electronic lab links will be used (including automatic coding into EMIS Web) if available, otherwise results letters will be posted to the GP practice.
- For sending results from the laboratory to NHAIS: The central laboratory LIMS database and middleware (Keystone) software will automatically pick up self-sample test results and send the relevant list of results to NHAIS via Open-Exeter. Patient data are verified via Open-Exeter ensuring that the correct result goes to the correct woman. Self-sample results that are verified by Open-Exeter will then be dropped into the NHAIS database.
- For sending results to women: NHAIS will send the relevant results letter to the women (programmed into NHAIS for the trial).

The laboratory will aim to report HPV results (i.e. authorised report released to NHAIS) within 5 days of receiving a self-sample. Once an authorised report has been successfully released into NHAIS, the relevant results letter will be triggered for release within the NHAIS system (further details below in the following section). Women should receive their results letter within two weeks of returning a self-sample (as per current NHSCSP guidelines).

7.5 Clinical management & NHAIS (call/recall) pathway

NHS Digital will implement the changes required in NHAIS to support self-sampling. A specification outlining the changes required in the system to support the self-sampling pathway has been jointly drafted with input from NHS Digital, the trial team, clinical experts (consultant gynaecologist and GPs on the Joint Steering Group), PHE, Research Advisory Committee (RAC) and HSL laboratory. This includes appropriate failsafe measures for cervical screening self-sampling. 4**Error! Reference source not found.** summarises the clinical management for women who return a self-sample in the trial and the associated self-sampling pathway in the national cervical screening database NHAIS (call/recall). Self-sampling will be recorded in NHAIS and will elicit the relevant changes in the call/recall pathway in NHAIS. This will allow us to accurately calculate the impact of self-sampling on coverage and to test the IT pathways. Proposed NHAIS codes for self-sampling are in Table 1.

7.5.1 HPV Negative

Women who test HPV negative on a self-sample will not require any further action or tests. They will have their NTDD reset within the national cervical screening (call/recall) programme according to age (3 years if aged 25-49y, 5y if aged 50-59, or if aged 60 or above at the time of testing, they will be ceased from the cervical screening programme (in keeping with the usual auto-ceasing rules).

7.5.2 HPV Positive

Women who test HPV positive on a self-sample will be advised in their results letter to have a clinician sample – a conventional cervical screening test (HPV primary test triaged with reflex cytology if HPV positive) taken by a doctor or nurse at their GP practice. These women who subsequently undergo a conventional cervical screening test will be managed according to their HPV primary screening results as per routine clinical care under the NHS CSP.

The call/recall status for women who test HPV positive on a self-sample will be updated in NHAIS to early recall in 3 months, and her NTDD will be set to 3 months from the date the self-sample was taken (as recorded on the sample tube and lab request/consent form). As cells from the cervix are not exfoliated for self-sample collection, the follow up test (conventional cervical screening test) can be taken immediately (i.e. does not require the stipulated 3 month period in between conventional cervical screening tests to allow cells to regenerate). Therefore, NHAIS will be programmed to ensure it will accept any conventional cervical screening test immediately following an HPV positive on a self-sample result.

If a follow up test (conventional cervical screening test) is not received in NHAIS within 10 weeks of the reset NTD (3m), the woman will be added to her GP practices' Prior Notification List (PNL). The PNL is a list of women at a GP practice who will be invited to be screened within the next 10 weeks. The purpose of the PNL is to allow GPs to remove women from the list who do not need to be invited for screening (e.g. recent hysterectomy or pregnant). We have requested that NHAIS add women who test HPV positive on a self-sample into the PNL so that they will receive two "reminder" letters to attend for a follow-up (clinician) test.

Self-sampling studies have found that most (~82%) women who test HPV positive attend for follow-up (cytology or colposcopy).(17, 18, 34-36) In our previous study of opportunistically offered SSKs in London, 85% of women who tested HPV positive on a self-sample attended for follow-up investigations.(16) We will collect details of follow up cervical screening tests, colposcopy referrals and histological outcomes for self-sample screen positive women from NHAIS and the central laboratory Cyres colposcopy database (with the women's permission – see section 7.3.2 Consent at individual women level).

As an additional safety-net, when the trial reaches the end of recruitment, all self-sample screen positive women who have not attended for a follow up test will be highlighted to their GP practices who will make one further attempt to contact the women to come for a follow up test. This will be either by telephone call or text message as per GP Practice local procedures. Relevant women will be identified by the participating GP practices (designated trial administrator) using a pre-written search of the GP records or by the laboratory.

7.5.3 Unavailable due to insufficient DNA

Women who return a sample that has insufficient DNA (i.e. human beta globin not detected) to produce a viable result will be asked to repeat the self-sample in their results letter sent by CSAS. This will trigger a repeat kit to be made available to them within approximately 1 week of their results letter being sent. Based on a previous study we expect this to be rare (2%).(16)

If the repeat self-sample is also unavailable due to insufficient DNA, women will be advised (in their results letter sent by CSAS) to attend their GP practice for a conventional cervical screening test (collected by a nurse or doctor). This approach has been chosen because women who return a repeat kit indicates that they are highly motivated to be screened. There are no clinical safety concerns as two consecutive unavailable due to insufficient DNA results on a self-sample indicates poor sample-taking, not higher risk of having cervical abnormalities.

Unavailable results due to insufficient DNA will not elicit an action in NHAIS and their NTDD will remain unchanged.

As with women testing HPV positive, women who have an unavailable test result (due to insufficient DNA) will be safety-netted at their GP practice. A pre-written search of EMIS web (electronic patient record software) will identify all women with an unavailable self-sample result who either do not return a repeat (2nd sample) or who test unavailable on two consecutive samples. GP practices will be asked to contact these women (by letter, telephone call or text message) to invite them to come for a conventional screening test.

7.5.4 Invalid samples

Invalid samples arriving at the laboratory will not be analysed. The sample details will be recorded on the central laboratory LIMS database but will not be reported back to the NHAIS database.

Samples will be considered to be “invalid” for analysis if they:

1. arrive >14 days after the date the sample has been taken (as recorded on the sample tube and lab request/consent form; or
2. do not meet the minimum required information on the sample tube and/or lab request/consent form to meet NHSCSP requirements for valid samples or
3. are returned by women who are ineligible.

As invalid samples will not be analysed or reported to NHAIS, a letter cannot be sent by CSAS. Therefore, the print company (CFH Docmail) will send a letter to women who return an invalid sample, informing them of this, and advising them that they will either receive a repeat kit or should book for a clinician-taken sample at their GP practice (as appropriate). The central laboratory will securely send (via secure file transfer protocol (SFTP)) the necessary details to issue these letters and repeat kits to CFH Docmail (NHS number, full name, date of birth, postal address, GP practice code, study ID (encrypted NHS number)). Results will be sent to the GP practices in the usual way (electronic lab links). The trial team at King’s will receive the list of encrypted NHS numbers with date of invalid result in order to ensure sufficient oversight for this process.

Samples returned by women who are ineligible (i.e. women who are not >=6 months overdue cervical screening) may arise if they receive a kit and are screened by conventional screening or self-sample or if they have received a kit in error. The central laboratory will contact the women’s GP practice to inform them that the sample is invalid and will therefore not be analysed or reported to NHAIS. The GP practice will in turn, contact the woman to inform her that her sample was not analysed because she is not eligible.

Detailed instructions for the handling of invalid samples by the laboratory will be included in the YouScreen Laboratory Manual, in accordance with current NHSCSP guidelines.

Table 1 Proposed codes for recording self-samples in NHAIS

Self-Sample HPV result	HPV primary screening flag	Infection Code	Result Code	Action Code	Call/recall Action in NHAIS
HPV Negative	S	0	X	A	Reset Next Test Due Date (NTDD) using the standard call/recall protocol.
HPV Positive	S	9	X	R (3)	Reset NTDD with an early recall of 3m (will restart the call/recall cycle and automatically add them to the PNL list that is sent to GPs. NTDD would be reset again if the women had standard screening test
HPV unavailable (insufficient DNA)	S	U	X	H	NTDD unchanged.

7.5.5 Pregnant women

Although pregnant women will be advised against taking part in the trial it is highly likely that several will return a self-sample. A kit may be sent in the mailout offer to a woman who is pregnant because pregnancy is not recorded on the NHAIS database.

This also may be because women may not realise they are pregnant at the time of sampling or because they do not see the text advising against it in the participant information booklet or instructions..

There are no safety issues with a pregnant woman using a vaginal swab to self-collect, even if she touches her cervix or if the swab breaks (the broken end would be at the vagina entrance, not near

the cervix). In addition, cervical screening is not contraindicated in pregnancy. However, the NHSCSP recommends pregnant women to defer their cervical screening until 12 weeks post-partum.

Therefore, if a pregnant woman returns a self-sample in the trial, her data will be included. Women with HPV negative self-sample results will be treated in the same way as set out above. HPV positive women on a self-sample should be followed up with a clinician-taken sample for cervical screening. If the GP/nurse at the practice feel unable to do this, then they should refer the woman to a cytology clinic within the local colposcopy service. Experienced clinical staff within colposcopy are more used to assessing a pregnant cervix.

Figure 4 Clinical management for self-sampling

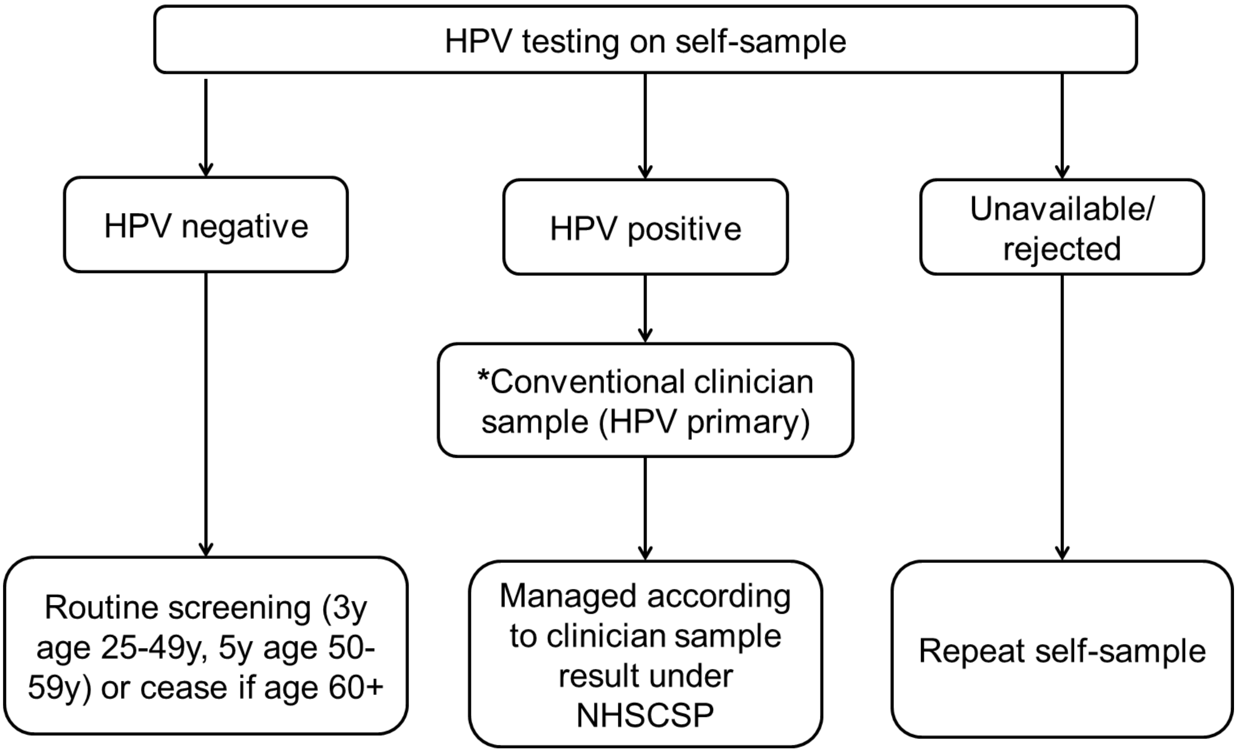

7.6 Central Laboratory Process (HPV Testing)

All valid self-samples from the women will be analysed for presence of high-risk HPV by the central laboratory in London contracted to deliver all testing for cervical screening in London as part of the NHS cervical screening programme. This is currently the Health Services Laboratories (HSL) at The Halo Building, 1 Mabledon Place, London, WC1H 9AX, however may change if there are changes within the NHSCSP. HSL will perform activities according to the standards set out in the cervical screening programme guidelines, which will be further defined in a YouScreen Laboratory Manual and HSL standard operating procedures developed for the purpose of the YouScreen trial. HSL, as the contracted London laboratory for the NHSCSP have existing access to NHAIS data.

7.6.1 Sample collection and transfer

Women who accept a self-sampling kit in their GP practice (opportunistic) will have the option of collecting their sample in the clinic bathroom or at home. Samples collected at the GP practice will be picked up daily via the usual NHS laboratory sample collection services for delivery to the central laboratory. If this is not feasible, samples will be posted daily to the central laboratory by the GP practice staff using pre-addressed freepost envelopes provided with the self-sampling kit.

Women who are sent kits via direct mail-out will collect their sample at home. All samples collected by women at home will be posted dry under ambient conditions to the central laboratory for analysis (using a freepost, pre-addressed UN3373 compliant box or envelope that will be provided with the kit). Women will be advised to post the sample within 5 days of taking it. Samples that are returned more than 14 days after sample collection will be rejected by the laboratory.

All self-sampling kits will contain freepost envelopes pre-addressed to the central laboratory at Health Services Laboratories, The Halo Building, 1 Mabledon Place, London, WC1H 9AX.

7.6.2 Sample labelling

7.6.2.1 Opportunistically offered kits

Sample tubes will be pre-labelled with a unique kit number. The GP/nurse/HCA offering the kit will write 3 identifiers (e.g. full name (first and last name counts as two identifiers, date of birth (DOB)) on the label. Women will be instructed to write the date the sample was taken on the sample tube and on the lab request/consent form. The date the sample was taken should only be entered after the sample has been taken to avoid inaccurate sample dates being received (the date sample taken will inform the NTDD in call/recall, and acceptable sample validity for analysis).

7.6.2.2 Direct mail-out

Sample tubes will be pre-labelled with a unique kit number, the woman's full name, NHS number, Date of Birth and GP National Code. If pre-labelled tubes are not possible, a peel and stick label with these details pre-populated will be included in the kit for the woman to attach to the tube. Women will be instructed to write the date the sample was taken on the sample tube and on the lab request/consent form.

7.6.3 Laboratory procedures

7.6.3.1 Sample Receipt

The central laboratory will be responsible for performing sample, documentation and eligibility checks of all self-samples received at the laboratory in accordance to cervical screening programme guidelines and the YouScreen Laboratory Manual to determine whether a sample is valid or invalid for HPV testing.

The central laboratory will also be responsible for maintaining electronic records of whether participants have consented to residual samples storage.

7.6.3.2 HPV Testing and Result Reporting

Valid samples will be tested for HPV using the Roche cobas® 4800 platform detailed further in Section Roche cobas 4800 System8.2. The cobas® 4800 Human Papillomavirus (HPV) Test is a qualitative in vitro test for the detection of Human Papillomavirus in patient specimens. The test utilises amplification of target DNA by the Polymerase Chain Reaction (PCR) and nucleic acid hybridisation for the detection of 14 high-risk (HR) HPV types in a single analysis. The test specifically identifies (types) HPV16 and HPV18 while concurrently detecting the rest of the high-risk types (31, 33, 35, 39, 45, 51, 52, 56, 58, 59, 66 and 68) at clinically relevant infection levels. The cobas® assay simultaneously tests for human beta-globin as an internal control of sufficient specimen cellularity. In addition, a positive and negative control specimen will be included in each run.

HPV testing will be performed with the liquid-based cytology (LBC) ThinPrep platform (PreservCyt) by the HSL laboratory. Samples will be prepared and processed following the Roche company guidelines.

The laboratory will record presence of human beta-globin, HPV result including HPV type (16/18 and other), although only HPV results without type will be reported to GPs and to women.

Results will be recorded in the usual NHS laboratory electronic LIMS database. Results will be reported from the laboratory to the GP practices using electronic links and automatically coded into EMIS Web if possible. Where electronic lab links are not available results will be sent via letter to the GP Laboratory Information System (LIMS).

The laboratory will stop analysing self-samples one month after the last kit has been sent out (including repeat kits for unavailable results). Women will be informed of this when they receive their kit.

7.7 Residual samples

All women will be asked to opt in by completing an initials box on their lab request/consent form to allow their residual samples to be stored with their personal details removed for future ethically

approved research. Samples will be discarded by the central laboratory according to local procedures after HPV result reporting unless the initials box on the lab request/consent form is completed to indicate their consent.

Residual samples will comprise of human DNA in the leftover elute from HPV testing on self-samples. After HPV results have been reported, residual samples belonging to participants that have opted in for residual sample storage, will be de-personalised by the central laboratory (personal identifiers removed, leaving only barcode or unique kit ID) and stored refrigerated (2-8 °C) pending shipment to the Guy's and St Thomas' Research Biobank (HTA licence number 12121) for storage pending future ethically approved research. The de-personalised residual samples will be sent by the central laboratory under the conditions defined in the material transfer agreement to the biobank facility.

At the end of the trial, residual samples will remain in storage under the Guy's and St Thomas' institutional HTA license, pending use for possible future ethically approved research. Any future research on residual samples will be in research to inform management for cervical screening in the future and will be overseen by the YouScreen Chief Investigator. Results will not impact clinical management for women in the trial.

7.8 Follow up

7.8.1 Women who return a self-sample

7.8.1.1 Clinical Follow up

All women who return a self-sample will be passively followed up for the duration of the trial, to obtain data on subsequent cervical screening tests, referrals to colposcopy and/or diagnoses of CIN2+. At the end of recruitment, we have factored in 6 months follow up time to allow data collection for self-sample HPV positives. Individual participant clinical follow-up length for self-sample screen positives during the current protocol will depend on the point at which they are enrolled and will range from 6-18 months. This will be carried out using NHAIS and GP record data.

7.8.1.2 Passive Long Term follow up

After the trial has ended we will continue to passively observe those enrolled in the trial through their cancer screening, cancer registration and other health related records through central registries at NHS Digital and Public Health England. This will include depersonalised data recorded on participants' cervical cancer or CIN3 diagnoses, tumour and individual characteristics, their interactions with the NHS as part of their cancer pathway. Linkage between these datasets will be conducted using privacy conserving methods (OpenPseudonymiser) to minimise the transfer of personal confidential data. Where available, molecular data recorded by NCRAS will also be analysed to support analysis of the epigenetics of cervical cancer within the population.

This will enable us to carry out long term follow up analysis to assess "longterm" coverage at 13 months after the trial has ended and on CIN2+ and cervical cancer rates in women who return a self-sample at approximately 3 and 6 years after the trial has ended. As the YouScreen samples will be the only self-samples recorded in NHAIS over the trial period, it should be straightforward for NHS Digital to provide us with aggregate anonymous data on the numbers of relevant women screened in NHAIS. The precise mechanism for collecting longterm follow up data at 3 and 6 years is dependent on future funding and therefore will be decided at a later date. However, we will request permission from the participants to allow for passive long term follow up via the colposcopy hospital records (e.g. Cyres database) and if feasible and from the National Cancer Registration and Analysis Service (aggregate anonymous data).

7.8.2 All women offered a kit

7.8.2.1 Passive long term follow up

After the current trial has ended we also plan to collect follow up data on all women offered a kit from NHAIS to examine the impact of self-sampling on long-term coverage at 13 months and at approximately 3 and 6 years after the trial end (as a minimum we will examine long-term coverage at 13 months after last woman was sent/given a kit (NHAIS data and GP medical record data). This will allow us to assess any "nudge" effect that the offer of self-sampling has on conventional

screening uptake, and whether or not the women who uptake self-sampling would have come for screening in any case.

7.8.3 All eligible women

7.8.3.1 Passive long term follow up

We will also assess long-term coverage for all eligible women at 13 months and at approximately 3 and 6 years after the trial end (as a minimum we will examine long-term coverage at 13 months after last woman was sent/given a kit (NHAIS data and GP medical record data) . This will comprise aggregate anonymous data from NHAIS.

7.9 Withdrawal criteria

Participants will be free to withdraw from the trial at any time without giving reasons and without prejudicing their further treatment and will be provided with a contact point where they may obtain further information about the trial and the withdrawal process. If participants choose to withdraw, the trial team would include them in the assessment of self-sampling and self-sampling HPV test result uptake data for reporting. Participants will be made aware (as detailed in the patient information booklet) that any samples that have been taken as part of the trial will continue to be analysed and data retained in the project however no new data will be collected (except anonymous aggregate data for which consent is not required). Residual samples can be requested to be destroyed.

Throughout the duration of the trial, participants will be able to visit the trial small c website for full information including accessing up-to-date contact details and the process for withdrawing their consent for the trial.

7.10 End of Trial

The end of the active recruitment of the trial will be defined as either when a minimum of 10,000 kits have been returned or at 12 months after study start, whichever is earliest.

The trial will end once a minimum of 6 months of follow-up data (6 months from the last participant recruited) for women testing HPV positive on a self-sample have been received.

The Chief Investigator will inform the REC of the end of the trial within 90 days of its completion. A summary of the final research report will be sent to the REC within 12 months of the end of the trial

8 MEDICAL DEVICES

8.1 FLOQSwab 552C.80

Description: The FLOQSWAB 552C.80 is a self-vaginal swab with a marked breakpoint, manufactured by Copan Italia S.p.A.

CE Marking and intended use: The FLOQSwab 552C.80 is CE marked as a medical device Class IIa; licenced for use for self-collection of vaginal samples in home setting for HPV detection. As such the FLOQSwab will be used within its intended use in this trial.

Storage and shelf life: The FLOQSwab 552C.80 has a shelf life of 36 months and must be stored between 3-30 degrees Celsius.

Supply: Copan Italia will supply up to 45,000 swabs free of charge for the duration of the trial. As the self-sampling kits will be issued to women and GP practices from CFH Docmail, Copan will arrange supply to CFH Docmail at a frequency as agreed in the terms of the supply agreement. The number and frequency of shipments will depend on the availability of storage space at CFH Docmail.

Recall: In the event of a manufacturer recall; Copan Italia S.p.A will notify the King's CPTU trial team. The CPTU trial team will coordinate with CFH Docmail and all participating GP practices to organise recall of any unused swabs to the manufacturer.

8.2 Roche cobas 4800 System

Description: The cobas 4800 system is a complete end to end solution that integrates total nucleic acid isolation, automates PCR setup and performs real-time PCR. The cobas 4800 system consists of two linked instruments: the cobas x 480 for fully automated sample extraction and the cobas z 480 for fast and accurate real-time PCR.

CE Marking and intended use: The cobas 4800 System is CE marked as an in vitro diagnostic medical device. The system is intended to be used as a diagnostic or screening system providing sample preparation, amplification and detection of specific targets from human samples.

Supply: Health Services Laboratories will procure the cobas 4800 system from Roche. Roche will also supply at cost consumables for 10,000 (+/- 10%) samples.

8.2.1 Roche cobas 4800 HPV Test

Description: The cobas® 4800 Human Papillomavirus (HPV) Test is a qualitative in vitro test for the detection of Human Papillomavirus in patient specimens. The test utilizes amplification of target DNA by the Polymerase Chain Reaction (PCR) and nucleic acid hybridization for the detection of 14 high-risk (HR) HPV types in a single analysis.

The test specifically identifies HPV16 and HPV18 while concurrently detecting the other high-risk types (31, 33, 35, 39, 45, 51, 52, 56, 58, 59, 66 and 68) at clinically relevant infection levels. Specimens are limited to cervical cells collected in Roche Cell Collection Medium (Roche Molecular Systems, Inc.), cobas® PCR Cell Collection Media (Roche Molecular Systems, Inc.), PreservCyt® Solution (Hologic Corp.) and SurePathTM Preservative Fluid (BD Diagnostics-TriPath). Manufactured by Roche Molecular Systems, Inc.

CE Marking and intended use:

The cobas 4800 HPV Test is CE marked as an in vitro diagnostic medical device (according to the In-Vitro Diagnostic Medical Devices Directive 98/79/EC).

Indications for use of the cobas® 4800 HPV Test are:

(a) The cobas® 4800 HPV Test is indicated for use in screening patients with ASC-US (atypical squamous cells of undetermined significance) cervical cytology results to determine the need for referral to colposcopy.

(b) The cobas® 4800 HPV Test is indicated for use in screening patients with ASC-US cervical cytology results to assess the presence or absence of high-risk HPV genotypes 16 and 18.

(c) The cobas® 4800 HPV Test is indicated for use adjunctively with cervical cytology to assess the presence or absence of high-risk HPV types.

(d) The cobas® 4800 HPV Test is indicated for use adjunctively with cervical cytology to assess the presence or absence of HPV genotypes 16 and 18.

(e) The cobas® 4800 HPV Test is indicated for use as a first-line primary screening test to identify women at increased risk for the development of cervical cancer or presence of high-grade disease.

f) The cobas® 4800 HPV Test is indicated for use as a first-line primary screening test to assess the presence or absence of HPV genotypes 16 and 18.

As such the cobas 4800 HPV test will be used **outside** of its licenced indication for the trial and will be considered to be exempt from the directive under the 'in-house exemption' to the Medical Devices Regulations 2002. Full details can be found in section

Supply: Health Service Laboratory will procure the cobas 4800 HPV test and associated reagents from Roche for analysis of 10,000 (+/- 10%) samples.

9 STATISTICAL CONSIDERATIONS

9.1 Sample size considerations

This is a feasibility trial of the implementation of a new self-sampling pathway that is not designed to detect a difference between two arms. Rather, to estimate the increase in coverage associated with offering self-sampling to nonattenders, therefore, formal power calculations are not appropriate. In

addition, the trial aims to recruit as many women as possible that the budget will allow. Our budget allows for a return of approximately 10,000 samples and issuing of circa 31,000 kits.

According to the NHAIS 2017/18 data available online from the National General Practice Profiles (https://fingertips.phe.org.uk/profile/general-practice), across the five CCGs included in our trial there are 212 GP practices and 190,579 women who are ≥6 months overdue for cervical screening. Assuming 50% (106/212) of GP practices agree to take part, there would be approximately 95,000 (0.50*190,579) eligible women (i.e. women aged 25-64 eligible for cervical screening who are overdue by at least 6 months).

9.1.1 Direct Mail-out

Assuming 106 GP practices recruit women for an average of 9 months (GP practices will recruit for a minimum of 6 months and a maximum of 12 months given that study set-up at each practice will be staggered over a 6 month period) and 20 women reach the 15 month anniversary at each practice each month, approximately 19,000 women will be sent kits during the trial. Table 2 below provides the expected 95% confidence intervals for various uptake levels over an average of 9 months recruitment period. We have estimated that 15% of women who receive a kit via direct mail-out will return a self-sample (i.e. ~2,800 women will return a self-sample over an average of 9m).

Table 2 Expected 95% confidence intervals for different proportions of women who return a self-sample based on 106 GP practices over a 9-month average recruitment period

Uptake among those offered kits	95%CI
10%	9.6-10.4
15%	14.5-15.5
20%	19.4-20.6
30%	29.3-30.7

9.1.2 Opportunistic offering of self-sampling

With an estimated 106 GP practices participating, there would be some 95,000 (190,579*(106/212)) women eligible for opportunistic offering of kits. Based on our previous studies(16, 37)) we estimate that ~60% (57,000) will consult their GP practice over 9-12 months, some 17,100 (30%) will be offered a self-sampling kit and of those offered, ~12,000 (70%) will accept. We estimate that a total of ~8,500 would return a self-sample for HPV testing (50% of those offered i.e. 17,000 *0.5). Therefore, the proportion of women ≥6m overdue cervical screening who will return a sample when opportunistically offered a kit (i.e. ≈8,500/17,000) could be estimated with a 95% confidence interval of width ±1.0%.

9.1.3 Questionnaire-based sub-study

As the trial is designed to be descriptive, a formal power calculation is not possible. A previous study in Westminster, London(18) had a survey response rate of 97% among the 6% of women who completed a self-sample kit, and just under 1% for those who did not send back a kit. We therefore expect a very low response rate in women who do not return the kit, making statistical comparisons between completers and non-completers inappropriate. However, we plan to explore demographic patterns of PAPM stage, previous barriers and confidence in completing the kit (e.g. by ethnicity and level of deprivation) within the completer and (to a lesser extent) the non-completer groups, and to do this, we anticipate needing a relatively large sample.

We have allowed for a high response rate (90% for kit completers; 10% for non-completers) to avoid under-funding. This would yield a sample of ~5,500 kit completers and ~2,500 non-completers but we anticipate the actual sample size is likely to be somewhat lower.

Table 3 Summary of anticipated sub-study sample

Total questionnaires sent:	31,000
Total returned from kit completers (90%):	5,580

Total returned from kit non-completers (10%):	2,480
Total questionnaires returned	8,060

9.2 Planned analysis

Overall baseline characteristics of individuals and GP practices will be presented overall and by pathway. We will identify the total number of women identified for the mail out cohort and the GP opportunistic pathway. We will also report on the cervical screening status at the end of the passive follow up period (aggregate data by GP practice).

We will compare individual characteristics for women who consent to take part in the trial vs. those who do not. We will also tabulate primary and secondary outcomes overall and by pathway.

A detailed description of analysis will be provided in the statistical analysis plan to be finalised prior to completion of data collection.

9.2.1 Primary endpoint

The primary outcome is uptake which is defined as the proportion of women who return a self-sample at 30 days, 90 days and at the end of the trial amongst those who have been given one direct mail out and opportunistic offer at GP practice with adjustment for multiple testing. We are interested in estimating variability across GP practice by using funnel plots(38) and estimating the intra cluster correlation (ICC).

9.2.2 Secondary outcomes – individual level

One of the secondary aims to is to compare uptake by pathway, ethnic group, LSOA and age. This will be achieved using mixed-effect models with random effect for clusters (GP practice) with uptake regressed onto the predictor of interest while and adjusting the model for time. This will allow us to estimate the ICC and the time effect. We will use this approach to analyse the other binary secondary endpoints collected at individual level:

- The proportion of women who attend for follow up within 6m (cervical cytology, HPV primary test or colposcopy)
- The proportion with abnormal follow up results (cytology and/or HPV primary)
- The proportion with CIN2+ and CIN3+ on histology (also expressed as proportion of all women with a self-sample by age)
- The proportion of women whose self-sample is unavailable (insufficient DNA), and of these:
  - who return a second self-sample or attend for clinician sampling

9.2.3 Secondary outcomes – GP practice level

Based on NHAIS data, we will explore change (before vs. after) in cervical screening coverage. We will explore variation across GP practice using Funnel plots.

We will run a Mantel-Haenszel test accounting for clustering at the GP practice level to explore the relationship between coverage and before/after.

We will also explore whether coverage varies as a function of age group, screening history, ethnic group and LSOA using change in cervical screening coverage using the extended Mantel-Haenszel test for 2*j tables so to account for the clusters.

Change in cervical screening coverage at each practice (to assess the impact of self-sampling on coverage):

- Before (including both immediately before the GP practice is “entered” into the trial and exactly one year before the end of the trial) and after the trial.
- Coverage in each month prior to and post the trial starting at the GP practice.

9.2.4 Questionnaire-based sub-study

Descriptive analyses will be carried out in SPSS. Where sample size allows, the distribution of responders across the PAPM stages, and the self-reported barriers to previous participation will be

compared across demographic sub-groups (by ethnic group, age, and deprivation). Separate analyses will be carried out for kit completers and non-completers.

10 DATA MANAGEMENT

10.1 Data collection tools and source document identification

10.1.1 Source data

Source data for the trial will comprise the questionnaire for the sub-study, GP records, laboratory records, hospital records (colposcopy data held in Cyres) and extracts from NHAIS (national screening database).

The questionnaire will be in paper or electronic (via REDCap) format and will be entered into the YouScreen database.

The remaining source data are all in electronic format that will extracted directly from the original databases.

10.2 Data handling and record keeping

All data transferred between stakeholders, or to KCL will be covered by appropriate data sharing or data processing agreements.

All data provided to or from CFH Docmail will be done so via their HSCN Secure File Transfer (HSCN SFTP). This will include the monthly data extract from CSAS, any ad-hoc repeat kit requests from HSL, and details of undelivered letters or self-sampling kits.

Trial data will be stored securely and made available for audit according to the standard procedures of King's College London. Third party companies will process data on behalf of King's College London including the provider for the Data Safe Haven (DSH) (currently this is AIMES https://www.aimes.uk/) and the commercial print company (CFH Docmail).

A YouScreen trial database will be built in RedCap (a secure web application for building and managing online surveys and databases). The RedCap trial database will be kept in a DSH at King's College London. The DSH is a controlled and secured service environment for undertaking research using sensitive data (personal, sensitive-personal, or confidential). The service provides robust controls and safeguards to enable the secure transfer of sensitive data into a highly secure environment where it can be stored, manipulated and analysed by approved members of a research team. Only designated members of the YouScreen trial team will have access to the study database and the Data Safe Haven.

Data transferred/saved in the DSH at King's College London, will be cleaned/queried/checked for missing data.

The final dataset will be exported and analysed in STATA or R: the RedCap database and .csv files will be imported into a statistical programme (e.g. STATA) for final data cleaning and analysis.

10.3 Study ID

The study/participant ID will comprise the encrypted NHS number and will be used to de-personalise the data and to link between seven data sources (see section 10.4). This will enable us to bring together all of the data required for analysis to meet the trial objectives.

For the encryption we plan to use the free open source software OpenPseudonymiser (https://www.openpseudonymiser.org/Default.aspx). OpenPseudonymiser allows an encrypted "salt" (an extra string of characters appended to the data being de-personalised) to be added, which ensures that there is no risk of the data being cross referenced by another project that uses different salt. The encryption programme and salt will be created by an individual outside of the King's trial team at Public Health England. The encryption programme and salt will be provided to each of the bodies who will encrypt NHS numbers for the trial. These are: HSL laboratory, participating CCGs and NHS Digital (NHAIS data).

The King's trial team will not have access to the encryption programme at any point and will not be able to decrypt the information. As such, the data held at King's College London will be considered to be effectively anonymous in line with the ICO's anonymisation standard (but will still be stored securely in the DSH).

10.4 Datasets

Trial data will be collected from seven different sources:

(i) NHAIS database,
(ii) GP medical records
(iii) The central NHS laboratory (HSL),
(iv) Hospital record (colposcopy clinic) data from the Cyres database (Cyres has a central server database where all cytology and histology data from NHS colposcopy clinics in London are uploaded),
(v) The mailing company (CFH Docmail) and
(vi) The questionnaire, and
(vii) Cancer registration data from the National Cancer Registration and Analysis Service.

A further dataset may arise from the analysis of residual samples; however, this will be treated separately from the main dataset (i.e. to be analysed and reported separately). Data arising from the residual samples may be annotated with cancer registration data for future translational research. Linkage to cancer registration data will be conducted using privacy conserving methods as described in 10.3.

The data (csv or excel files) will be uploaded to King's CPTU using a secure electronic transfer method. Once a data file has been downloaded at the King's CPTU and formatted for the YouScreen database by the Data Coordinator or Trial Coordinator it will be uploaded onto the YouScreen database which will be stored in the DSH.

10.4.1 NHAIS dataset

NHAIS holds the national cervical screening database and is therefore, the most accurate and complete source of screening data. GP records also contain cervical screening data, however, this tends to be less accurate than NHAIS as the two systems are not linked. As such, NHAIS data will be the main source of cervical screening data for the trial

Prior to each data extraction NHS Digital will apply the "exclusion" lists to remove women in the national data opt-out and women who have opted out from data usage for the study (see section 7.3.5). NHS Digital will then extract the data and, prior to sending it to KCL, will encrypt the NHS numbers (i.e. generate study ID) and convert the postcode to LSOA. In this way only anonymous line-level data will be provided to the trial team at King's for all eligible women (i.e. ≥6m overdue cervical screening) and therefore, will include data from women who have not consented to the trial. We will seek CAG approval for this. Data will include:

- Study ID (encrypted NHS number)
- Demographic data (age, LSOA (converted from postcode prior to sending to KCL))
- Cervical screening tests (including self-samples, cytology and HPV tests) dates and results, source of self-sample (opportunistic or mailout) including the last test before trial start, all tests during the trial period)
- Date identified for 15m mail out (if applicable)
- Cervical screening disclaimer returned (i.e. opted-out from the cervical screening programme)
- The woman's GP practice

These line-level NHAIS data will be linked to anonymised line-level data from GP records using the study ID to enable us to assess response rates and cervical screening activity according to ethnicity, whether or not women are offered kits in GP primary care.

We will also collect anonymous aggregate data:

- Practice-specific coverage at before study start, monthly and at the end of the trial

- An additional extract on practice-specific coverage may be requested after the trial has ended (after approximately 13 months after the last kit was handed out) to assess long term coverage.

10.4.2 Central Laboratory dataset

The central laboratory (HSL) will provide detailed data on self-samples that are received at the laboratory for the trial. Although self-sample results will also be obtained from NHAIS, the laboratory will provide critical data on self-samples that will allow us to assess the self-sampling pathway that are not available in NHAIS such as sample received date, analysed date, samples with missing or incorrect information, HPV type, kit ID numbers, and consent status for residual samples (on the lab request/consent form).

The HSL laboratory is the central NHS laboratory for cervical screening in London, therefore have existing permission to receive full PID and will handle data securely to NHS standards.

HSL will encrypt the NHS numbers prior to sending the data to the trial team at King's. De-personalised line-level data will be provided for women who return a self-sample.

Anonymous aggregate data will be collected for:

Number of samples rejected because:

- DNA insufficient (unavailable) – β-globin negative
- Unlabelled sample or missing lab request/consent form
- Damaged in transit
- Identifiers not matched on lab request/consent form and sample tube
- Sample received outside of agreed validity window
- Self-sample already recorded in central laboratory LIMS database
- Woman ineligible for self-sampling because not >=6m overdue cervical screening

10.4.3 Hospital data (colposcopy clinic data from Cyres)

Cyres is the integrated software that is used by all colposcopy clinics/services in London to generate routine reports (KC65) on referrals to colposcopy, subsequent treatment and outcome, for the cervical screening programme. As the designated London central laboratory for the NHSCSP, HSL are able to access colposcopy data in Cyres for all of London.

HSL will extract any data that are available in Cyres for all women who return a self-sample (downstream events only). Data will include referrals to colposcopy, date of colposcopy, histology date and results of biopsy or treatment (if applicable).

Data will be extracted from Cyres then merged to the main central laboratory dataset using NHS number. HSL will encrypt the NHS numbers (i.e. generate study ID) so that all datasets provided to the King's trial team will be anonymised.

10.4.4 GP record datasets

Electronic GP record data will provide key data for the trial that are not available from the other data sources, such as ethnicity, granular details of the opportunistic offer (kits offered, accepted and declined, reasons for declining kits, and health professional type offering kits).

Cervical screening (dates and results) data will be obtained to enable us to assess how accurate GP records are for identifying women eligible for the opportunistic offer (it may be that kits may be offered to women who are ≥6m according to their GP records but not in NHAIS. Also, cervical screening data in GP records may contain details of tests taken in other countries which are not recorded in NHAIS.

EMIS searches will be written to exclude women who are coded to have opted out from data usage for the study (see section 7.3.5).

GP record data will be extracted in bulk using EMIS searches that have been specially written for the trial. Extracts are likely to be downloaded from participating GP practices once they sign up to the trial (historical cohort data), at the end of the trial and depending on logistics, at several points during the study (e.g. on a monthly basis, at the end of active recruitment). These data extracts will be downloaded centrally at the CCG level by a member of the relevant CCG. If central download is

not possible, it will be carried out by each GP practice by a member of the practice team. The Clinical Effectiveness Group (CEG) have confirmed that they have centralised access to all GP practice data in Tower Hamlets and Newham CCG. Similarly, North East London Commissioning Support Unit (NEL CSU) have access to data from all GP practices in Barnet and Islington CCGs. Camden CCG have an IT lead who we plan to liaise with to extract data for Camden GP practices.

De-personalised line-level data will be collected for all women who are eligible (i.e. >=6m overdue cervical screening) and therefore, will include data from women who have not consented to the trial.

CAG approval will be sought to allow download of identifiable data at the CCG level and to allow the trial team at KCL to access de-personalised line-level data from women who have not consented to the trial. The CCG IT team will encrypt the NHS numbers before securely sending the data to King's.

We plan to use the in-built template and search functions within EMIS web to collect standardised data from GP records. Only anonymous aggregate data or line-level de-personalised data will be accessed by the trial team.

10.4.5 Mailout / Print company dataset

The commercial print company (CFH Docmail) will be handling data on behalf of King's College London. They will provide de-personalised (de-personalised using study ID which they will receive from NHAIS via CSAS) line-level data on the dates kits are sent out (primary endpoint data), kit numbers and aggregate anonymous data on numbers of kits returned (undelivered).

10.4.6 Questionnaire dataset

Questionnaires will be returned to the King's trial team directly from women via the post. They will be pre-printed with the kit ID number. The kit ID number will be used to link questionnaire answers to the woman's cervical screening history, HPV result and (if HPV positive) attendance for follow-up, as well as basic demographic information not collected as part of the questionnaire e.g. age, LSOA. Kit ID numbers with corresponding study ID numbers (encrypted NHS number) will be provided by HSL and CFH Docmail.

Data collected in the questionnaire include self-reported data on previous screening barriers and future screening preferences.

10.4.7 NCRAS data

Anonymous aggregate data from the National Cancer Registration and Analysis Service (NCRAS) may be requested at approximately 3 and 6 years after the trial has ended for women who return a self-sample. Data will comprise numbers of women with CIN3 and cervical cancer.

10.5 Confidentiality

Only anonymised and de-personalised data (with study ID) will be collected by the trial team for analysis. The trial has been designed to ensure that only minimal identifiers are used (while maintaining data accuracy and integrity).

All information related to participants will be kept confidential and managed in accordance with General Data Protection Regulation (GDPR), NHS Caldicott Principles, UK Policy Framework for Health and Social Care Research (2017), and the conditions of Research Ethics Committee Approval. Data for the trial will be shared via secure NHS email or a secure data sharing platform.

The identification of women in NHAIS (national screening database) for the direct mail out cohort will be carried out using public authority under the NHS Screening Programmes as the lawful basis (PHE are the current joint data controllers).

KCL will also be including lab request/consent forms for women should they choose to partake in the trial using the residual samples collected afterwards and separately to the samples being screened for the Public Health purpose. As the personal data are being used for the secondary purpose of including the invitation to partake in the trial then NHSE cannot be the sole data controller, therefore KCL will become the data controller once the data have been released from NHAIS. A Data Sharing Agreement between PHE (current data controller) and KCL (data controller) will be in place. In addition, both KCL and PHE will complete a DPIA (Data Protection Impact Assessment).

Completed lab request/consent forms will be retained by the laboratory until the end of the study when they will be archived according to the sponsor arrangements detailed in Section 10.6.

Appropriate agreements will also be put in place between UCL, KCL, NHAIS, the laboratories, the commercial print company and the participating GP practices, as necessary in order to ensure confidentiality and appropriate data handling.

10.6 Record Retention and Archiving

The commercial print company (CFH Docmail) will retain identifiable data from NHAIS (via CSAS) for one month from the date of receipt. The rationale for allowing one-month retention is that this is a new and un-tested pathway and we anticipate that there may be snagging issues. Therefore, we have factored in sufficient time for CFH Docmail to ensure that all of the required letters and kits have been sent to the relevant women each month, including the timely reissue of kits and letters if necessary. All PID held will be securely purged from the CFH Docmail systems every 30 days. CFH Docmail will maintain a master list of the study IDs (encrypted NHS numbers), kit numbers and date of kit/letters sent for the mailout offer for the duration of the trial as these data will be used to assess the primary endpoint of kit uptake and for audit and reporting purposes.

Trial data will be stored securely and made available for audit. Upon completion trial records will be kept for 20 years as per UK Policy Framework for Health and Social Care Research (2017) and UCL Records Retention Schedule. Archiving will be authorised by the Sponsor following submission of the end of study report. All essential documents will be archived for at least 20 years after end of study. Principal Investigators/Local Collaborators are responsible for the secure archiving of essential documents as per their local policy. Destruction of essential documents will require authorisation from the Sponsor.

11 SAFETY REPORTING

11.1 Definitions of Adverse Events

Term	Definition
Adverse Event (AE)	Any untoward medical occurrence in a patient or study participant, which does not necessarily have a causal relationship with the procedure involved.
Serious Adverse Event (SAE).	Any adverse event that:<br>• results in death,<br>• is life-threatening*,<br>• requires hospitalisation or prolongation of existing hospitalisation**,<br>• results in persistent or significant disability or incapacity, or<br>• consists of a congenital anomaly or birth defect
*A life- threatening event, this refers to an event in which the participant was at risk of death at the time of the event; it does not refer to an event which hypothetically might have caused death if it were more severe.<br>** Hospitalisation is defined as an in-patient admission, regardless of length of stay. Hospitalisation for pre-existing conditions, including elective procedures do not constitute an SAE.	

11.2 Causality

The assessment of relationship of adverse event to the study is a clinical decision based on all the information available at the time of the event.

The following categories will be used to define the causality of the adverse event:

Category	Definition
Definitely:	There is clear evidence to suggest a causal relationship, and other possible contributing factors can be ruled out.
Probably:	There is evidence to suggest a causal relationship, and the influence of other factors is unlikely

Possibly	There is some evidence to suggest a causal relationship. However, the influence of other factors may have contributed to the event (e.g. the participant's clinical condition, other concomitant events).
Unlikely	There is little evidence to suggest there is a causal relationship. There is another reasonable explanation for the event (e.g. the participant's clinical condition).
Not related	There is no evidence of any causal relationship.
Not Assessable	Unable to assess on information available.

11.3 Expectedness

Category	Definition
Expected	An adverse event which is consistent with the information clearly defined in this protocol.
Unexpected	An adverse event which is not consistent with the information clearly defined in this protocol.

11.4 Recording adverse events

Self-sampling is a low risk intervention and flocked swabs for vaginal self-collection are already widely used within the NHS. We do not anticipate any major safety issues based on previous studies and experience. As such, Adverse Events (AEs) will not be reported or collected.

11.4.1 Expected adverse reactions

There is a very small risk that the swab could break off in the woman's vagina during sampling as there is a breakpoint at 8cm from the swab tip. The participant information booklet outlines instructions to the woman to report this to her GP. The GP will be instructed at the Site Initiation Visit (SIV) to report any instances of swab breakages to the trial team via the generic trial email or telephone.

11.4.2 Serious Adverse Events

If a GP Practice is informed by a woman about an adverse event that meets the criteria of serious (section 11.1), the SAE should be reported to the trial team at KCL CPTU (on behalf of the sponsor) within 24 hours using the Trial Specific SAE form to the generic trial email address: youscreen@kcl.ac.uk.

All GP Practices will receive training at the SIV on the process for reporting SAEs.

11.5 Urgent safety measures

The CI will take urgent safety measures if necessary, to ensure the safety and protection of participants from immediate risks to their health and safety. The measures will be taken immediately. The approval of the REC prior to implementing urgent safety measures is not required. However, the CI will inform the sponsor and Research Ethics Committee (via telephone) of this event immediately.

The CI will inform the REC in writing within 3 days, in the form of a substantial amendment. The sponsor will be sent a copy of the correspondence.

11.6 Annual Safety Reporting

The CI will include safety information (a summary of reported SAEs) in the Annual Progress Report.

11.7 Overview of the Safety Reporting responsibilities

The CI has overall responsibility on behalf on the sponsor for safety reporting. The CI will ensure that safety monitoring and reporting is conducted in accordance with the sponsor's requirements.

12 MONITORING, AUDIT & INSPECTION

12.1 Assessment and management of risk

A formal risk assessment has been undertaken for the trial to identify the main risks and to propose mitigation strategies for these risks to ensure safe and successful delivery of the trial to the agreed milestones. Key risks have been outlined below.

12.1.1 Risk to participants

Vaginal self-sampling using flocked swabs has been safely performed in several studies(18, 39-42) and is already being used in the Australian national cervical screening programme (http://www.cancerscreening.gov.au/internet/screening/publishing.nsf/Content/self-collection-and-the-cervical-screening-test). The sampling procedure itself is already used for self-collection of high vaginal samples using a swab for Chlamydia testing in GP primary care. Women do not need to touch their cervix with the device.

Self-sampling has been shown to increase uptake among cervical screening non-attenders and being screened confers protection against developing cervical cancer. Self-sampling gives women greater choice in screening and addresses most screening barriers by enabling women to take a test for cervical screening themselves without having to be examined or to make an appointment.

There is a very small risk that the swab could break off in the women's vagina during sampling as there is a breakpoint at 8cm from the swab tip. However, significant bending of the swab is required for this to happen (much more than applied during sample-taking) and we have been informed that there have been no reported incidences of this happening within the Australian national programme (~3000 samples) or within a Malaysian pilot study (~4000 samples). In our previous and ongoing self-sampling studies using the same flocked swab (>800 samples) this has not occurred and no issues with sample collection have been reported by the women.(16) If in the unlikely event that this does happen the woman should be able to remove the swab easily herself as the breakpoint is at approximately 8cm and will be easily accessible.

Women will be provided with pictorial and written instructions for taking their sample including the best position to get into to take their sample and an instruction to hold the swab on the shaft where the breakpoint is. This should minimise the risk of any sample taking which would put undue force or bending of the swab. At the site initiation visits GP practice staff will receive training on the possibility accidental breakage of the swab shaft, how to manage this if reported by the women and how to report incidents to the YouScreen coordinating team. Swabs that are removed without touching the tip will be considered viable, otherwise a new kit will be given to the woman. In addition, any incidences of breakage occurring will be monitored by the Study Delivery Group and escalated to the Joint Steering Group as necessary.

12.1.2 Risk to trial delivery

The main risks to successful trial delivery stem from the various large pieces of development work that are needed to carry out the trial within the NHS CSP. These comprise (i) the programming changes to the national screening database (NHAIS) to record self-samples and reset the NTDD and (ii) set up and local validation of the Roche cobas HPV assay in the central laboratory (as the cobas platform is not currently in use in Health Services Laboratory). We have factored in realistic timelines to allow sufficient time for these changes to be made and tested. We have regular meetings with the relevant parties to ensure progress is on track ahead of the planned trial start.

As outlined in section 10, this trial involves the collection of data from numerous different sources and stakeholders and subsequent linkage of this data. As such, the transfer of both patient identifiable data and de-personalised data between sources, and to KCL is a significant risk to ensure appropriate handling of data. This risk will be minimised by clear arrangements for data sharing and transfer via data sharing/transfer agreements, agreed, authorised and tested procedures in place with each stakeholder for data transfer and central monitoring of data quality by KCL.

Further risks to trial delivery include the logistical challenges associated with setting up the anticipated 106 GP practices for an intervention trial. We have built in contingencies to deal with levels of interest that are either over or under our estimate of 106 GP practices and for effectively setting up the trial at scale. We will draw on the experience and influence of the North Central and

East London Cancer Alliance to promote and encourage recruitment should we find demand for the study is lower than expected; however we understand that there is already a great deal of interest from GP Practices; therefore we may find the demand higher than the expected 50%. If this arises, we will consider the recruitment capability of each GP Practice carefully prior to initiating setup to ensure that our resources are directed to the GP Practices most likely to recruit well.

We have also planned contingencies for self-sample return rates that are either over or above our estimates or outside of the estimated timelines.

Based on our experiences with the ALOHA study (17/LO/1655) we have identified that the supply and personalisation of kits at scale by a commercial print company may pose a risk to trial delivery. However, given the issues with the supplier used in ALOHA we have opted to use a different supplier for the commercial print company who have much more experience with complex individualised mailouts within the NHS and in a research context. We have worked closely with the proposed supplier and specified a high degree of detail to ensure that our trial processes are well-specified, and we achieve smooth trial delivery.

12.2 Monitoring

A monitoring plan will be developed by the King's CPTU team for the trial from the completed risk assessment.

Monitoring of the trial will be conducted using a risk-based approach and follow a trial monitoring plan developed by the King's CPTU team through data review and site or vendor visits on an agreed frequency and schedule.

The following areas may be reviewed as part of trial monitoring:

- Participant enrolment, consent and eligibility

- Adherence to trial interventions and policies to protect participants, including reporting of harm

- Completeness, accuracy, and timeliness of data collection

- Kit accountability and handling

- HTA compliance

Sites flagged for monitoring would be those with the highest enrolment rates, large numbers of withdrawals or low uptake numbers, or any event which in the study team or Sponsor's opinion should trigger a visit. Sites/vendors would be required to accommodate monitoring visits by providing access to relevant staff, premises and records. Monitoring findings will be reported to the Chief Investigator, Joint Steering Group as required and notified to the Sponsor.

Further details will be elaborated upon in the Trial Monitoring Plan.

The trial may be audited by UCL and KCL CPTU.

13 TRIAL COMMITTEES

Three trial oversight groups and committees will be set up for the trial: (i) a study delivery group (ii) a joint steering group and (iii) an independent trial oversight committee.

13.1.1 Study Delivery Group

The study delivery group will meet fortnightly or monthly (depending on the level of study) and will comprise representatives from NCELCA, and KCL CPTU who are responsible for day to day management of the trial and carrying out the work detailed in the trial protocol. The purpose of the group is to:

Ensure the trial is on track to its project plan and budget.
Manage trial risks, issues and mitigations, escalating any major risks and issues to the NCELCA Programme Director and trial steering groups as necessary.
Provide and maintain ongoing progress reports to the Joint Steering Group and NCELCA Board.

The Study Delivery Group will hold biweekly progress update meetings with NHS Digital to oversee the development of HPV Self-sampling in NHAIS. This group will monitor whether the recording of

self-samples in NHAIS for the trial causes any “safety” issues. If major issues are identified, then the recording of self-samples in NHAIS would be suspended (until resolved) or stopped.

13.1.2 Joint Steering Group

A joint steering group has been set up to guide the trial protocol and provide expertise on the various aspects of the trial. The group comprises representatives from PHE, NHSE London, NHS Digital, CSAS, NCELCA, KCL, UCL, NHS laboratory, CCGs, GPs, patient representatives, Jo’s Cervical Cancer Trust. The purpose of the joint steering group is to advise and provide high-level strategic guidance to the study delivery group and other associated work streams, oversee implementation and delivery of the trial.

13.1.3 Independent Trial Oversight Committee

An independent trial oversight committee (ITOC) will be set up to ensure that the Trial is conducted to the rigorous standards set out in the Medical Research Council's (MRC) Guidelines for Good Research Practice. The ITOC will concentrate on progress of the trial, adherence to the protocol, patient welfare and to assess the impact and relevance of any new information of relevance to the research question. The ITOC will provide advice, through its chair, to the Joint Steering Group Chair, Chief Investigator (CI), Trial sponsor, Trial funders, King’s and any other relevant party on all appropriate aspects of the Trial. The majority of members of the ITOC, including the Chair, will be independent of the Trial. Non-independent members will also be part of the ITOC (Chair of Steering Committee and the CI) as observers. Representatives of the trial Sponsor and the Trial funders may be invited to all ITOC meetings. The final decision regarding whether or not the trial may continue is the responsibility of the ITOC.

14 ETHICAL AND REGULATORY CONSIDERATIONS

14.1 Health Research Authority (HRA) & Research Ethics Committee (REC) approvals

The Chief Investigator will ensure that the protocol and supporting participant-facing documentation receive HRA Approval, including being presented to a relevant Research Ethics Committee for favourable opinion. Following ethical review, research will only take place once appropriate HRA approvals are in place. The trial team will prepare the Annual Progress Report on behalf of the Chief Investigator within 30 days of the anniversary date on which the favourable opinion was given, and annually until the trial is declared ended. They will also on behalf of the Chief Investigator:

- notify the REC of the end of the trial
- if the trial is ended prematurely, will notify the REC, including the reasons for the premature termination
- within one year after the end of the trial, will submit a final report with the results, including any publications/abstracts, to the REC

14.2 Confidentiality Advisory Group (CAG) approval

An application will be made to the CAG for section 251 approval to cover two key parts within the trial where it is not practicable to seek consent.

1) *Participant identification to support the recruitment of participants to a REC and HRA approved trial and to subsequently seek their consent*

The national cervical screening database NHAIS (National Health Application and Infrastructure Services) hold details of women eligible for the national cervical screening programme. As part of the national cervical screening programme, NHAIS sends details of women who are due their cervical screening to the Cervical Screening Administration Services (CSAS) who are commission to:

- provide Prior Notification Lists (PNLs) of patients eligible for screening to GP practices
- send out call and recall letters to patients eligible for cervical screening tests
- notify patients of test results once we receive these from laboratories

for the call/recall Cervical Screening Programme in England.

We will ask NHAIS to identify details of eligible women who are at their 15-month anniversary of their last test date without being screened, and send these details to the Cervical Screening Administration Services (CSAS) who are responsible for sending invitations and reminders for the call/recall Cervical Screening Programme in England.

At this point, the data for these women will leave the direct care pathway and be securely transferred to a to a third-party commercial print company contracted by KCL who will send out letters and kits for self-sampling. It is this sharing of personally identifiable data without consent to a commercial print company for which we are seeking Regulation 5 approval to set aside the common law duty of confidentiality.

Once the woman returns a sample to the laboratory, consent to take part in the trial will be implied.

2) *Time limited access to all patient records, including those who have not consented to participate in the trial, for provision of anonymised data to the trial team*

In order to meet the trial objectives, de-personalised line level data will be collected for all women identified as eligible i.e. including women who do not return a self-sampling kit or questionnaire. This data collection will happen in two separate ways; from the electronic GP record (centrally, at the CCG level) and the national cervical screening database, NHAIS, for all women who are eligible and therefore will include data for women who have not consented to the study.

a. GP Practice record extractions performed centrally via each CCG

Clinical Commissioning Groups are not permitted to have access to personal confidential data; as they are not providing direct patient care.

The application to CAG will therefore seek to permit the download of identifiable data at the CCG level via the NHS numbers (so that they may be encrypted to de-personalise the data and create the study ID) to allow the trial team at KCL to access de-personalised line level data from women who have not consented to the trial. It will also allow the CCG to download free text fields which we would like to include as “associated text” fields linked to the clinical codes for self-sampling kit “accepted” and “declined”. Input function will be limited in EMIS web to ensure only relevant data are entered (i.e. use of drop-down menus).

The GP data will comprise key data that are not available from other data sources, such as ethnicity, granular details of the opportunistic offer (kits offered, accepted and declined, reasons for declining kits, health professional type offering kits) and cervical screening tests that may not be recorded in NHAIS (e.g. those taken in other countries).

These data are currently not covered by the existing section 251 approval held by the CCGs for audit purposes and are crucial to the study endpoints (impact of self-sampling on inequalities). This will provide important data on the profile of women who do not respond to the self-sampling offer.

b. Data extracted from the NHAIS dataset

The NHAIS data will give details of cervical screening test/result history and date identified for mailout (if applicable). This line level data will be linked via the study ID will enable us to assess response rates and cervical screening activity according to ethnicity and opportunistic offering. For women who return a self-sample consent is implicit. However, women who do not respond will not have provided their consent to use this data and therefore Section 251 approval will be required.

Opt-out mechanisms will be in place to enable individuals who do not wish for their data to be used. (See section 7.3.5 Opt-out procedures for women who do not consent to the trial).

The trial will not start until CAG approval has been received.

14.3 Medicines and Healthcare Regulatory Agency

The trial is not considered to be under the remit of the MHRA and as such we will not receive a Notice of No Objection. The Cobas 4800 HPV test will be used **outside** of its licenced indication for the trial and will be exempt from the directive under the ‘in-house exemption’ to the Medical Devices Regulations 2002.

14.3.1 In house exemption

The current EU medical device and in vitro diagnostic medical device Directives do not apply to devices manufactured and used only within the same health institution and on the premises of their manufacture or used on premises in the immediate vicinity without having been transferred to another legal entity (in-house use). Therefore, under the Directives, devices manufactured and used within health institutions are not considered as having been put into service and health institutions are exempt from the obligations set out in the Directives.

A health institution is defined as 'an organization whose primary purpose is the care or treatment of patients or the promotion of public health'. This includes hospitals, laboratories, local authorities and public health institutes supporting the health care system and/or addressing patient needs but may not treat or care for patients directly.

HSL will be using a Roche platform out of the CE mark and this is where the in-house exemption applies. The UCLH/UCL Joint Research Office (JRO)'s justification of being one health institution is as follows:

1) As an equity investor – UCLH own a set percentage of HSL and this is governed through the Joint Venture Agreement
2) As a customer – HSL supply all UCLH pathology services (aside from consultant histopathologists and some QS specialist labs). This is through a Pathology Services Agreement
3) The JRO is a joint unit between UCL and UCLH and under our processes this will be sponsored by UCL to meet insurance requirements.
4) The UCLH Cancer Program Director is designated the study leader and has overall responsibility for management of the study and its funding. The funding resides in UCLH.
5) A UCL sponsor representative and a UCLH clinician (Adam Rosenthal) will sit on the trial steering committee.

14.4 Other approvals

The trial will also seek Cervical Screening RAC approval prior to trial start. Approval for release of NHAIS data and access to data processed by the National Cancer Registration and Analysis Service will be obtained from the Office for Data Release (ODR).

14.5 Peer review

The protocol has received independent, expert, and proportionate reviews including review by a member of the UK National Screening Committee and head of the Department of Health Policy Research Unit for cancer screening and a leading UK gynaecologist.

14.6 Regulatory compliance

The Trial will not commence without the necessary regulatory and organisational approvals being in place. The Trial will have appropriate HRA Approval, NHS REC Favourable Opinion, CAG approval and confirmation of capacity & capability in place for each GP site.

The Trial Master File will hold all relevant communications with regulatory bodies and maintained by KCL.

14.7 Protocol compliance

Any accidental protocol deviations will be adequately documented on the relevant forms and reported on the trial deviation log and notified to KCL CPTU. All deviations from the protocol which are found to frequently recur will require immediate action and could potentially be classified as a serious breach. The CI will retain oversight of the trial deviation log.

Notification of Serious Breaches to GCP and/or the protocol: A "serious breach" is a breach which is likely to affect to a significant degree the safety or physical or mental integrity of the subjects of the Trial; or the scientific value of the Trial.

The Sponsor will be notified immediately of any case where the above definition applies during the trial conduct phase.

14.8 Data protection and patient confidentiality

All investigators and trial site staff will comply with the requirements of the Data Protection Act 2018 with regards to the collection, storage, processing and disclosure of personal information and will uphold the Act's core principles.

14.9 Sponsorship and Indemnity

University College London (UCL) will act as sponsor for this Trial.

UCL will delegate overall responsibility for the design, management and conduct of the trial implementation to the King's Cancer Prevention Trial Unit (King's CPTU) at KCL; in line with North Central and East London Cancer Alliance's (NCELCA) grant funding conditions. Access to data and Intellectual Property Rights will be governed by these grant conditions.

Delegation of contractual agreements with third parties will be discussed and agreed between UCL and KCL CPTU.

The NCELCA will act as funder and collaborative working partner with KCL for delivery of this project.

The Trial's Sponsor will ensure that appropriate insurance and indemnity arrangements are in place for the trial.

University College London holds insurance against claims from participants for injury caused by their participation in the clinical trial. Participants may be able to claim compensation if they can prove that UCL has been negligent. However, as this clinical trial is being carried out in a hospital, the hospital continues to have a duty of care to the participant of the clinical trial. University College London does not accept liability for any breach in the hospital's duty of care, or any negligence on the part of hospital employees. This applies whether the hospital is an NHS Trust or otherwise.

Participants may also be able to claim compensation for injury caused by participation in this clinical trial without the need to prove negligence on the part of University College London or another party. Participants who sustain injury and wish to make a claim for compensation should do so in writing in the first instance to the Chief Investigator, who will pass the claim to the Sponsor's Insurers, via the Sponsor's office.

GP Practices selected to participate in this clinical trial shall provide clinical negligence insurance cover for harm caused by their employees and a copy of the relevant insurance policy or summary may be provided to University College London, upon request.

14.10 Amendments

Trial amendments will be prepared by CPTU trial team according to local SOPs for submission according to Sponsor and HRA requirements. Protocol changes will be approved by UCL and/or CPTU prior to submission, and potentially by the Trial Joint Steering Committee depending on the nature of the amendment; as detailed in the TSC Charter.

It will be the Sponsor's responsibility to decide whether an amendment is substantial or non-substantial for the purposes of submission to regulatory bodies. KCL CPTU Trial team will be responsible for cascading amendments to participating sites.

Where the patient documentation provided in the self-sampling kits is amended and updated to a new version; Once the new versions are approved, the KCL CPTU Trial team will liaise with the commercial print company to distribute the new versions and ensure these are incorporated into the next kit run and to organise updated supply of kits to the GP surgeries. GP surgeries will be instructed to replace their stock of kits once the new delivery arrives.

14.11 Access to the final trial dataset

During trial implementation, the King's team will have controlled access to the full dataset. Wider data-sharing with follow the data-sharing and dissemination plans agreed with the funder.

15 PUBLIC AND PATIENT INVOLVEMENT

User representatives are involved in the trial design and review of patient-facing materials. We have recruited two patient representatives who sit on the Joint Steering Group. The patient representatives make a difference by ensuring that the approach is centred on the needs of patients and enabling us to think through processes from a patient perspective. As the trial will be held in GP primary care, we have involved GP leads from NEL and NCL in the steering group. Their role is to provide clinical advice on the implementation of the trial from a primary care perspective.

16 FINANCE AND FUNDING

Funding for the project will be provided by a grant awarded to KCL from the NCELCA as part of the transformation funding the Alliance receives from NHS England to deliver improvements in early and faster diagnosis and quality of life for people living with and beyond cancer.

17 DISSEMINATION POLICY

Data gathered from the trial will be collated and analysed by researchers at KCL. Summary reports of the main trial findings will be written and shared with the CCGs where the trial takes place, with Public Health England and NHSE. Findings will be published in peer reviewed scientific journals and online as well as used as evidence to propose a new policy on the use of HPV self-sampling for cervical screening non-attenders.

The trial will be registered on the ISRCTN registry.

17.1 Authorship guidelines

The final study report and related publications will be authored in line with the arrangements set out in the Collaborative Agreement dated 1st March 2019 and any subsequent variations or amendments. All publications will be approved by the Chief Investigator. Authorship will follow the criteria established by the International Committee of Medical Journal Editors.

18 APPENDICES

18.1 Appendix 1: Randomisation

If feasible, GP practices may be randomised to a start date using a computer programme. It is likely that a full list of participating GPs may not be available until very close to the trial start, which could make it logistically difficult for a timely trial set up (to book site initiation visits, install trial searches into GP software). GP practices may be randomised once a minimum of 20 have confirmed. A computer-generated list of random numbers will be produced by the trial team (KCL) using a statistical programme (e.g. STATA). A list of suitable start weeks or months will be identified and assigned to GP practices according to the assigned random numbers.

This will most likely only be done to the start of the opportunistic offer and if logistics allow.

18.2 Appendix 2: Revision history

Protocol version	Protocol date	Minor or substantial amendment	Date approved by Ethics	Date approved by HRA	Comments and description
V1.0	19.03.2020	Initial version	N/A	N/A	N/A

19 REFERENCES

1. Screening & Immunisations Team ND. Cervical Screening Programme, England - 2017-2018. 2018.

2. Public Health England (PHE) NE. Fingertips National General Practice Profiles. Females, 25-64, attending cervical screening within target period (3.5 or 5.5 year coverage, %) 2017/18. Cancer2019.

3. Marlow LAV, Chorley AJ, Haddrell J, Ferrer R, Waller J. Understanding the heterogeneity of cervical cancer screening non-participants: Data from a national sample of British women. Eur J Cancer. 2017;80:30-8.

4. Marlow LAV, Chorley AJ, Rockliffe L, Waller J. Decision-making about cervical screening in a heterogeneous sample of nonparticipants: A qualitative interview study. Psychooncology. 2018;27(10):2488-93.

5. Waller J, Bartoszek M, Marlow L, Wardle J. Barriers to cervical cancer screening attendance in England: a population-based survey. J Med Screen. 2009;16(4):199-204.

6. Arbyn M, Smith SB, Temin S, Sultana F, Castle P. Detecting cervical precancer and reaching underscreened women by using HPV testing on self samples: updated meta-analyses. Bmj. 2018;363.

7. Duffy SW, Myles JP, Maroni R, Mohammad A. Rapid review of evaluation of interventions to improve participation in cancer screening services. J Med Screen. 2017;24(3):127-45.

8. Snijders PJ, Verhoef VM, Arbyn M, Ogilvie G, Minozzi S, Banzi R, et al. High-risk HPV testing on self-sampled versus clinician-collected specimens: A review on the clinical accuracy and impact on population attendance in cervical cancer screening. Int J Cancer. 2013;132(10):2223-36.

9. Verdoodt F, Jentschke M, Hillemanns P, Racey CS, Snijders PJ, Arbyn M. Reaching women who do not participate in the regular cervical cancer screening programme by offering self-sampling kits: a systematic review and meta-analysis of randomised trials. Eur J Cancer. 2015;51(16):2375-85.

10. Polman NJ, Ebisch RMF, Heideman DAM, Melchers WJG, Bekkers RLM, Molijn AC, et al. Performance of human papillomavirus testing on self-collected versus clinician-collected samples for the detection of cervical intraepithelial neoplasia of grade 2 or worse: a randomised, paired screen-positive, non-inferiority trial. Lancet Oncol. 2019;20(2):229-38.

11. Arbyn M, Verdoodt F, Snijders PJ, Verhoef VM, Suonio E, Dillner L, et al. Accuracy of human papillomavirus testing on self-collected versus clinician-collected samples: a meta-analysis. Lancet Oncol. 2014;15(2):172-83.

12. Stanczuk G CH, Forson W, Palmer T, Cuschieri K. Women attending routine screening who test Hr-HPV negative on a self sample are at very low risk of disease over 5 years; lessons from the PaVDaG cohort EUROGIN; 04/12/2019; Monaco2019.

13. Nelson EJ, Maynard BR, Loux T, Fatla J, Gordon R, Arnold LD. The acceptability of self-sampled screening for HPV DNA: a systematic review and meta-analysis. Sex Transm Infect. 2017;93(1):56-61.

14. Williams D, Davies M, Fiander A, Farewell D, Hillier S, Brain K. Women's perspectives on human papillomavirus self-sampling in the context of the UK cervical screening programme. Health expectations : an international journal of public participation in health care and health policy. 2017;20(5):1031-40.

15. Lim AW, Sasieni P. Consultation rates in cervical screening non-attenders: opportunities to increase screening uptake in GP primary care. J Med Screen. 2015;22(2):93-9.

16. Lim AW, Hollingworth A, Kalwij S, Curran G, Sasieni P. Offering self-sampling to cervical screening non-attenders in primary care. J Med Screen. 2017;24(1):43-9.

17. Cadman L, Wilkes S, Mansour D, Austin J, Ashdown-Barr L, Edwards R, et al. A randomized controlled trial in non-responders from Newcastle upon Tyne invited to return a

self-sample for Human Papillomavirus testing versus repeat invitation for cervical screening. J Med Screen. 2015;22(1):28-37.
18. Szarewski A, Cadman L, Mesher D, Austin J, Ashdown-Barr L, Edwards R, et al. HPV self-sampling as an alternative strategy in non-attenders for cervical screening - a randomised controlled trial. Br J Cancer. 2011;104(6):915-20.
19. Council DH. Screening for cervical cancer—recommendations. 2017.
20. Australia MSAC. National cervical screening program renewal: executive summary. Effectiveness modelling and economics evaluation in the Australian setting. 2013.
21. Polman NJ, Snijders PJF, Kenter GG, Berkhof J, Meijer C. HPV-based cervical screening: Rationale, expectations and future perspectives of the new Dutch screening programme. Prev Med. 2019;119:108-17.
22. Wise J. DIY smear tests: are they the answer to falling cervical screening rates? Bmj. 2019;364:l361.
23. UK National Screening Committee. The UK NSC recommendation on Cervical Cancer screening in women. UK NSC coversheet and consultation responses2019.
24. Pedersen AF, Olesen F, Hansen RP, Zachariae R, Vedsted P. Social support, gender and patient delay. Br J Cancer (in press). 2011.
25. Hemming K, Haines TP, Chilton PJ, Girling AJ, Lilford RJ. The stepped wedge cluster randomised trial: rationale, design, analysis, and reporting. Bmj. 2015;350:h391.
26. Weinstein N, Sandman P, Blalock S. The precaution adoption process model. K Glanz BR, K Viswanath editor. San Francisco, CA, US: Jossey-Bass; 2008.
27. Chorley AJ, Marlow LA, Forster AS, Haddrell JB, Waller J. Experiences of cervical screening and barriers to participation in the context of an organised programme: a systematic review and thematic synthesis. Psychooncology. 2017;26(2):161-72.
28. Marlow LAV, Wardle J, Waller J. Understanding cervical screening non-attendance among ethnic minority women in England. British Journal of Cancer. 2015;113(5):833-9.
29. Waller J, Jackowska M, Marlow L, Wardle J. Exploring age differences in reasons for nonattendance for cervical screening: a qualitative study. BJOG. 2012;119(1):26-32.
30. Waller J, Robb K, Stubbings S, Ramirez A, Macleod U, Austoker J, et al. Awareness of cancer symptoms and anticipated help seeking among ethnic minority groups in England. British Journal of Cancer. 2009;101 Suppl 2:S24-30.
31. Hewitson P, Ward AM, Heneghan C, Halloran SP, Mant D. Primary care endorsement letter and a patient leaflet to improve participation in colorectal cancer screening: results of a factorial randomised trial. Br J Cancer. 2011;105(4):475-80.
32. Lam JU, Rebolj M, Moller Ejegod D, Pedersen H, Rygaard C, Lynge E, et al. Human papillomavirus self-sampling for screening nonattenders: Opt-in pilot implementation with electronic communication platforms. Int J Cancer. 2017;140(10):2212-9.
33. Hobbs FD, Bankhead C, Mukhtar T, Stevens S, Perera-Salazar R, Holt T, et al. Clinical workload in UK primary care: a retrospective analysis of 100 million consultations in England, 2007-14. Lancet. 2016;387(10035):2323-30.
34. Gok M, Heideman DA, van Kemenade FJ, Berkhof J, Rozendaal L, Spruyt JW, et al. HPV testing on self collected cervicovaginal lavage specimens as screening method for women who do not attend cervical screening: cohort study. Bmj. 2010;340:c1040.
35. Gok M, van Kemenade FJ, Heideman DA, Berkhof J, Rozendaal L, Spruyt JW, et al. Experience with high-risk human papillomavirus testing on vaginal brush-based self-samples of non-attendees of the cervical screening program. Int J Cancer. 2012;130(5):1128-35.
36. Wikstrom I, Lindell M, Sanner K, Wilander E. Self-sampling and HPV testing or ordinary Pap-smear in women not regularly attending screening: a randomised study. Br J Cancer. 2011;105(3):337-9.
37. Lim AW, Hollingworth A, Kalwij S, Curran G, Sasieni P. Offering self-sampling to cervical screening non-attenders in primary care. J Med Screen. 2016.
38. Spiegelhalter DJ. Funnel plots for comparing institutional performance. Statistics in medicine. 2005;24(8):1185-202.

39. Cerigo H, Coutlee F, Franco EL, Brassard P. Dry self-sampling versus provider-sampling of cervicovaginal specimens for human papillomavirus detection in the Inuit population of Nunavik, Quebec. J Med Screen. 2012;19(1):42-8.
40. Feng Q, Cherne S, Winer RL, Popov V, Zambrano H, Yerovi C, et al. Evaluation of transported dry and wet cervical exfoliated samples for detection of human papillomavirus infection. Journal of clinical microbiology. 2010;48(9):3068-72.
41. Wolfrum SG, Koutsky LA, Hughes JP, Feng Q, Xi LF, Shen Z, et al. Evaluation of dry and wet transport of at-home self-collected vaginal swabs for human papillomavirus testing. Journal of Medical Microbiology. 2012;61(Pt 11):1538-45.
42. Eperon I, Vassilakos P, Navarria I, Menoud PA, Gauthier A, Pache JC, et al. Randomized comparison of vaginal self-sampling by standard vs. dry swabs for human papillomavirus testing. BMC Cancer. 2013;13:353.
