# Supplementary material for: Opportunistic offering of self-sampling to non-attenders within the English cervical screening programme: a pragmatic, multicentre, implementation feasibility trial with randomly allocated cluster intervention start dates (YouScreen)
Source: eClinicalMedicine. 2024 Jul 16;73:102672. doi: 10.1016/j.eclinm.2024.102672 (PMC11490653; doi:10.1016/j.eclinm.2024.102672)
Supplement: Trial Steering Committee list [file mmc3.docx]

# Additional information

## Trial Steering Committee (Joint Steering Group)

| **Name** | **Role** |
| --- | --- |
| Miss Alexandra Lawrence | Co-Chair. North East London (NEL) and North Central London (NCL) Cancer Alliance London Cancer Gynaecology Pathway Director |
| Dr Clare Stephens | Co-Chair. NCL Cancer Alliance GP lead |
| Dr Afsana Bhuiya | NCL Cancer Alliance GP lead |
| Fanta Bojang | NCL Cancer Alliance Programme Manager |
| Catherine Nestor | NCL Cancer Alliance communications lead |
| Naser Turabi / Holly Norman | NCL Cancer Alliance Programme Lead |
| Kate Sanger | Jo’s Cervical Cancer Trust head of communications |
| Michelle Quaye /Farhat Gilani/Misha Ladva | UCL Sponsor representatives |
| Dr Anita Lim | YouScreen Chief Investigator King’s College London (KCL) |
| Prof Peter Sasieni | KCL |
| Dr Jo Waller | KCL – Reader in cancer behavioural science |
| Mairéad Lyons | King’s College London (KCL) – senior consultant to YouScreen |
| Jo Gambell | KCL project manager |
| Katie Deats | KCL Trial manager |
| Ann-Marie Wright / Aileen Masson | Department of Health and Social Care (DHSC) (formally Public Health England) National cervical screening programme (CSP) project manager |
| Philippa Pearmain | PHE Head of Screening Quality Assurance CSP |
| Ruth Stubbs | PHE CSP Programme Manager |
| Dr Josephine Ruwende | NHS England London Screening and Immunisations Lead (Cancer screening) |
| Hasit Patel | Cervical Screening London (CSL) |
| Mike Gandy | Cervical Screening London (CSL) |
| Paul Roberts | Cervical Screening Administration Service |
| Pauline Fisher | Cervical Screening Administration Service |
| Angela Lydon-Burgan | Cervical Screening Administration Service |
| Lorraine Silver | Patient representative |
| Kate Ruane | Patient representative |
| Nick Winfield | NHS Digital |
| Marion Dunn / Georgina Platt /Molly Taylor | NHS Digital |
| Adele Shepherd / Emma Coppini / Alison Cowie | NHS England Public Health Commissioning Central Team |
| Caroline Cook | NEL Cancer Alliance lead for screening |
| Claire Horner | NHS England |
| Elliann Fairbairn / Jo Aracena | Transforming Cancer Services Team |
| Wayne Douglas | WEL CCG representative – lead for cancer |
| Lucy McLaughlin | NCL STP representative (commissioning) |
| Gali Siegal | Cancer Research UK facilitator |
